# Recombination Rate Heterogeneity within Arabidopsis Disease Resistance Genes

**DOI:** 10.1371/journal.pgen.1006179

**Published:** 2016-07-14

**Authors:** Kyuha Choi, Carsten Reinhard, Heïdi Serra, Piotr A. Ziolkowski, Charles J. Underwood, Xiaohui Zhao, Thomas J. Hardcastle, Nataliya E. Yelina, Catherine Griffin, Matthew Jackson, Christine Mézard, Gil McVean, Gregory P. Copenhaver, Ian R. Henderson

**Affiliations:** 1 Department of Plant Sciences, Downing Street, University of Cambridge, Cambridge, United Kingdom; 2 Department of Biotechnology, Adam Mickiewicz University, Poznan, Poland; 3 Watson School of Biological Sciences, Cold Spring Harbor Laboratory, Cold Spring Harbor, New York, United States of America; 4 Institut Jean-Pierre Bourgin, INRA, AgroParisTech, CNRS, Université Paris-Saclay, RD10, Versailles, France; 5 The Wellcome Trust Centre for Human Genetics, University of Oxford, Oxford, United Kingdom; 6 Department of Biology and the Integrative Program for Biological and Genome Sciences, University of North Carolina at Chapel Hill, Chapel Hill, North Carolina, United States of America; 7 Lineberger Comprehensive Cancer Center, University of North Carolina School of Medicine, Chapel Hill, North Carolina, United States of America; UC Davis, UNITED STATES

## Abstract

Meiotic crossover frequency varies extensively along chromosomes and is typically concentrated in hotspots. As recombination increases genetic diversity, hotspots are predicted to occur at immunity genes, where variation may be beneficial. A major component of plant immunity is recognition of pathogen Avirulence (Avr) effectors by resistance (*R*) genes that encode NBS-LRR domain proteins. Therefore, we sought to test whether NBS-LRR genes would overlap with meiotic crossover hotspots using experimental genetics in *Arabidopsis thaliana*. NBS-LRR genes tend to physically cluster in plant genomes; for example, in Arabidopsis most are located in large clusters on the south arms of chromosomes 1 and 5. We experimentally mapped 1,439 crossovers within these clusters and observed NBS-LRR gene associated hotspots, which were also detected as historical hotspots via analysis of linkage disequilibrium. However, we also observed NBS-LRR gene coldspots, which in some cases correlate with structural heterozygosity. To study recombination at the fine-scale we used high-throughput sequencing to analyze ~1,000 crossovers within the *RESISTANCE TO ALBUGO CANDIDA1* (*RAC1*) *R* gene hotspot. This revealed elevated intragenic crossovers, overlapping nucleosome-occupied exons that encode the TIR, NBS and LRR domains. The highest *RAC1* recombination frequency was promoter-proximal and overlapped CTT-repeat DNA sequence motifs, which have previously been associated with plant crossover hotspots. Additionally, we show a significant influence of natural genetic variation on NBS-LRR cluster recombination rates, using crosses between Arabidopsis ecotypes. In conclusion, we show that a subset of NBS-LRR genes are strong hotspots, whereas others are coldspots. This reveals a complex recombination landscape in Arabidopsis NBS-LRR genes, which we propose results from varying coevolutionary pressures exerted by host-pathogen relationships, and is influenced by structural heterozygosity.

## Author Summary

The majority of plants, animals and fungi reproduce sexually, which has a profound effect on patterns of genetic diversity. For sexual reproduction to occur a specialized cell division called meiosis generates gametes (sex cells) with recombined DNA. During meiosis homologous chromosomes undergo reciprocal exchange of genetic material called crossover, which mixes parental sequences. Crossover frequency is highly variable along chromosomes and is typically concentrated in narrow regions called hotspots. Sexual reproduction is proposed to provide a major advantage during host-pathogen coevolution. This theory predicts that crossover hotspots will be associated with genes that play important roles in the immune system, where diversity may be beneficial. To investigate this idea we mapped patterns of crossover frequency in relation to genes that plants use to recognize pathogens. We observe that a subset of *Arabidopsis thaliana* resistance genes overlap with strong crossover hotspots. However, we also observe resistance gene coldspots, which correlate with chromosomal rearrangements between varietal lines (ecotypes). Using Arabidopsis ecotypes we also show that natural genetic variation has a strong modifying effect on resistance gene cluster recombination rates. Together our work shows complex variation in resistance gene crossover patterns, which may relate to heterogeneity in coevolutionary pressures experienced at different loci, in addition to the effects of structural genetic diversity.

## Introduction

During meiosis, homologous chromosomes recombine via reciprocal exchanges of DNA, called crossovers [1,2]. Crossovers initiate from DNA double strand breaks that are repaired using an interhomolog pathway [1–3]. Meiotic recombination frequency is highly variable along chromosomes and is concentrated in narrow (1–2 kb) hotspots in plants, animals and fungi [4–7]. Hotspot locations and activity are controlled by both genetic and epigenetic factors [4–7]. The “two-speed” genome hypothesis proposes that crossover hotspots will be differentially distributed among immunity versus housekeeping genes, as the former participate directly in host-pathogen coevolution, where recombination and diversity may be beneficial [8–12]. Here we investigate this hypothesis by experimentally mapping patterns of meiotic crossover recombination in relation to Arabidopsis NBS-LRR disease resistance genes.

The plant immune system consists of two major branches: (i) Pathogen Associated Molecular Pattern (PAMP) Triggered Immunity (PTI) and (ii) Effector Triggered Immunity (ETI) [13–15]. PTI signaling recognizes conserved PAMPs shared by many pathogenic microbes, including flagellin and chitin, via the action of receptor-like kinases [15]. ETI signaling involves recognition of specific pathogen effectors (the products of *Avirulence* genes, *Avr*) by matching plant disease resistance genes (*R* genes) [13,14]. *R* gene products typically share combinations of domains including TIR (Toll, Interleukin-1 and *R* genes), NBS (Nucleotide Binding Site) and LRR (Leucine Rich Repeats) [13,14,16,17]. R proteins can interact directly with pathogen Avirulence proteins (e.g. RRS1-R & Pop2 and L & AvrL567), or guard host targets and decoys (e.g. RPS2, RIN4 & AvrRpt2) [18–23]. Pathogen recognition by R proteins triggers hypersensitive cell death, in addition to systemic signaling that increases resistance in uninfected tissues [13,14]. *R* genes confer direct fitness benefits via resistance and tolerance to pathogens [24–26]. However, there is a cost of resistance for growth [26,27], and positive and negative epistatic effects on plant fitness have been observed between *R* gene loci. For example, *RPS4* and *RRS1* function together and are genetically linked [28–31], and a small number of *R* gene combinations can trigger autoimmune hybrid necrotic interactions [32–34].

*R* genes belong to large paralogous gene families, that show high levels of diversity within and between species [16,17,35,36]. For example, the *Arabidopsis thaliana* NBS-LRR gene family consists of ~190–200 members, of which 30 have characterized resistance functions (*R* genes) (S1 Table) [16,35]. In this study we consider 197 genes in the NBS-LRR gene family (S1 Table). NBS-LRR genes show evidence of high historical recombination rates, inferred from patterns of linkage disequilibrium (LD) between single nucleotide polymorphisms (SNPs) [37–40]. NBS-LRR genes also show the signatures of both directional and balancing selection [40–46], with the latter thought to reflect negative frequency-dependent interactions [47], heterozygote advantage (overdominance) [48], or selection in diffuse or fluctuating environments [26]. Physical clustering of NBS-LRR genes along chromosomes is common and extensive experimental evidence for recombination exists, including the signatures of non-allelic gene conversion and unequal crossover [16,49–59]. This includes demonstration that unequal crossing-over within the maize *Rp1* cluster can generate novel resistance specificities not present in either parent [54].

Relatively little is known about variation in fine-scale crossover frequency between *R* genes. We therefore sought to use experimental genetics to measure meiotic crossovers associated with the Arabidopsis NBS-LRR gene family. We observe strong crossover hotspots in a subset of NBS-LRR genes. However, we also observe significant NBS-LRR gene coldspots, which in some cases associate with structural heterozygosity. We mapped fine-scale crossover patterns within the *RESISTANCE TO ALBUGO CANDIDA1* (*RAC1*) *R* gene hotspot, which confers resistance against oomycete pathogens [60–62]. *RAC1* intragenic recombination was found to overlap exons encoding the TIR, NBS and LRR domains. Highest *RAC1* crossover frequency occurs adjacent to the promoter and overlaps hotspot-associated CTT-repeat DNA sequence motifs [37,63,64]. Using a crossover assay based on flow cytometry of fluorescent pollen, which allows >10,000s post-meiotic gametes to be scored per individual [65–67], we show significant modification of NBS-LRR cluster recombination rate by genetic background. Using Southern blots we present data on NBS-LRR gene structural variation between ecotypes and relate this to patterns of crossover frequency. Together these analyses reveal heterogeneity in *R* gene crossover frequency and show how natural variation modifies NBS-LRR cluster recombination.

## Results

### Mapping crossover frequency within the Arabidopsis NBS-LRR gene clusters

**Fig 1:**
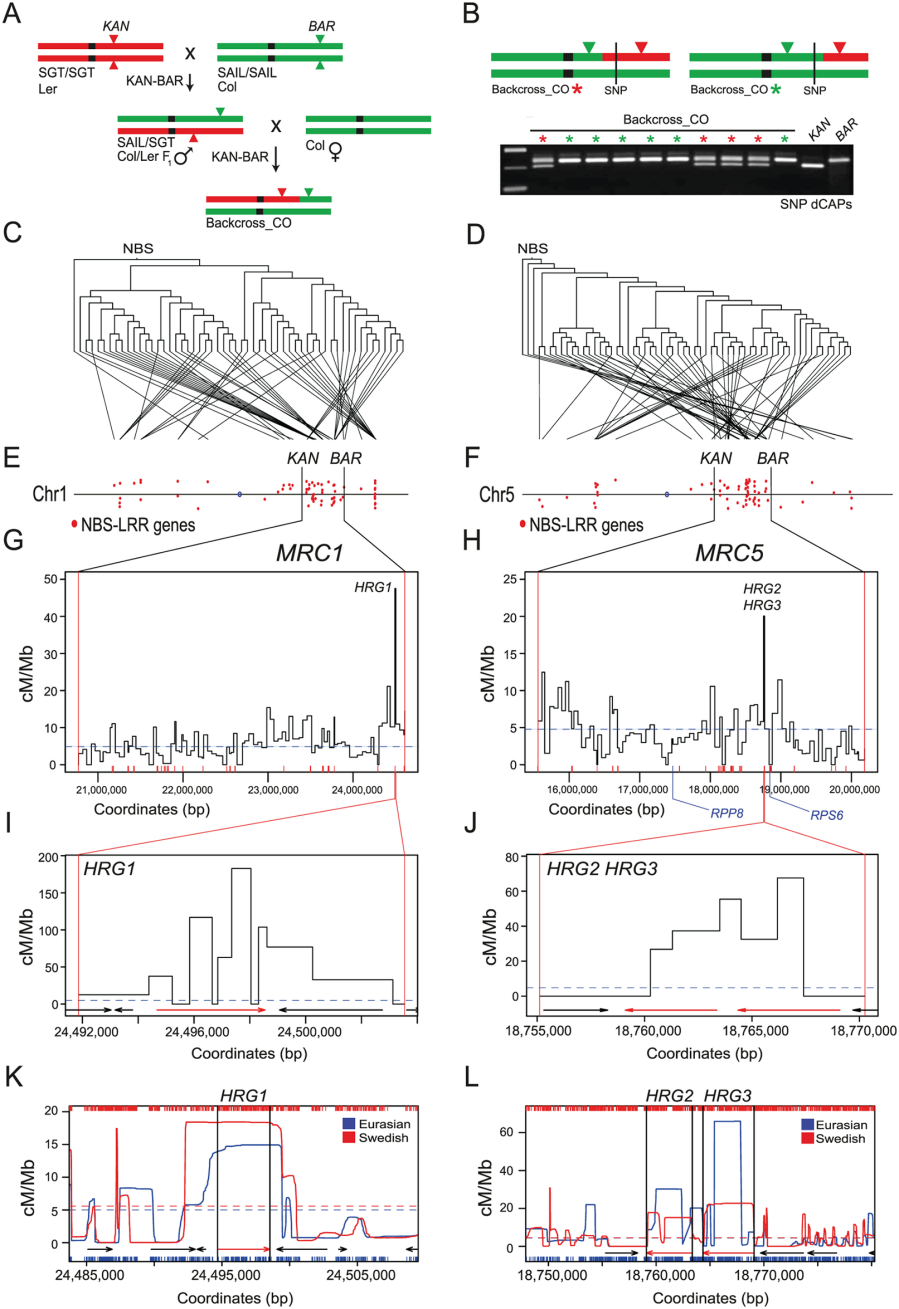
Meiotic recombination hotspots and coldspots within *MAJOR RESISTANCE CLUSTER1* and *MAJOR RESISTANCE CLUSTER5*. **(A)** Crossing scheme used to double-select *MRC* crossovers. *KAN* (SGT) Ler chromosomes are shown in red and *BAR* (SAIL) Col chromosomes are shown in green. Triangles represent insertions and are labeled with the resistance they confer (*KAN* = kanamycin, *BAR* = glufosinate). **(B)** Diagram illustrating *MRC* genotype expectations in double-resistant backcross progeny, depending on crossover location. A representative ethidium-stained genotyping gel for a Col/Ler single nucleotide polymorphism (SNP) dCAPs marker is shown beneath [70]. Colored stars above the genotyped samples match the chromosome diagrams above. **(C-D)** Phylogenetic trees generated using NBS domain amino acid sequences are plotted, with branch tips connected to the corresponding gene physical location. **(E-F)** Physical maps of chromosomes 1 and 5 with red dots indicating NBS-LRR gene positions (S1 Table). The selected *MRC* regions are indicated by black vertical lines and *KAN* and *BAR* symbols. **(G-H)** Recombination rate (cM/Mb) throughout the genotyped *MRC1* and *MRC5* regions (S2 and S3 Tables). Red x-axis ticks indicate NBS-LRR gene positions (S1 Table). The dotted horizontal line indicates the male Col/Ler chromosome average recombination rate [71]. The *RPP8* and *RPS6* coldspot locations are indicated beneath the *MRC5* map. **(I)** Crossover frequency within the *HRG1* hotspot interval (S4 Table). Arrows represent genes, with the *R* gene highlighted in red. **(J)** As for (I) but for *HRG2/HRG3* (S5 Table). **(K-L)** Historical recombination rates across *HRG1* and *HRG2/HRG3*. Crossover frequency (cM/Mb) estimates (LDhat) from 80 Eurasian and 180 Swedish accessions are shown in blue and red respectively [72–74]. X-axis ticks indicate SNP positions used for analysis. Horizontal dotted lines indicate chromosome mean historical recombination values. Arrows represent genes, with the NBS-LRR genes highlighted in red.

NBS-LRR genes frequently cluster in plant genomes [16]. For example, the majority of the Arabidopsis NBS-LRR domain gene family (114/197) are located in clusters on the south arms of chromosomes 1 and 5 (Fig 1 and S1 Table), which also correspond to regions of disrupted synteny between *A*.*thaliana* and *A*.*lyrata* chromosomes [16,36]. We constructed phylogenies using NBS domain amino acid sequences and compared the resulting trees to the physical location of NBS-LRR genes on chromosomes 1 and 5 (Fig 1C and 1D). This indicated that both tandem and dispersed NBS-LRR gene duplications contribute to cluster organization, consistent with previous analyses [16,36]. To map crossovers experimentally at high resolution within the clusters we designed a double-selection strategy (Fig 1A). This utilizes Syngenta Arabidopsis Insertion Library (SAIL) T-DNA insertions that confer glufosinate resistance in the Columbia (Col) accession, hereafter named *BAR* (Basta Resistant) lines [68], and Singapore Gene Trap (SGT) *Ds* insertions that confer kanamycin resistance in the Landsberg *erecta* (Ler) accession, hereafter named *KAN* (Kanamycin Resistant) lines (Fig 1A and 1B) [68,69]. Single copy *KAN* and *BAR* insertions that flank the NBS-LRR clusters on chromosomes 1 and 5 were identified using Southern blotting and genetic analysis (Figs 1 and S1). *KAN-BAR* insertion pairs used for crossover double-selection define the *MAJOR RESISTANCE CLUSTER1* (*MRC1*, 3.87 Mb, SGT_5517 and SAIL_148_D06) and *MAJOR RESISTANCE CLUSTER5* (*MRC5*, 4.69 Mb, SAIL_309_G06 and SGT_5183) map regions (Fig 1A and S2 and S3 Tables).

Paired *MRC KAN-BAR* insertions were crossed to generate double kanamycin-glufosinate resistant F_1_ *trans-*hemizygotes, which were then backcrossed as males to wild type Col females (Fig 1A). Backcross progeny were grown on kanamycin-glufosinate media and the ratio of double-resistant to sensitive individuals was used to measure genetic distances, according to the formula cM = 100×(2×(N_KB_*/*N_total_)) (Fig 1A and Table 1), where N_KB_ is the number of kanamycin-glufosinate double-resistant progeny and N_total_ is the total number of progeny screened. Genotyping of double-resistant individuals with Col/Ler Simple Sequence Length Polymorphisms (SSLP) markers was used to confirm that they had experienced a crossover within the selected *MRC* region (Fig 1B and S2 and S3 Tables). Average recombination rates within the *MRC* regions were comparable to the male Col/Ler genome average 4.82 cM/Mb (*MRC1* 5.89 cM/Mb, *MRC5* 4.18 cM/Mb) (Table 1 and S2 and S3 Tables) [71]. To analyze fine-scale recombination patterns within the *MRC* regions a subset of the double-selected *MRC1* (*n =* 725) and *MRC5* (*n =* 714) recombinant individuals were chosen for further analysis by internal genotyping (Fig 1 and S2 and S3 Tables). Double-resistant crossover progeny were genotyped for 93 and 90 Col/Ler SNPs within the clusters to identify *MRC1* and *MRC5* marker intervals containing crossovers, with a mean inter-marker distance of 46.5 kb (Fig 1 and S2 and S3 Tables). Analysis of crossover patterns in the *MRC* maps revealed intervals with both significantly higher and lower crossovers than expected if random (*MRC1*, *X*^*2*^ *=* 441.45 d.f. = 93 *P* = <2.2×10^−16^; *MRC5*, *X*^*2*^ *=* 337.29 d.f. = 90 *P* = <2.2×10^−16^), including intervals containing *R* genes (Fig 1 and S2 and S3 Tables).

**Table 1:** Double-selection of *MRC1* and *MRC5* crossovers. *KAN-BAR* F_1_ *trans*-hemizygotes were backcrossed to Col wild type female flowers and progeny were double-selected for kanamycin and glufosinate resistance. The numbers of double-resistant versus sensitive backcross seedlings are listed. The physical distances (megabase, Mb) were calculated between the *KAN* and *BAR* insertion sites relative to the TAIR10 Col reference assembly [75]. Genetic distance (cM) is calculated as 100×(2×(N_KB_*/*N_total_)), where N_KB_ is the number of Kan-Bar double-resistant backcross progeny and N_total_ is the total number of backcross progeny screened (Double-Resistant+Sensitive).

	*MRC1*	*MRC5*
Double-Resistant	1,028	947
Sensitive	7,988	8,716
Total	9,016	9,663
cM	22.80	19.60
Mb	3.87	4.69
cM/Mb	5.89	4.18

### NBS-LRR gene hotspots and coldspots within *MRC1* and *MRC5*

**Fig 2:**
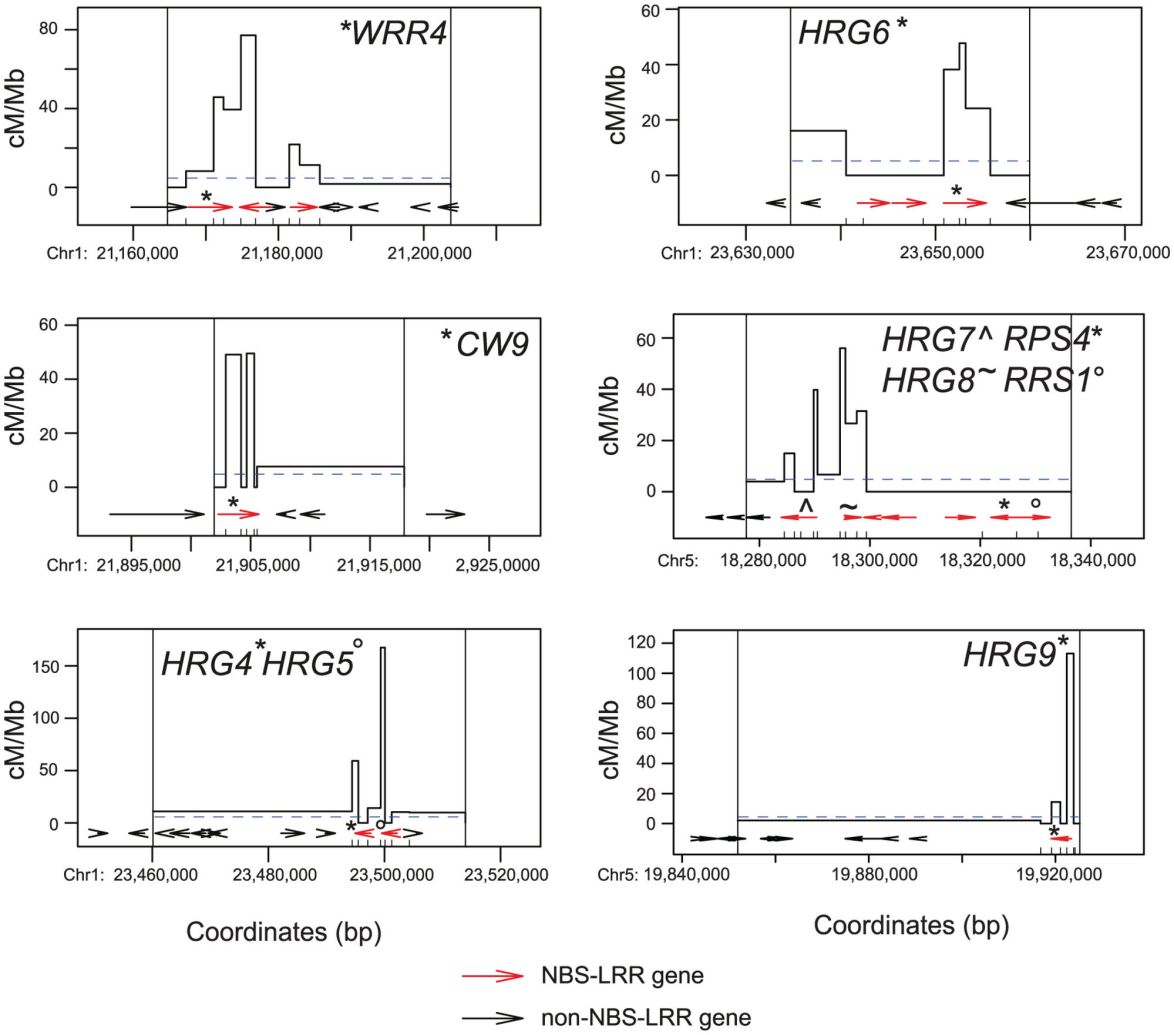
Fine-mapping *MRC* NBS-LRR gene crossover hotspots. Crossover frequency (cM/Mb) in high recombination *MRC* intervals containing NBS-LRR genes (S6–S11 Tables). Internal dCAPs marker positions are indicated by x-axis ticks, in addition to whether the region is located in *MRC1* (Chr1) or *MRC5* (Chr5). NBS-LRR genes are indicated by red arrows and non-NBS-LRR genes by black arrows. The chromosome average recombination rate for Col×Ler male backcrosses is indicated by horizontal, dotted blue lines [71]. Vertical black lines indicate the location of SNP markers genotyped in *MRC* maps (S2 and S3 Tables). NBS-LRR domain gene names are printed within the plot and symbols used to indicate which gene arrows they correspond to.

The *MRC1* interval with the highest recombination contains a functionally uncharacterized TIR-NBS-LRR gene (At1g65850), which for the purposes of this study we designate *HOT R GENE1* (*HRG1*) (Fig 1G and S2 Table). *HRG1* shares high sequence identity with *RPP1* and *DM2*, which confer resistance to *Hylaperonospora parasitica* and cause hybrid necrosis respectively [32,76]. The *HRG1* map interval contained 18 crossovers, which we fine-mapped by genotyping using derived Cleaved Amplified Polymorphisms (dCAPs) markers (Fig 1I) [70]. We observed a concentration of crossovers (14 of 18) within *HRG1*, with a peak recombination rate of 182.87 cM/Mb (Fig 1I and S4 Table), compared to the male Col/Ler genome average of 4.82 cM/Mb [71]. Similarly, the interval in *MRC5* with highest crossover frequency contains a tandem pair of TIR-NBS-LRR genes (At5g46260 and At5g46270), which we designate *HOT R GENE2* (*HRG2*) and *HOT R GENE3* (*HRG3*) (Fig 1H and S3 Table). *HRG2* and *HRG3* are also functionally uncharacterized, but show high sequence similarity with *RPS6* and *RAC1*, which confer resistance to *Pseudomonas syringae* and *Albugo candida* respectively [60,77]. Fine-mapping crossovers using additional dCAPs markers again revealed crossovers concentrated within the *HRG2* and *HRG3* NBS-LRR genes (11 of 11 crossovers) (Fig 1J and S5 Table). We fine-mapped crossovers in six additional high recombination intervals in *MRC1* and *MRC5* that contained NBS-LRR genes (Fig 2). We observed enrichment of crossovers within NBS-LRR genes in all cases (*WRR4*, *CW9*, *HRG4*, *HRG5*, *HRG6*, *HRG7*, *HRG8* and *HRG9*) (Fig 2 and S6–S11 Tables). Recombination rates observed at the *MRC* NBS-LRR hotspots were comparable to characterized plant hotspots and were detected at singleton, tandemly duplicated and complex NBS-LRR loci (Fig 2 and S4–S12 Tables).

To test for *MRC* intervals with significantly different crossover rates, we constructed 2×2 contingency tables and performed *X*^*2*^ tests using the observed crossover and non-recombinant counts, and those expected at random based on the physical size of each interval. Finally, we applied a false discovery correction, due to multiple testing [78]. Out of 185 intervals in *MRC1* and *MRC5*, 22 were significantly different from the null expectation (S2 and S3 Tables). Ten intervals showed higher recombination, which included *HRG1*, *HRG2*, *HRG3*, *HRG4* and *HRG5* (S2 and S3 Tables). Twelve intervals showed lower recombination, which included intervals containing *RRS1B-RPS4B*, the *RPS6* cluster and a cluster of 4 uncharacterized CC-NBS-LRR genes (At1g58807, At1g58848, At1g59124 and At1g59218) (S2 and S3 Tables). However, after correction for multiple testing, only *HRG1* and the CC-NBS-LRR cluster remained significantly different (S2 and S3 Tables) [78]. To test whether intervals containing NBS-LRR genes showed different recombination rates compared to those that did not, we compared observed crossover counts to those expected at random in proportion to physical distance, using a *X*^*2*^ test. *MRC* intervals that contained NBS-LRR genes did not have significantly different crossover counts compared to those that lacked NBS-LRR genes (*P* = 0.418).

As a further test of recombination in NBS-LRR genes compared to other genes, we analyzed 1,230 crossovers mapped throughout the genome by genotyping-by-sequencing of 192 Col×Ler F_2_ individuals (S13 Table) [79,80]. The crossovers were mapped to a mean resolution of 947 bp, representing 1,164,432 bp in total, which significantly overlapped with genes (Block-bootstrap Z = 4.54, *P* = 2.77×10^−6^). To compare gene groups we sampled from the non-NBS-LRR genes using parameters that approximated the clustering structure of the NBS-LRR genes (S2A and S2B Fig). We repeatedly sampled and tested for overlap between samplings and crossovers, requiring at least one base pair in common to score an overlap. Values were also length-normalized by rescaling the observed data by the total length in base pairs of the sample or gene set (S3C–S3F Fig). For both NBS-LRR and non-NBS-LRR genes the observed proportion of genes overlapping a crossover lie within those observed for the sampled data, usually near the distribution’s center (S3C–S3F Fig). Therefore we conclude that the number of NBS-LRR and non-NBS-LRR genes overlapping crossovers is not substantially different, and that both groups show the presence of hotspots and coldspots.

If recombination hotspots within NBS-LRR genes have persisted in the species, then decay of linkage disequilibrium (LD) between natural genetic polymorphisms is expected [81,82]. For example, the presence of all four possible gametes for pairs of linked polymorphisms (i.e. AB, aB, Ab and ab) can be used as a measure of historical recombination [81,82]. We therefore analyzed single nucleotide polymorphisms (SNPs) from 80 Eurasian and 180 Swedish Arabidopsis accessions using LDhat [72–74]. Consistent with our experimental crossover measurements *HRG1*, *HRG2* and *HRG3* show elevated historical recombination (Fig 1K and 1L) [72–74]. We also observed intervals containing *R* genes, like *RPP8*, with low experimental recombination, but which exhibited high historical recombination frequency (Fig 1H and S1–S3 Tables). The suppressive effect of structural polymorphism on recombination is likely to contribute to these patterns. For example, *RPP8* is tandemly duplicated in Ler relative to Col [50], and the *RPS6* cluster shows complex Col/Ler structural polymorphism [77]. In summary, although overall *MRC* recombination rates were close to the genome average, we observed heterogeneity in local crossover frequency and the presence of NBS-LRR gene associated hotspots and coldspots.

### Intragenic recombination hotspots in the *RAC1* resistance gene

**Fig 3:**
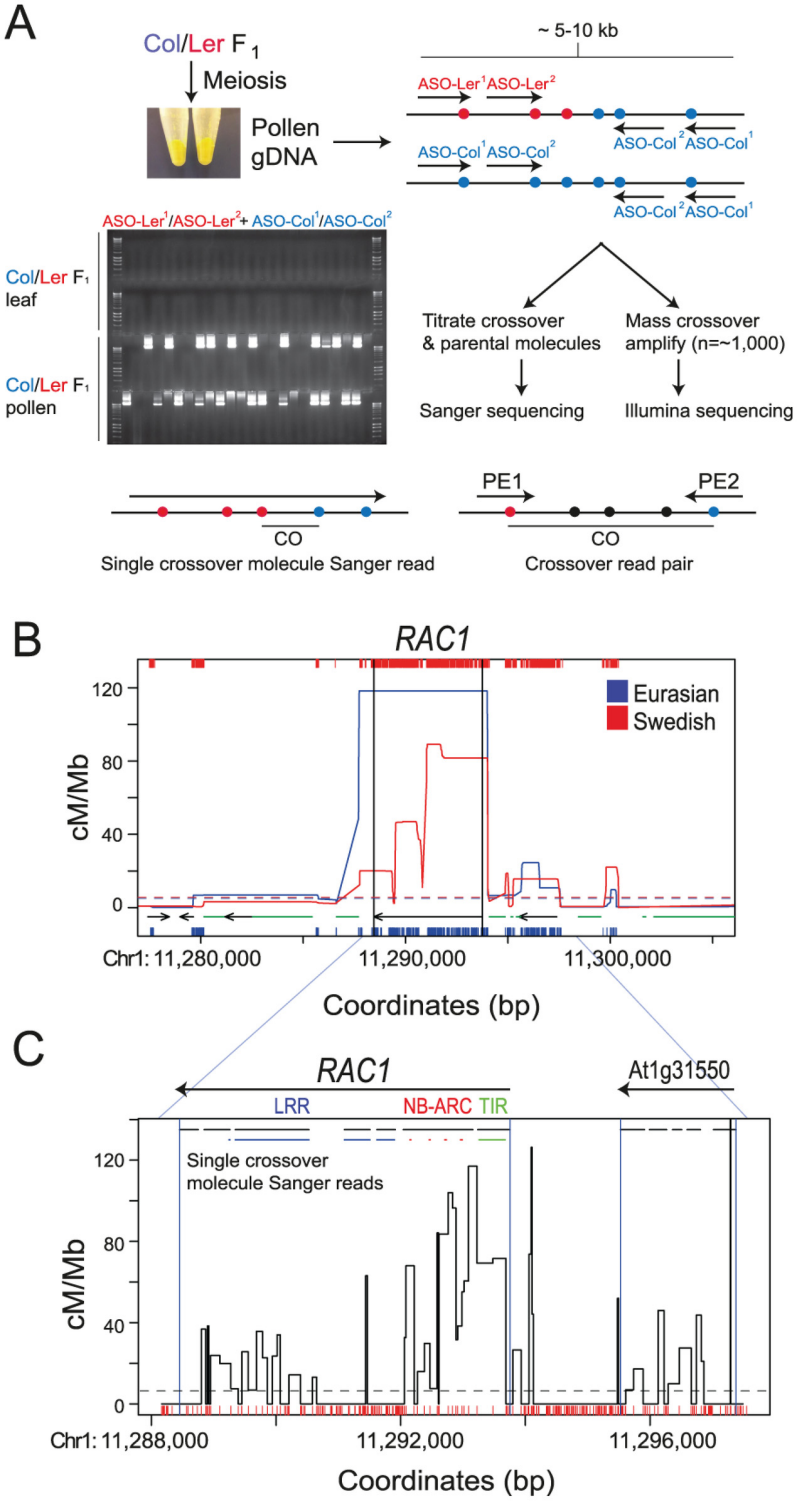
Intragenic crossover hotspots within the *RAC1* resistance gene. **(A)** Diagram illustrating pollen-typing methods used to fine-map plant crossover hotspots. Pollen is collected from Col/Ler F_1_ plants and genomic DNA is used for two rounds of nested allele-specific PCR amplifications with either crossover (ASO-Ler+ASO-Col) or parental (ASO-Col+ASO-Col) allele-specific oligonucleotide (ASO) primer combinations. An ethidium-bromide stained agarose gel is shown with crossover-specific amplifications from Col/Ler F_1_ pollen, which are not observed when using Col/Ler F_1_ leaf (somatic) DNA as a template. Crossover positions are identified using either Sanger sequencing from single crossover molecule amplifications, or paired-end sequencing of crossover molecule libraries. **(B)** Historical crossover frequency over *RAC1* estimated by LDhat analysis of polymorphisms from 80 Eurasian (blue) and 180 Swedish (red) accessions [72,73]. X-axis ticks indicate SNP positions used for analysis. Horizontal dotted lines indicate chromosome mean values. Black arrows represent genes and green horizontal lines indicate transposable elements. Black vertical lines indicate *RAC1* TSS and TTS positions. **(C)** Crossover frequency within the *RAC1* amplicon measured by crossover and parental molecule titration, followed by single crossover molecule Sanger sequencing. The positions of *RAC1* and the adjacent gene (At1g31550) are indicated by horizontal black arrows above the plot. Exons are indicated by horizontal black lines, with sequences encoding RAC1 TIR (green), NB-ARC (red) and LRR (blue) domains highlighted as coloured lines. For the NB-ARC domain, the position of the Walker A, Walker B, ARC1 and ARC2 motifs are shown. Red x-axis ticks show Col/Ler SNP positions. Blue vertical lines indicate the positions of gene TSS and TTS from TAIR10 representative gene models.

To investigate crossover variation within a single *R* gene we used pollen-typing [37,83,84], which is analogous to mammalian sperm-typing approaches [85,86]. This technique uses two rounds of nested, allele-specific PCR to specifically amplify and titrate either crossover or parental (non-recombinant) molecules from F_1_ (e.g. Col/Ler) pollen DNA (Fig 3A and S3 Fig). Importantly, crossover products are not amplified from Col/Ler F_1_ somatic leaf DNA under the same PCR conditions (Fig 3A). This demonstrates that template switching is not generating crossover molecules during PCR amplification. The concentrations of parental and crossover molecules, measured by titration, are used to calculate recombination rate within the amplified region [37,83–85]. Amplification from single molecules containing crossovers, followed by Sanger sequencing, is used to identify internal recombination locations, to the resolution of individual polymorphisms (Fig 3A) [37,83,84]. Between 100–200 crossover molecules are Sanger sequenced to map the topology of recombination within an amplified region and calculate crossover frequency (cM/Mb) for each marker interval (Fig 3A) [37,80,83,84]. Physical distances analyzed by pollen-typing are typically between 5–10 kb [37,80,83,84].

To select candidate hotspot *R* genes for pollen-typing analysis we analyzed historical recombination rates [37,72–74]. We used LDhat to analyse SNPs from 80 Eurasian and 180 Swedish Arabidopsis accessions [72,73], and observed that *RPP8* and *RAC1* are the *R* genes with highest LD-based recombination estimates (S1 Table). However, the *RPP8* locus shows structural polymorphism between accessions, including between Col and Ler, making it unsuitable for pollen-typing analysis [50]. In contrast, *RAC1* is present as a TIR-NBS-LRR singleton in both Col and Ler and so was selected for further study. *RAC1* alleles confer resistance to *Hyaloperonospora arabidopsidis* and *Albugo candida* and shows high sequence identity with *HRG2*, *HRG3* and *RPS6* [60–62]. *RAC1* resistance has been mapped using the Acem1 Albugo isolate, which both Col and Ler are susceptible to [60–62]. We amplified a 9.45 kb region, spanning *RAC1* and an adjacent gene (At1g31550) that encodes a putative lipase, and measured a recombination rate of 16.99 cM/Mb, which is comparable to known plant crossover hotspots (Fig 3C and S12 and S14 Tables). After sequencing 181 single molecules containing crossovers we observed highest recombination within the *RAC1* open reading frame, with a peak intragenic crossover frequency of 117 cM/Mb (Fig 3C and S14 and S15 Tables). *RAC1* recombination was highest in the 5' exons that encode the TIR and NBS domains, and in a 3'-region encoding LRR domains (Fig 3C). This pattern is consistent with high intragenic recombination rates observed at the 5' and 3' ends of plant genes [37,63,64,87]. In this regard it is important to note that residues critical for resistance function have been mapped to all three domains (TIR, NBS and LRR) in other *R* genes [58,88–91]. The open reading frame of the adjacent gene At1g31550 also showed substantial crossovers (Fig 3C). Together these data demonstrate that *RAC1* contains strong historical and contemporary crossover hotspots.

**Fig 4:**
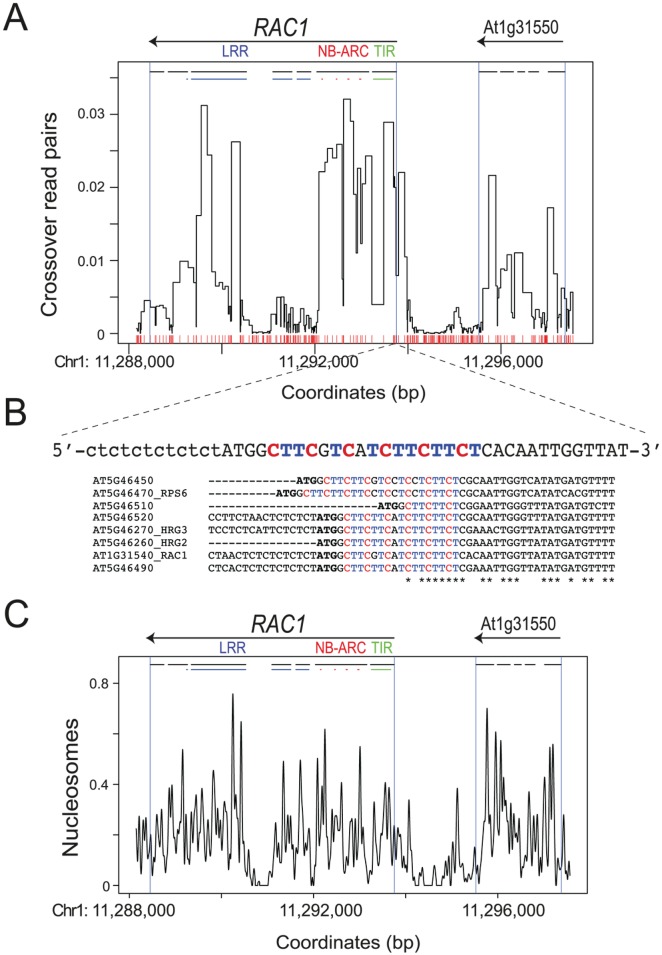
*RAC1* crossovers measured by high-throughput sequencing, nucleosome occupancy and CTT-repeat motifs. **(A)** Normalized crossover read pair values are plotted relative to the TAIR10 reference sequence, with the position of Col/Ler polymorphisms indicated by red x-axis ticks. Labelling is as for Fig 3C. **(B)** The sequence of the *RAC1* CTT-repeat motif is printed and connected to its physical location in (A) with dashed lines. Note the sequence is the reverse complement relative to the orientation of plotting in (A). The presence of CTT-motifs in *RAC1*-related paralogs in the Col-0 accession are shown in a multiple sequence alignment, where gene start codons (ATG) are in bold type. The asterisks beneath the alignment indicate identical positions. **(C)** Normalized nucleosome occupancy values (MNase-seq) plotted as for (A).

To analyze greater number of recombination events, we mass-amplified approximately 1,000 *RAC1* crossover molecules using 672 independent PCR reactions (Fig 3A). Each reaction was estimated to contain between 1 and 2 crossover templates, based on our previous titration experiments (S14 Table). The resulting crossover amplification products were then pooled, sonicated and used to generate sequencing libraries (Fig 3A). Paired-end sequencing was performed and read pairs were separated and aligned to the Col and Ler parental *RAC1* templates, allowing only exact matches (S16 Table). Reads were filtered for those that aligned to one parent only, and for pairs with a centromere-proximal read mapping to Col and a centromere-distal read mapping to Ler, on opposite strands (S16 Table). Read pairs matching these criteria are consistent with the crossover allele-specific oligonucleotide (ASO) configuration used during PCR amplification (Fig 3A). These filters yielded 182,909 total crossover read pairs (S16 Table). We assigned recombination values to marker intervals between crossover read pairs, weighted by the number of intervals and their widths, and normalized by the total number of mapping reads (Fig 4A). This revealed a similar recombination pattern to that observed from single molecule Sanger sequencing, with high recombination overlapping the *RAC1* TIR, NB-ARC and LRR domain exons (Figs 3C and 4A). Crossover read pair analysis also confirmed substantial recombination activity within the adjacent gene At1g31550 (Fig 4A). Together these data demonstrate focused intragenic recombination in *RAC1* overlapping exon sequences [60–62].

We investigated the extent to which chromatin and DNA sequences associate with crossover frequency at the *RAC1* hotspot. Previous studies have identified degenerate CTT and CCN repeat motifs associated with Arabidopsis hotspots, enriched at the 5′-end of genes [37,63,64]. We observed a CTT-motif (CTTCGTCATCTTCTTCT) almost immediately downstream of the *RAC1* start codon, within the TIR-domain encoding exon that shows high recombination rate (Fig 4A and 4B). CTT-motifs are also observed in *RAC1-*related NBS-LRR genes (Fig 4B). To investigate association of CTT-motifs with recombination we divided NBS-LRR genes located within the *MRC* maps into those in intervals with higher or lower crossover frequency, compared to the male Col×Ler genome average (4.82 cM/Mb) [71]. 62% (21 of 34) of high recombination NBS-LRR genes showed at least one match to a 16-mer CTT-repeat motif that we previously identified as enriched at historical crossover hotspots (S17 Table) [37]. In contrast, significantly fewer (33%, 16 of 49) low recombination NBS-LRR genes showed matches to this CTT-motif (S17 Table) (*X*^*2*^ *P* = 0.016). This analysis further demonstrates association between high crossover recombination and CTT-repeat motifs in Arabidopsis [37,39,63,64]. To analyse chromatin structure we performed micrococcal nuclease digestion of wild type (Col-0) chromatin and gel purified the resulting ~150 bp mononucleosomal DNA band [92]. This DNA was used to generate a library and paired-end sequencing performed, followed by analysis using the nucleR package [93]. We observed that intergenic regions within the pollen typing amplicon, and *RAC1* intron 4, showed relatively low nucleosome occupancy (Fig 4C) [92]. Highest crossover rates correlated with nucleosome-occupied exon sequences within *RAC1* and At1g31550 (Fig 4A and 4C). Together these analyses demonstrate associations between *RAC1* crossover frequency, DNA sequence motifs and chromatin structure.

### *R* gene structural variation and crossover modification by genetic background

**Fig 5:**
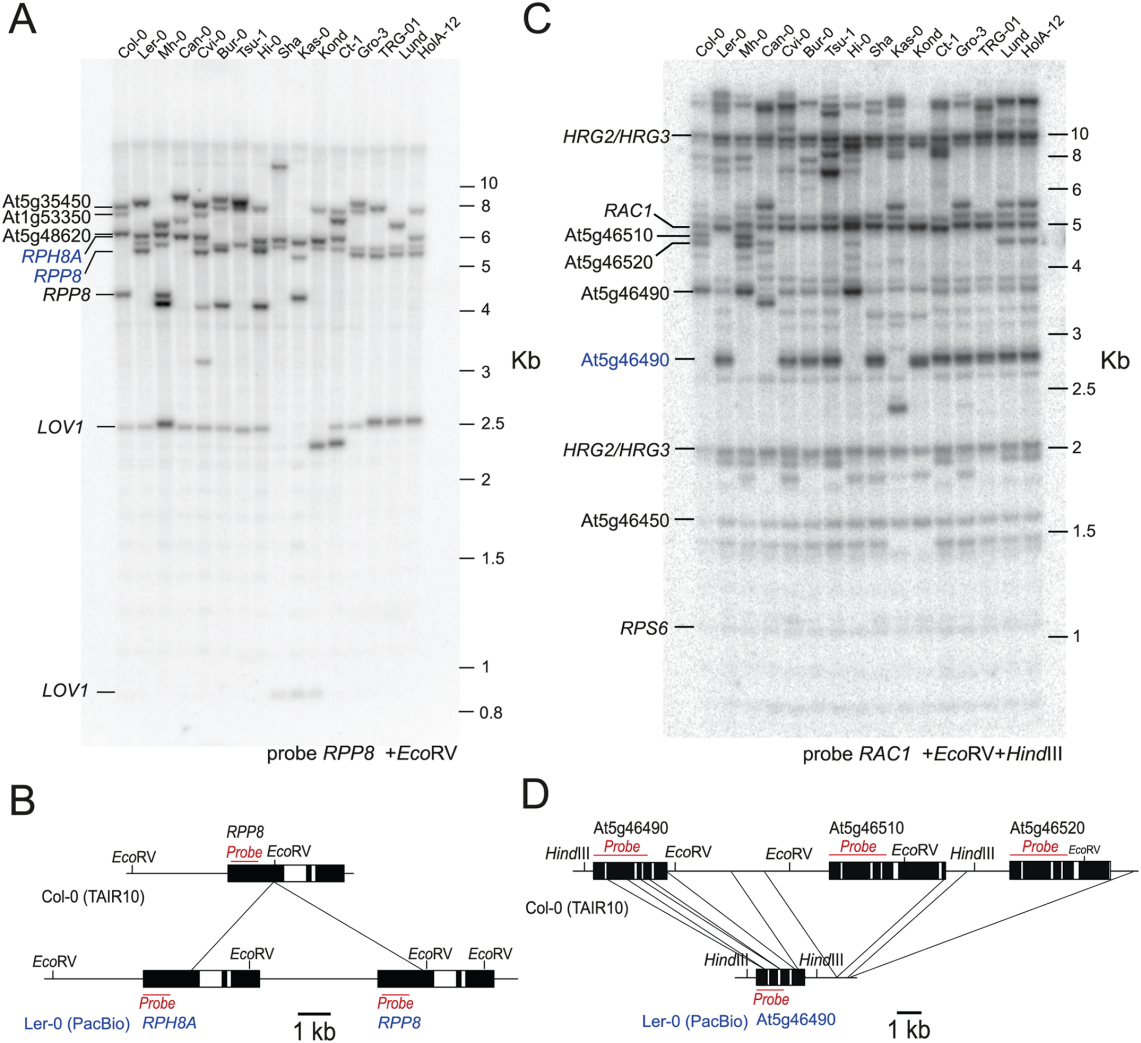
NBS-LRR gene structural diversity between Arabidopsis accessions. **(A)** Southern blotting and hybridization analysis of *RPP8* in the indicated accessions. Cross-hybridizing NBS-LRR genes in Col-0 predicted from the TAIR10 reference assembly are labelled in black. In Col-0 *RPP8* is detected as a 4.3 kb *Eco*RV band, and two 5.5 and 6.1 kb bands in Ler-0. Bands corresponding to Ler-0 *RPP8* and *RPPH8A* genes are labelled in blue. **(B)** Diagram showing annotated structural variation at *RPP8* between Col-0 (TAIR10) and Ler-0 (PacBio) assemblies [94]. Regions predicted to cross-hybridize with the probe are indicated in red, in addition to the position of *Eco*RV sites. **(C)** and **(D)** are as for (A) and (B), but probing for *RAC1*. The *RAC1* probe cross-hybridizes with a linked cluster of NBS-LRR genes, including *RPS6*. In Col-0 the *RPS6* cluster includes three NBS-LRR genes (At5g46490, At5g46510, At5g46520) that are present as a single gene (At5g46490) in Ler-0; such that in Col-0 three bands of 3.7, 4.5 and 4.7 kb are detected after *Eco*RV and *Hind*III digestion, and one 2.8 kb band in Ler-0. The Ler-0 band is labelled in blue.

The *HRG1*, *HRG2*-*HRG3* and *RAC1* NBS-LRR hotspots show historical and experimental recombination rates above the genome average (Figs 1, 3, S4, S5, S14 and S15 Tables), whereas other *R* genes differed between these two measures. For example, *RPP8* shows high historical recombination, yet low experimental crossover frequency (Fig 1H and S3 Table). *RPP8* is a singleton *R* gene in the Col reference genome, but in Ler occurs as a tandemly duplicated pair with *RPH8A* [50]. Sequence analysis indicates that the Col *RPP8* haplotype is derived from a gene fusion event between *RPP8* and *RPH8A* [50]. We performed Southern blotting and hybridization analysis and confirmed the *RPP8/RPH8A* Col/Ler structural polymorphism (Fig 5A) [50]. The observed band sizes are also consistent with a recent Ler genome assembly generated by PacificBiosystems (Fig 5B) [94]. Hence, low *RPP8* recombination in the Col/Ler *MRC5* map may be explained due to structural heterozygosity inhibiting crossovers [95,96]. Similarly, the *RPS6* cluster shows structural polymorphism between Col and Ler and an absence of crossovers in *MRC5*, despite showing high sequence similarity to the hotspot *R* genes *RAC1*, *HRG2* and *HRG3* (Figs 1H, 5C and 5D and S3 Table) [77]. In contrast the hotspot *R* genes *HRG1*, *HRG2*, *HRG3* and *RAC1* show low levels of structural polymorphism between accessions, including between Col and Ler (Figs 5C and 5D and S4). For the *R* genes *RPP1* and *RPP4* Southern blotting and hybridization analysis revealed complex patterns of structural polymorphism that did not obviously correspond to the PacBio Ler assembly (S5 and S6 Figs) [94]. Hence, for these *R* genes structural polymorphism is also likely to be inhibitory to recombination, when divergent haplotypes are heterozygous.

**Fig 6:**
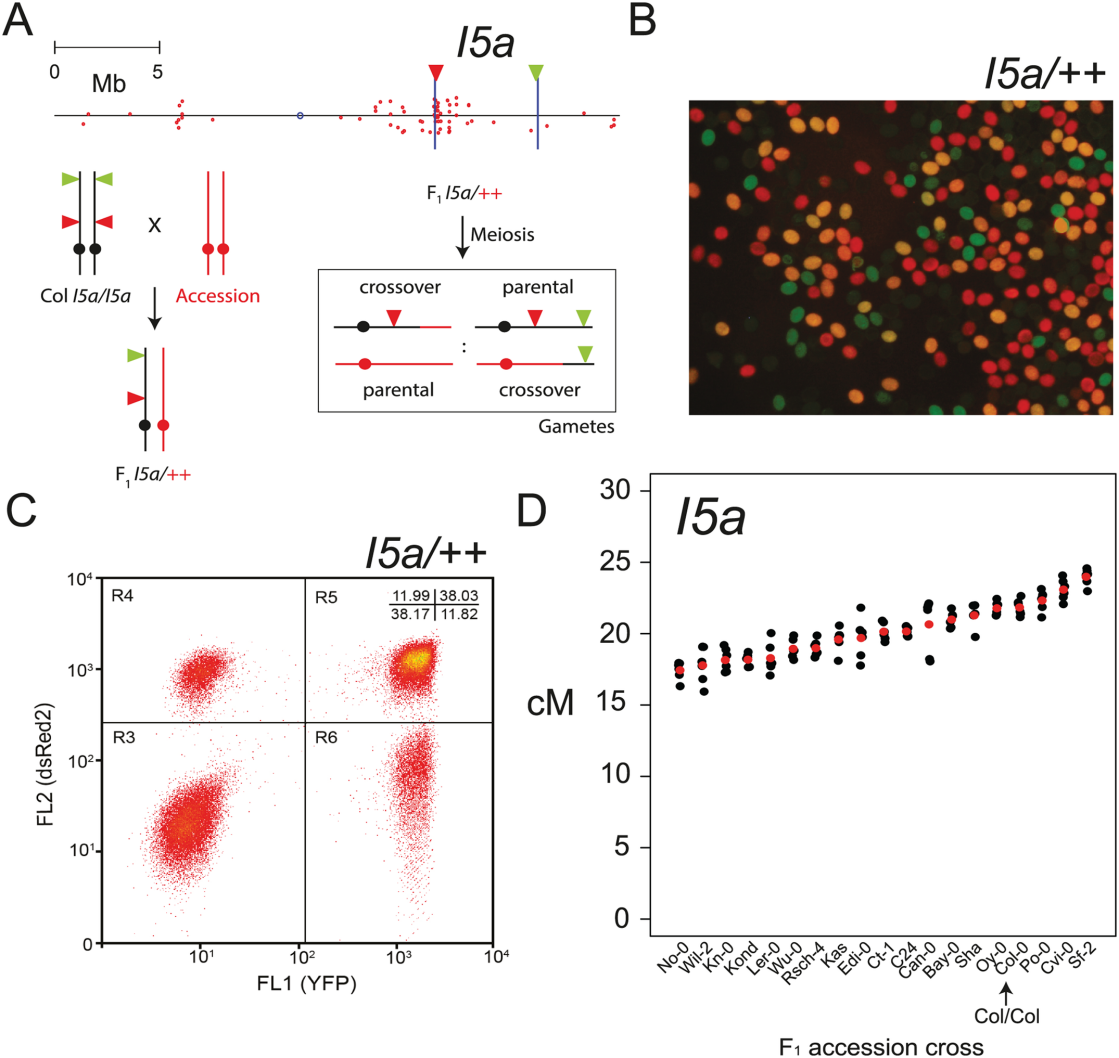
NBS-LRR cluster recombination rate is modified by natural genetic variation. **(A)** Physical map of chromosome 5 with NBS-LRR gene positions indicated by red dots. The positions of FTL T-DNAs that define *I5a* are indicated by vertical blue lines, with triangles indicating the colour (red or yellow) of fluorescent protein encoded. Beneath is a diagram illustrating the generation of FTL hemizygous F_1_ plants and scoring of crossover recombination in pollen. Crossover within *I5a* leads to pollen with red or yellow fluorescence alone, whereas the parental classes are either without fluorescence or show both red and yellow. **(B)** Fluorescence micrograph of pollen from *I5a/++* hemizygotes, showing the presence of four fluorescent classes. **(C)** A representative flow cytometry plot of pollen from *I5a/++* hemizygotes. Points represent individual pollen grains measured for yellow (FL1 YFP) and red (FL2 dsRed2) fluorescence. **(D)** *I5a* genetic distance (cM) measured from individual F_1_ plants (S18 Table). Mean values are shown in red. The accession crossed to is indicated on the x-axis. The Col/Col homozygous sample is labelled and indicated with an arrow.

To investigate the extent of inter-cross variation in NBS-LRR cluster recombination we used segregation of linked Fluorescent Tagged Line (FTL) T-DNA insertions [67,97]. Inheritance of FTLs in pollen can be monitored via fluorescence microscopy or flow cytometry (Fig 6A) [67,97]. Pollen grains collected from FTL/++ hemizygotes consists of four fluorescent classes that represent parental (no color and red+yellow) versus crossover recombinant (red or yellow alone) chromosomes (Fig 6B). The ratios between these fluorescent pollen classes are used to calculate genetic distance between the FTL T-DNA insertions (Fig 6A and 6B) [66,67,97]. Pollen fluorescence was analyzed using a flow cytometer allowing >10,000s of pollen grains to be scored per individual plant (Fig 6C and S18 Table) [67]. We used FTL lines that define a 4.92 Mb interval called *I5a*, which has a 2.09 Mb overlap with the *MRC5* region and contains 33 out of 70 chromosome 5 NBS-LRR genes (Fig 6A and S1 Table). We crossed *I5a* (generated in the Col-0 background) to 18 Arabidopsis accessions representing global diversity and measured genetic distance in replicate F_1_ plants, scoring ~30,000 pollen grains per individual (Fig 6 and S18 Table). We observed 3 F_1_ crosses with significantly higher *I5a* recombination and 15 F_1_ crosses with significantly lower *I5a* recombination, relative to Col/Col homozygotes (general linearized model *P*<2.0×10^−16^) (Fig 6D and S18 Table). This demonstrates the sensitivity of NBS-LRR cluster recombination rates to genetic background, which is consistent with observed *cis* and *trans* modification of crossover frequency by natural genetic variation in plants [97–105]. Therefore, Arabidopsis genetic variation has a significant modifying effect on crossover frequency within the NBS-LRR clusters.

## Discussion

The ‘two-speed’ genome hypothesis predicts recombination hotspots associated with disease resistance genes, due to the beneficial effects of diversity during pathogen coevolution [8–11]. Consistent with this we observed a subset of strong *R* gene hotspots. For example, *RAC1* shows high historical and contemporary crossover frequency, comparable to known plant hotspots (S12 Table). However, not all NBS-LRR genes are hotspots and a substantial number of coldspots were observed. Coldspot *R* genes in the *MRC* maps correlated with structural heterozygosity in some cases, for example, *RPS6* and *RPP8*. Heterogeneity between NBS-LRR genes is well documented in (i) patterns of sequence diversity and evolutionary history [44,107], (ii) the degree of sequence exchange observed between paralogs [57,59,106,107], (iii) direct versus indirect modes of effector recognition [13,20] and (iv) the extent of gene clustering [108]. Here we additionally demonstrate heterogeneity in Arabidopsis NBS-LRR gene meiotic recombination rates. The ecology, generation time and effector complement of a given pathogen will interact to determine the selection pressures on NBS-LRR genes. It is also important to consider that domains required for recognition versus downstream resistance signaling within a single NBS-LRR gene may be under distinct forms of selection, e.g. diversifying versus purifying. Hence, for a given *R-Avr* interaction, higher or lower recombination rates, at varying scales, may be beneficial. Relevant to these considerations is the degree of outcrossing in Arabidopsis, which is estimated between 1–3% [39,109]. For example, genotyping of local stands of Arabidopsis in Germany detected clear evidence for recent outcrossing and heterozygosity [110]. The rapid decay of LD in Arabidopsis also indicates significant levels of historical outcrossing [39,72,73,111].

We observed pronounced intragenic variation in *RAC1* crossover frequency, with TIR, NB-ARC and LRR encoding exons showing high recombination. It is important to note that all three domains have been shown to control resistance specificity and sensitivity in other *R* genes [88–91]. We propose that intragenic recombination patterns can be selected through a combination of sequence and epigenetic determinants. For example, CTT and CCN repeat sequence motifs associate with high recombination in Arabidopsis [37,63,64]. Similarly, we observed that CTT-motifs were enriched in high recombination NBS-LRR genes located in the *MRC* maps. These motifs co-localize with the +1 H2A.Z histone variant-containing nucleosome and also significantly overlap with historical and experimental recombination [37,63,64]. CTT/CCN motifs bear similarity to C-rich motifs bound by mammalian PRDM9 proteins, which determine hotspot locations via histone H3K4^me3^ [112–117]. However, no clear homolog of PRDM9 exists in plants, and so CTT/CCN repeat motifs likely function via a different mechanism [7]. Association of high recombination and AT-rich sequences upstream of transcriptional start sites is also observed in Arabidopsis, which correlates with regions of low nucleosome occupancy [37,39,63,64]. Hence, Arabidopsis hotspot-associated DNA sequence motifs may function directly to recruit recombination-promoting factors, or alter chromatin and thereby influence accessibility of recombination factors. Extensive evidence exists for *cis* and *trans* acting genetic modifiers of meiotic recombination in plants, which also have the potential to influence *R* gene crossovers [97–105]. The location of *R* genes along the chromosome is also likely to be a significant factor, as recombination rate shows broad scale variation, in addition to sex-specific patterns in the sub-telomeric regions [37,71,103,118].

Varying recombination rates within and between *R* genes has important consequences for the generation of recognition diversity, as gene conversion and unequal crossover have been associated with changes to *R* gene structure and function [16,49–59]. Heritable gene conversion events occur during meiosis and are associated with both crossover and non-crossover DNA double strand break repair [119,120]. Extensive NBS-LRR sequence analysis indicates gene conversion events within and between *R* paralogs, which are associated with *Avr* recognition differences. For example, in the tomato *Cf-4/9* and *Cf-2/5* clusters [49,121], the rice *Xa21* cluster [122], the lettuce *Dm3* cluster [51,123], the Arabidopsis *RPP8* and *RPP5* genes [50,52,53,106] and the Flax *M* and *N* loci [59,124]. Hence, higher recombination rates are likely to promote generation of functional allelic diversity via associated gene conversion events. Unequal crossovers contribute to changes in *R* gene resistance function by fusing, duplicating or deleting genes, or clusters of genes [49,51,53,54,56,59,125]. This is believed to occur due to misalignment of related, but non-allelic, sequences during strand invasion, followed by crossover recombination. Unequal recombination has been directly observed between the tomato *Cf4* and *Cf9* genes, which encode membrane bound LRR-repeat proteins that control resistance to *Cladosporium fulvum* [49,121,126]. Interestingly, increased levels of unequal recombination were detected in *Cf-4/Cf-9* heterozygotes compared with *Cf-9/Cf-9* homozygotes, suggesting that polymorphism can promote this type of recombination [49]. Similar testcrosses were performed with the maize *Rp1* cluster, which confers resistance against *Puccinia sorghi*, and significant levels of crossover-associated resistance change were observed using both heterozygotes (*Rp1-J1/Rp1-J3*) and homozygotes (*Rp1G/Rp1G*) [54–56,127]. Finally, the lettuce *Dm3* cluster that confers resistance against *Bremia lactucae* was found to show low recombination rates, though deletions associated with crossovers were observed [51].

Besides unequal recombination, canonical crossovers within *R* genes have the potential to generate new resistance functions, as amino acids controlling recognition specificity or signaling are known to occur in TIR, NBS and LRR protein domains [13,58,88–91,128]. As *R* genes tend to cluster, crossovers within these clusters can also generate or break beneficial resistance haplotypes, for example when linked *R* gene alleles function together [28–31]. High recombination rates are predicted to lead to high levels of structural diversity. However, we demonstrate that the *RAC1* resistance gene shows high recombination, yet occurs as a singleton TIR-NBS-LRR gene in all Arabidopsis ecotypes tested. In flax the *L* resistance gene is similar to *RAC1* in showing stability as a singleton TIR-NBS-LRR gene in all backgrounds, but with a high diversity of resistance alleles [57]. *RAC1* and *L* are both closely related to complex, tandemly duplicated loci that include *RPS6* and *M* respectively [57,59,60,77]. Hence, it will be interesting to further define how contrasting patterns of sequence diversity are maintained at related *R* gene loci and how this relates to meiotic recombination.

*R* gene clusters are reminiscent of mammalian major histocompatibility complex (*MHC*) regions [129]. For example, the 3–4 Mb *MHC* region on human chromosome 6 contains 240 genes, including multiple human leukocyte antigen (*HLA*) genes [129]. Both *MHC* and *R* cluster regions show high sequence diversity, the signatures of balancing selection and high non-synonymous:synonymous substitution ratios [38,40,42,44–46,129,130]. Although human genetic maps generally show low recombination in *MHC* regions, punctate hotspots have been observed (e.g. *DNA3*, 140 cM/Mb) [131]. Interestingly, virulence effectors have also been observed to cluster in the genomes of eukaryotic pathogens [132]. For example, the genome of the oomycete *Phytophora infestans* (potato blight) shows conserved gene-dense regions, interspersed with diverse repetitive blocks where virulence effectors are located [9,132], and the basidiomycete fungus *Ustilago maydis* genome contains clustered genes encoding secreted virulence proteins that are transcriptionally induced during infection [11]. Similarly, the human pathogens *Trypanosoma brucei* and *Plasmodium falciparum* show sub-telomeric clustering of variant surface glycoproteins [133,134]. We speculate that coevolutionary dynamics drive mirrored changes in host and pathogen genomes and clustering of selected genes. It will be important to further explore inter-relationships between recombination, selection and plant-pathogen ecologies and how they influence diversity and gene organization at host resistance and pathogen effector loci.

## Materials and Methods

### NBS-LRR gene identification and analysis

We combined published annotations of the Arabidopsis NBS-LRR family with our own manual searches and selected a set of 197 genes for analysis (S1 Table) [16,35]. To analyse phylogenetic relationships NBS domain amino acid sequences were aligned using ClustalOmega and gaps treated as missing data. The resulting alignment was analysed using the PhyML 3.0 webserver (http://www.atgc-montpellier.fr/phyml/) with 500 bootstraps performed for tree construction [135]. To search for CTT-repeat motifs associated with NBS-LRR genes, a position weight matrix from a historical hotspot-enriched motif was matched at 80% to 1 kb windows surrounding NBS-LRR gene transcriptional start sites (TSSs).

### Double-selection and analysis of *MRC1* and *MRC5* crossovers

To select for crossovers with the NBS-LRR gene clusters we followed an identical strategy to that used previously for the *TEL1* interval (Fig 1A) [37]. SAIL T-DNA insertions conferring glufosinate resistance in the Col accession (hereafter named *BAR*), and *Ds* insertions conferring kanamycin resistance in the Ler accession (hereafter named *KAN*), were chosen with insertion sites flanking the NBS-LRR gene clusters on the south arms of chromosomes 1 and 5 [68,69]. Southern blotting and hybridization was used to identify single copy *KAN* and *BAR* insertion lines (S1 Fig). Single copy lines were tested that (i) self-fertilization of homozygotes gave ~100% resistant progeny, (ii) self-fertilization of hemizygotes gave ~75% resistant progeny and (iii) backcrossing hemizygotes to wild type gave ~50% resistant progeny (S1 Fig). After screening we selected SGT_5517 (*KAN*) and SAIL_148_D06 (*BAR*) for chromosome 1 and SAIL_309_G06 (*BAR*) At5g49220 and SGT_5183 (*KAN*) for chromosome 5. *KAN* and *BAR* lines were then crossed to generate *trans*-hemizygote F_1_ individuals, which were backcrossed as males to Col-0 (Table 1). Backcross progeny were germinated on media containing kanamycin and glufosinate to select for individuals that experienced a crossover between the *KAN* and *BAR* insertions. Genetic distance was calculated as: cM = 100×(2×(N_KG_/N_total_)) (Table 1), where N_KG_ is the number of Kan-Bar double-resistant backcross progeny and N_total_ is the total number of backcross progeny screened. We used Col/Ler SSLP genotyping markers to confirm that surviving progeny had experienced a crossover between the *MRC* insertions. Recombinants were selected using SSLP markers to restrict analysis to the *MRC1* and *MRC5* intervals (S2 and S3 Tables). Genomic DNA from these individuals was then genotyped using sets of Col/Ler SNP markers within *MRC1* and *MRC5* (KBiosciences). To test whether crossover distributions were significantly different from random we performed a 2×n *X*^*2*^ test, where n = the number of map intervals. To test the significance of individual intervals *X*^*2*^ tests were performed between the observed crossover and non-recombinant counts with those expected at random using 2×2 contingency tables (S2 and S3 Tables). Random expectations were obtained by proportionally allocating crossovers to intervals according to their physical length, i.e. an interval twice as long would be expected to receive twice as many crossovers. Finally, a false discovery correction was applied due to multiple testing [78].

### Genotyping by sequencing and crossover analysis

DNA was extracted using CTAB and used to generate sequencing libraries as described [79,80], with the following modifications. DNA was extracted from 3 rosette leaves of 5 week old plants and 150 ng of DNA used as input for each library. DNA shearing was carried out for 20 minutes at 37°C with 0.4U of DNA Shearase (Zymo research). Each set of 96 libraries was sequenced on one lane of an Illumina NextSeq500 instrument (300-cycle Mid Output run). Sequencing data was analysed to identify crossovers as previously reported, using the TIGER pipeline [79,80]. The fastq files for these data have been uploaded to ArrayExpress accession E-MTAB-4657.

To compare gene groups we sampled from the non-NBS-LRR genes by taking *n* blocks of length *b* randomly distributed in the genome and sampled a proportion *p* of genes within each block. We compared the distributions of the distance between the start coordinates of the sampled genes and the physical widths of the sampled genes, to those observed in the NBS-LRR genes, performing parameter sweeps on *n*, *b* and *p*, in order to minimize the sum of the Kolmogorov-Smirnov statistics observed for each distribution. The minimal difference between the distributions was observed for 220 blocks of 35,000 base pairs and sampling 20% of the genes in each block. Using these parameters, we obtained an approximation to the clustering structure of the NBS-LRR genes (S2A and S2B Fig). We then repeatedly sampled (3,200 times) using these parameters and tested for overlap between samplings and crossovers, requiring at least one base in common to score an overlap. Values were length-normalized by rescaling the observed data by the total length in base pairs of the sample or gene set (S3C–S3F Fig). The observed overlap between NBS-LRR and non-NBS-LRR genes with crossovers were then compared to the sampled data (S3C–S3F Fig).

### *RAC1* hotspot pollen-typing and crossover sequencing

The *RAC1/RPP9* gene and surrounding region were PCR amplified and Sanger sequenced from the Ler-0 line used in our laboratory to identify all associated SNPs and indel polymorphisms. Using this information we designed allele specific oligonucleotides (ASOs) that specifically amplify from either Col or Ler genomic DNA templates, as described previously [37,83,84,136] (S1 Fig and S19 Table). For *RAC1* two nested ASO amplifications were performed with the following sets of ASO primer pairs to amplify either crossover (Ler-Col) or parental (Col-Col) molecules:

1^st^ Crossover amplification = KC459 + KC418
1^st^ Parental amplification = KC493 + KC418
2^nd^ Crossover amplification = KC465 + KC417
2^nd^ Parental amplification = KC495 + KC417

The same PCR conditions were used for 1^st^ and 2^nd^ PCRs, which were:

94°C for 2’30”,

then 5 cycles of {94°C for 30”, 65°C (decrease by 0.4°C per cycle) for 45”, 68°C for 8’00”},

then 25 cycles of {94°C for 20”, 63°C for 30”, 68°C for 8’00”},

then {68°C for 10’00”}.

Recombination rate was estimated by titration of crossover and parental molecule concentrations from F_1_ pollen genomic DNA [37,83,84,136]. DNA concentrations were identified which gave approximately half crossover positive amplifications, where by Poisson approximation the majority are from single molecules [37,83,84,136]. Single crossover molecules were amplified and treated with exonuclease I (NEB, M0293) and shrimp alkaline phosphatase (Amersham, E70092), and then directly Sanger sequenced to identify internal crossover locations.

Based on crossover molecule concentrations measured by titration, we performed mass amplification (672 reactions) of crossovers, such that most contained between 1–2 crossover templates. The resulting amplification products were pooled, concentrated by isopropanol precipitation and gel purified. ~2 μg of DNA in 100 μl of TE (10 mM Tris, 1mM EDTA, pH 8.0) buffer was then sonicated to a size range of 300–400 bp using a Bioruptor (Diagenode). Sonicated DNA was gel purified, end-repaired and used to generate libraries (Tru-seq, Illumina). These libraries were sequenced on an Illumina nextSeq instrument using paired-end 75 bp reads. Paired reads were separated and aligned to the Col or Ler parental sequences using bowtie allowing only exact matches [137]. Reads were filtered for those that uniquely aligned to one parental sequence only. We then filtered for read pairs that had a centromere-proximal match to Col and a centromere-distal match to Ler on opposite strands, in order to identify crossover read pairs (S16 Table). These criteria are consistent with the ASO configuration used during allele-specific PCR amplification. A value of 1 was divided by the distance (bp) between each crossover read pair. For example, if a given pair of crossover reads are 200 bp apart, then the fractional value per bp is 1/200 = 0.005. Further consider 2 SNPs located between these crossover reads that divide this region into 3 intervals of 10, 50 and 140 bp. Each interval would then be assigned values of 10*0.005, 50*0.005 and 140*0.005 respectively. This process is repeated for all read pairs and final values normalized by the total number of crossover read pairs. The *RAC1* pollen-typing fastq files have been uploaded to ArrayExpression accession E-MTAB-4556.

### Analysis of historical crossover frequency

Diallelic SNPs with less than 10% missing data were selected from 80 Eurasian and 180 Swedish accession 1,001 Genomes Project datasets [72,73]. SNPs overlapping repeats and the centromeres were masked due to difficulties in assembling these regions, as performed previously [37]. SNP data was analysed using LDhat run parameters described previously, but using a θ = 0.001 look-up table [37,74]. LDhat recombination maps were filtered by setting regions where 4*N*_*e*_*r* >100, or the distance between SNPs >50 kb, to zero recombination. Population-scaled recombination rates were rescaled to cM/Mb by regression onto a consensus experimental F_2_ genetic map, as described previously [37].

### Micrococcal nuclease sequencing

Chromatin from 1 gram of wild type (Col-0) flowers (unopened buds, prior to floral stage 12) was isolated and digested with 0.05 units of mirococcal nuclease (MNase, NEB) in reaction butter (10 mM Tris-HCl, pH 8.0, 10 mM NaCl, 1 mM EDTA, 4 mM CaCl_2_) at 37°C for 15 minutes, with occasional vortexing. The digested nucleosomal DNA was then treated with proteinase K (20 μg/μl) and phenol/chroloform extraction performed, followed by ethanol precipitation. The digested DNA was separated using gel electrophoresis through an ethidium-stained agarose-TBE gel and the ~150 bp nucleosomal band was gel purified. This DNA was end-repaired and used to generate Illumina sequencing libraries (Tru-seq) and subjected to paired-end 100 bp sequencing on a Hi-seq instrument. Reads were mapped using Bowtie2 to the TAIR10 assembly and normalized by library size. Nucleosome occupancy was analysed using the nucleR package [93]. The MNase-seq fastq files have been uploaded to ArrayExpression accession E-MTAB-4556.

### NBS-LRR gene Southern blotting and hybridization

1.5 μg of genomic DNA was digested using 100 units of restriction enzyme (NEB) in a final volume of 200 μl for 15 hours at 37°C. Digested DNA samples were isopropanol precipitated, resuspended in TE and electrophoresed in 0.8% agarose-TBE gels. Gels were blotted onto a positively charged nylon membrane (Hybond-N+, Amersham Biosciences), which was hybridized in 0.5 M phosphate buffer, 7% w/v SDS, 1 mM EDTA (pH 8) and 1% BSA at 65°C. DNA probes to the *RAC1* gene (a PCR fragment amplified with RAC1-F 5′-TGATAGAGATTGAGGCGGTCA-3′ and RAC1-R 5′-GGCTTCGTCATCTTCTTCTCAC-3′), *RPP8* (a PCR fragment amplified with RPP8-F 5′-TCTTCATTCCTTCAATCTTCAGT-3′ and RPP8-R 5′-GAGATACTTGAGTTTATACGAGGCTAA-3′), *HRG1* (a PCR fragment amplified with HRG1-F 5′-GGTTTGGTCATGGAAGTCGG-3′ and HRG1-R 5′-TCTTACCCGGTTTCCTTCCC-3′), *RPP1* (a PCR fragment amplified with RPP1-F 5′-AGTCAGCAAGAGGAGACCAC-3′ and RPP1-R 5′-TCCAACTTAGTGCAATCATGGTC-3′) or *RPP4* (a PCR fragment amplified with RPP4-F 5′-AGAAGGCAAACGCTTCACTG-3′ and RPP4-R 5′-AGCTGCCATCTCAAGGTCTT-3′) were labelled with [α-32P]-dCTP using a random priming labelling kit (Rediprime II DNA labelling system, Amersham). Blots were washed with 0.5% SSC, 0.1% SDS solution at 65°C and imaged with a PhosphoImager (Typhoon 8610, Molecular Dynamics). We compared Southern blot data with the TAIR10 Col assembly and a Ler assembly generated by Pacific Biosystems (http://blog.pacificbiosciences.com/2013/08/new-data-release-arabidopsis-assembly.html) [94].

### Measurement of crossovers using flow cytometry of fluorescent pollen

Analysis of fluorescent pollen and segregation of *I5a* FTL T-DNAs was performed as described previously [67]. Genetic distance is calculated as cM = 100×(R5/(R3+R5)), where R5 is the number of yellow-alone pollen and R3 is the number of both yellow and red fluorescent pollen. To test whether recombinant and non-recombinant counts were significantly different between replicate groups we used a generalized linear model (GLM). We assumed the count data is binomially distributed:

$$Y_{i}\sim B\left(n_{i},p_{i}\right)$$

where *Y*_*i*_ represents the recombinant counts, *n*_*i*_ are the total counts, and we wish to model the proportions *Y*_*i*_/*n*_*i*_. Then:

$$E\left(Y_{i}/n_{i}\right)=p_{i}$$

and,

$$var\left(Y_{i}/n_{i}\right)=\frac{p_{i\left(1-p_{i}\right)}}{n_{i}}$$

Thus, our variance function is:

$$V\left(\mu_{i}\right)=\mu_{i}\left(1-\mu_{i}\right)$$

and our link function must map from (0,1) -> (-∞, ∞). We used a logistic link function which is:

$$\text{g}(\mu_{i})\;=\text{ logit}(\mu_{i})\;=\text{ log}\frac{\mu_{i}}{1-\mu_{i}}=\beta X+\varepsilon_{i}$$

where *ε*_*i*_ ~ *N*(0,*σ*^2^). Both replicates and genotypes are treated as independent variables (***X***) in our model. We considered *P* values less than 0.05 as significant.

## Supporting Information

S1 Fig Identifying single locus KAN and BAR lines for MRC genetic mapping. **(A)** Representative Southern blots used to identify single copy SAIL and SGT lines. Replicate samples indicate DNA isolated from siblings. Single copy lines were identified for further experiments. **(B)** Self-fertilization of homozygous KAN or BAR lines should yield ~100% resistance progeny on selective plates. **(C)** Self-fertilization of hemizygous KAN or BAR lines should yield 75% resistant progeny on selective plates. **(D)** Backcrossing hemizygous KAN or BAR lines should yield 50% resistant progeny on selective plates.
(TIFF)

S2 Fig Overlap of crossovers with NBS-LRR genes compared with other genes. **(A)** The distribution of distances between start coordinates for NBS-LRR genes (black) and sampled data (blue). **(B)** As for (A), but showing the distribution of gene widths. **(C)** Boxplot showing the distribution of the number of crossovers per gene from the sampled data. The red point shows the observed value for NBS-LRR genes and the blue point shows that for the non-NBS-LRR genes. **(D)** As for (C), but showing the number of crossovers overlapping the sampled genes, normalized by the total length of all genes in the sample. **(E)** Boxplot showing the distribution of the proportion of genes overlapping crossovers in the sampled data. The red point shows the observed value for NBS-LRR genes and the blue point shows that for the non-NBS-LRR genes. **(F)** As for (E), but showing the number of genes overlapping crossovers normalized by the total length of all genes in the sample.
(TIFF)

S3 Fig Validation of template specificity for *RAC1* allele-specific oligonucleotides used for pollen-typing. Representative ethidium bromide stained agarose gels are shown with PCR amplification products generated using either Col or Ler genomic DNA as a template. Reactions were repeated using the gradient of annealing temperatures indicated at the top of the gel. Primers were either template non-specific (universal forward (UF) or universal reverse (UR)) or allele-specific (Col R1 = KC418, Col R2 = KC417, Ler F1 = KC459, Ler F2 = KC465). Primer sequences are provided in S19 Table. Amplification was specific to the template genotype of choice under these PCR conditions.
(TIFF)

S4 Fig *HRG1* structural diversity between Arabidopsis accessions analysed by Southern blotting and hybridization. Genomic DNA was isolated from the indicated accessions and digested with *Eco*RV. DNA was separated using gel electrophoresis, blotting and probing using radio-labelled *HRG1* DNA.
(TIFF)

S5 Fig *RPP4-RPP5* structural diversity between Arabidopsis accessions analysed by Southern blotting and hybridization. Genomic DNA was isolated from the indicated accessions and digested with *Ase*I. DNA was separated using gel electrophoresis, blotting and probing using radio-labelled *RPP4* DNA.
(TIFF)

S6 Fig *RPP1* structural diversity between Arabidopsis accessions analysed by Southern blotting and hybridization. Genomic DNA was isolated from the indicated accessions and digested with *Hae*II and *Pst*I. DNA was separated using gel electrophoresis, blotting and probing using radio-labelled *RPP1* DNA.
(TIFF)

S1 Table *Arabidopsis thaliana* NBS-LRR gene family annotation. For each gene, chromosome, transcriptional start site (TSS) and termination site (TTS) are listed according to TAIR10 representative gene models. Historical recombination (cM/Mb) and diversity parameters (θ and π) were calculated for intragenic regions between TSS and TTS, using Eurasian and Swedish SNP datasets [72–74,82]. For *R* genes with known biological functions their given name is listed. In addition 3 columns have been added with TRUE/FALSE statements depending if the gene product has a match to the listed Pfam domain (TIR, NBS, LRR). Information is given on *R* gene locus structure, classified as either singleton, tandem, inverted or complex. The number of members within a repeated locus is listed.
(XLSX)

S2 Table Crossover frequency within the *MRC1* NBS-LRR supercluster region. The table lists marker coordinates used to genotype double-selected *MRC1* crossover individuals, together with Col and Ler genotypes and interval lengths (bp). The number of crossovers identified in each interval is shown, together with cM/Mb. Eurasian and Swedish historical recombination rates estimated by LDhat are shown for the same intervals and NBS-LRR genes present in each interval are listed. A chi-square test was performed between the observed *MRC* crossover counts per interval and those expected at random using a 2×2 contingency table. *P*<0.05 values are listed in the *P* column, or listed as not significantly different (ns). The *P* adj. column shows the significance level after correction for multiple testing [78].
(DOCX)

S3 Table Crossover frequency within the *MRC5* NBS-LRR supercluster region. The table lists marker coordinates used to genotype double-selected *MRC5* crossover individuals, together with Col and Ler genotypes and interval length (bp). The number of crossovers identified in each interval is shown, together with cM/Mb. Eurasian and Swedish historical recombination rates estimated by LDhat are shown for the same intervals and *R* genes present in each interval are listed. A chi-square test was performed between the observed crossover counts per interval and those expected at random using a 2×2 contingency table. *P*<0.05 values are listed in the *P* column, or listed as not significantly different (ns). The *P* adj. column shows the significance level after correction for multiple testing [78].
(DOCX)

S4 Table Fine-mapping crossovers within the *HRG1 MRC1* map interval using dCAPs genotyping. The ‘Genotyping Assay’ column indicates whether a given marker coordinate was genotyped by KBiosciences (SNP), or via dCAPs assays.
(DOCX)

S5 Table Fine-mapping crossovers within the *HRG2 HRG3 MRC5* map interval using dCAPs genotyping. The ‘Genotyping Assay’ column indicates whether a given marker coordinate was genotyped by KBiosciences (SNP), or via dCAPs assays.
(DOCX)

S6 Table Fine-mapping crossovers within the *WRR4 MRC1* map interval using dCAPs genotyping. The ‘Genotyping Assay’ column indicates whether a given marker coordinate was genotyped by KBiosciences (SNP), or via dCAPs assays.
(DOCX)

S7 Table Fine-mapping crossovers within the *CW9 MRC1* map interval using dCAPs genotyping. The ‘Genotyping Assay’ column indicates whether a given marker coordinate was genotyped by KBiosciences (SNP), or via dCAPs assays.
(DOCX)

S8 Table Fine-mapping crossovers within the *HRG4-HRG5 MRC1* map interval using dCAPs genotyping. The ‘Genotyping Assay’ column indicates whether a given marker coordinate was genotyped by KBiosciences (SNP), or via dCAPs assays.
(DOCX)

S9 Table Fine-mapping crossovers within the *HRG6 MRC1* map interval using dCAPs genotyping. The ‘Genotyping Assay’ column indicates whether a given marker coordinate was genotyped by KBiosciences (SNP), or via dCAPs assays.
(DOCX)

S10 Table Fine-mapping crossovers within the *HRG7-HRG8 MRC5* map interval using dCAPs genotyping. The ‘Genotyping Assay’ column indicates whether a given marker coordinate was genotyped by KBiosciences (SNP), or via dCAPs assays.
(DOCX)

S11 Table Fine-mapping crossovers within the *HRG9 MRC5* map interval using dCAPs genotyping. The ‘Genotyping Assay’ column indicates whether a given marker coordinate was genotyped by KBiosciences (SNP), or via dCAPs assays.
(DOCX)

S12 Table Recombination rates of plant crossover hotspots. The mean and maximum crossover frequency (cM/Mb) measured at known plant hotspots is listed, together with the species studied, hotspot name, width of measured region, genetic distance (cM) and crossover frequency (cM/Mb), together with chromosome average recombination rates.
(DOCX)

S13 Table Crossovers identified by genotyping by sequencing in a Col×Ler F_2_ population. 192 Col×Ler F_2_ individuals were analysed by genotyping-by-sequencing. The number of crossovers (CO) per F_2_ and in total are listed, for the whole genome and for each chromosome separately.
(DOCX)

S14 Table Measurement of *RAC1* recombination rate via pollen-typing. A 9.419 kb amplicon (Chr1: 11,288,146–11,297,565 bp) containing the *RAC1* gene was amplified using allele-specific oligonucleotides to estimate the number of parental (non-recombinant) and crossover molecules per μl of Col/Ler F_1_ pollen genomic DNA. Genetic distance was calculated as described [83].
(DOCX)

S15 Table Crossover distributions across the *RAC1 R* gene hotspot analysed via pollen typing. 181 single *RAC1* crossover molecules were Sanger sequenced to identify recombination sites to the resolution of single polymorphisms. Col and Ler genotypes and number of crossovers per interval are listed, together with cM/Mb.
(DOCX)

S16 Table *RAC1* crossover read pair analysis pipeline. The number of read pairs surviving sequential analysis filters are listed in order to identify *RAC1* crossover read pairs. Paired end reads (1 and 2) were separated and aligned to the Col or Ler *RAC1* parental template sequences, allowing only exact matches (Mapped). Read pair ends that mapped to both Col and Ler were then excluded (Unique). Read pair ends (1 and 2) that mapped to Col and Ler were identified (Matched), where the Ler mapping read had a lower coordinate than the Col mapping read (Orientate), and that were on opposite strands (Strand). These filters yielded 182,909 crossover read pairs.
(DOCX)

S17 Table CTT-repeat motifs associated with high and low *MRC* recombination NBS-LRR genes. NBS-LRR genes located within the *MRC* genetic maps were divided into two groups according to whether their interval had higher or lower crossover frequency (cM/Mb) compared with the male Col×Ler genome average (4.82 cM/Mb) [71]. We then matched the position weight matrix of a CTT-repeat motif previously identified as enriched at Arabidopsis historical crossover hotspots (CTTCTTCTTCTTCTTC) [37] to +1 kb windows around NBS-LRR gene transcriptional start sites (TSSs), allowing matches with >80% identity. The location, width and sequence of matching CTT motifs are listed. Motif coordinates are given relative to the 1 kb of sequence matched to.
(DOCX)

S18 Table Measurement of *I5a* genetic distance using fluorescent pollen and flow cytometry. The first column lists the accession the *I5a* reporter (Col-0) was crossed to. In the case of Col-0 this represents data from Col-0/Col-0 homozygotes. The total number of pollen counted is listed, in addition to the number with red fluorescence alone (Red), yellow alone (yellow), both colors (Red+Yellow), or neither fluorescence (None). Genetic distance is calculated as cM = 100 × (Y/(Y+RY)). Where Y is the number of yellow alone pollen grains and RG is the number of both yellow and red fluorescent pollen grains. To test whether recombinant and non-recombinant counts were significantly different between replicate groups we used a generalized linear model (GLM), assuming that the count data is binomially distributed. Tests were performed between F_1_ genotypes and Col/Col homozygotes.
(DOCX)

S19 Table *RAC1* primer sequences used for allele-specific PCR amplification. Positions annealing to Col/Ler polymorphic bases in the allele-specific primers are indicated in red. The accession each primer anneals to is listed (Col or Ler), together with a code indicating whether the primer is forward or reverse (F or R) and used in the first or second round of allele-specific amplification (1 or 2).
(DOCX)
