# Supplementary figures and images for: Recombination Rate Heterogeneity within Arabidopsis Disease Resistance Genes
Source: PLoS Genet. 2016 Jul 14;12(7):e1006179. doi: 10.1371/journal.pgen.1006179 (PMC4945094; doi:10.1371/journal.pgen.1006179)

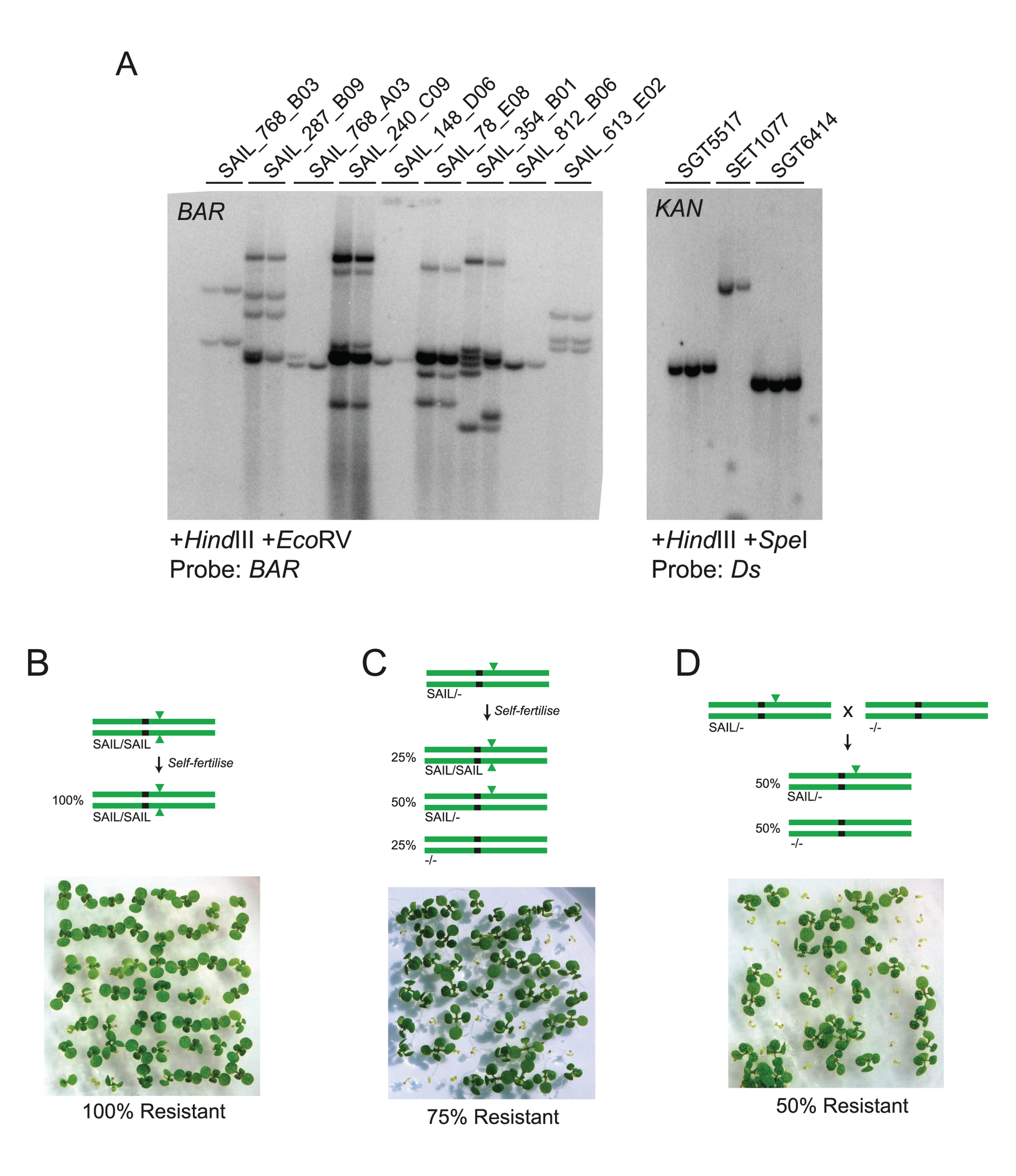

Supplement: S1 Fig — (A) Representative Southern blots used to identify single copy SAIL and SGT lines. Replicate samples indicate DNA isolated from siblings. Single copy lines were identified for further experiments. (B) Self-fertilization of homozygous KAN or BAR lines should yield ~100% resistance progeny on selective plates. (C) Self-fertilization of hemizygous KAN or BAR lines should yield 75% resistant progeny on selective plates. (D) Backcrossing hemizygous KAN or BAR lines should yield 50% resistant progeny on selective plates. (TIFF) [file pgen.1006179.s001.tiff]

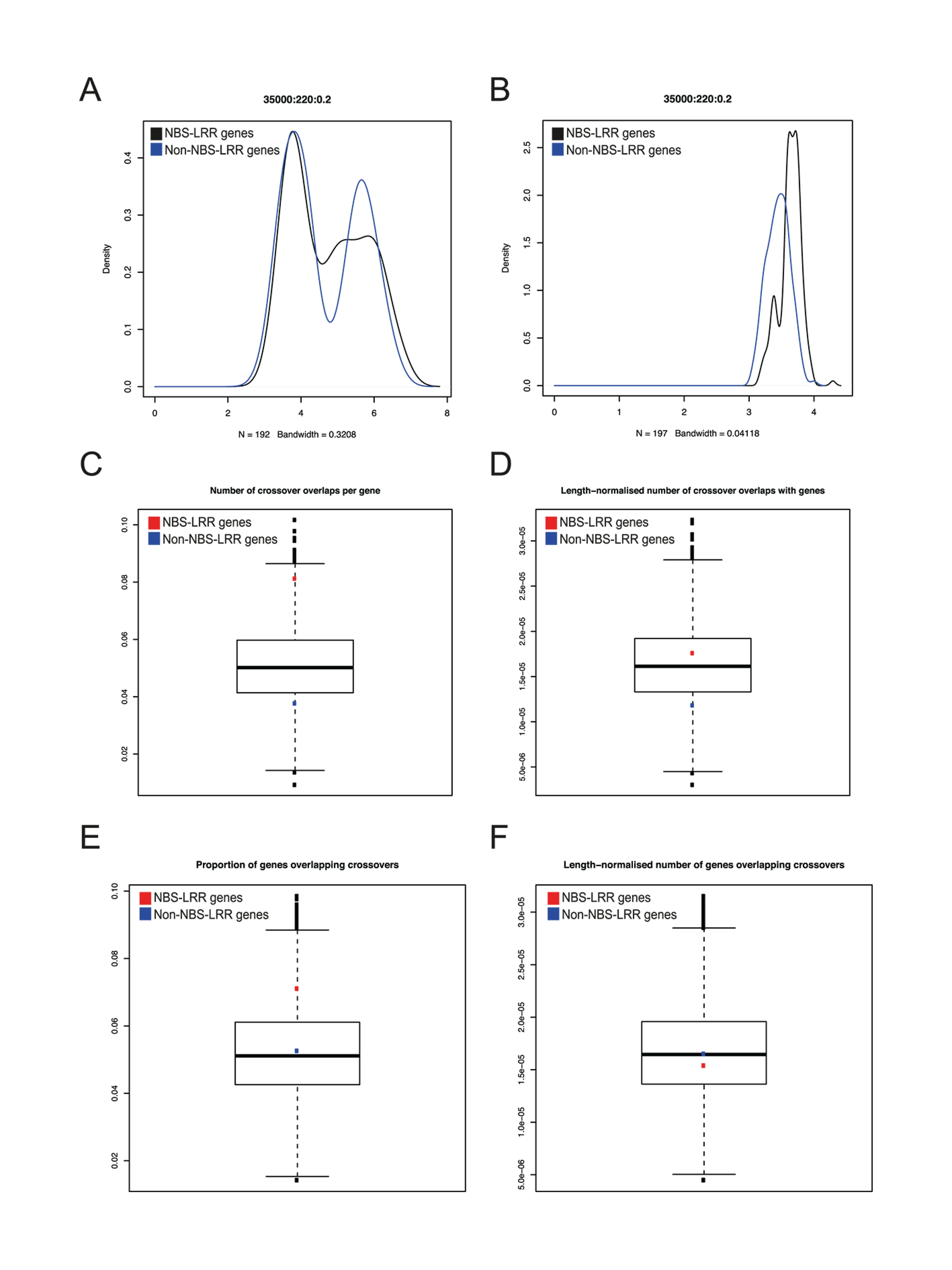

Supplement: S2 Fig — (A) The distribution of distances between start coordinates for NBS-LRR genes (black) and sampled data (blue). (B) As for (A), but showing the distribution of gene widths. (C) Boxplot showing the distribution of the number of crossovers per gene from the sampled data. The red point shows the observed value for NBS-LRR genes and the blue point shows that for the non-NBS-LRR genes. (D) As for (C), but showing the number of crossovers overlapping the sampled genes, normalized by the total length of all genes in the sample. (E) Boxplot showing the distribution of the proportion of genes overlapping crossovers in the sampled data. The red point shows the observed value for NBS-LRR genes and the blue point shows that for the non-NBS-LRR genes. (F) As for (E), but showing the number of genes overlapping crossovers normalized by the total length of all genes in the sample. (TIFF) [file pgen.1006179.s002.tiff]

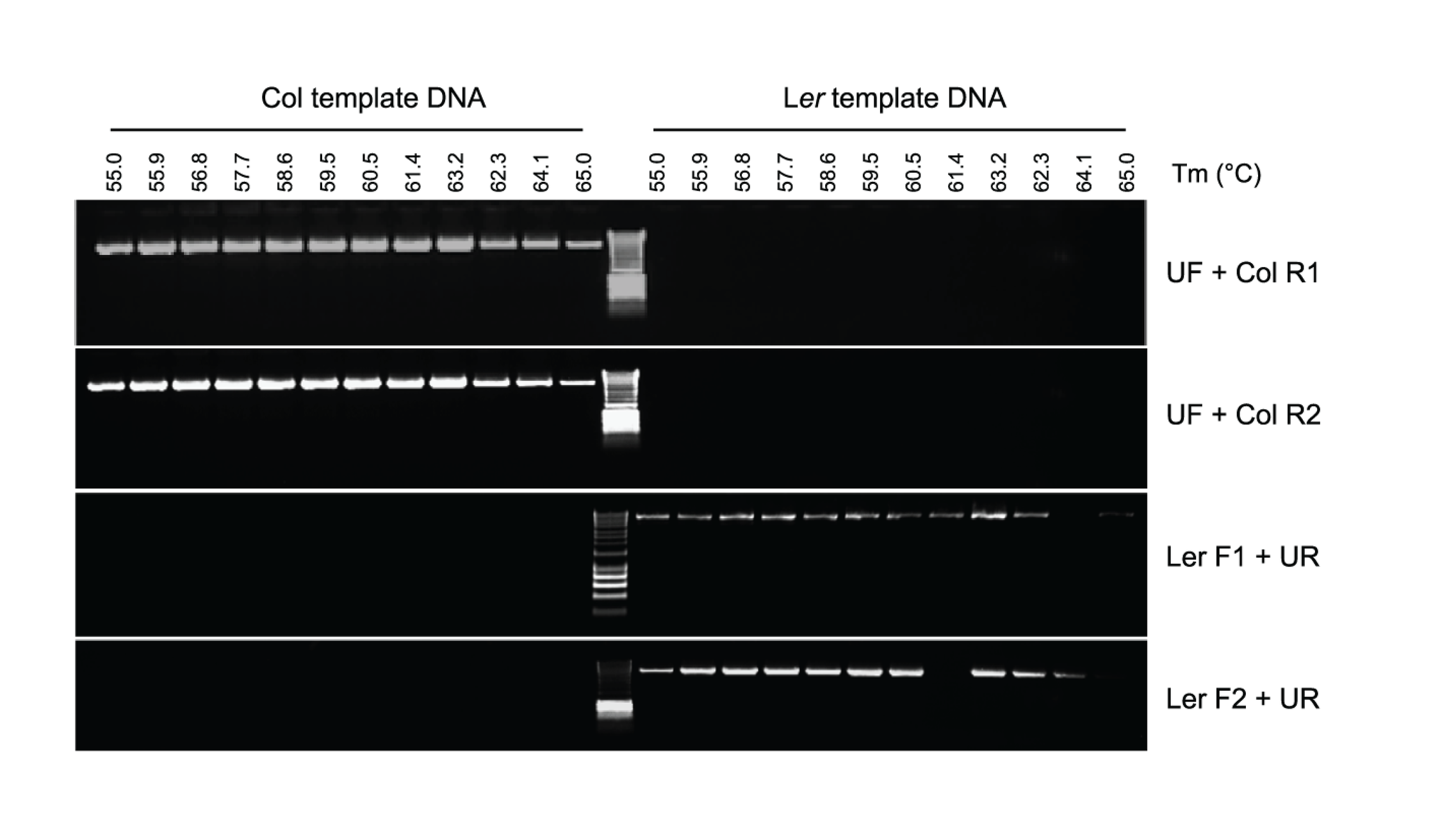

Supplement: S3 Fig — Representative ethidium bromide stained agarose gels are shown with PCR amplification products generated using either Col or Ler genomic DNA as a template. Reactions were repeated using the gradient of annealing temperatures indicated at the top of the gel. Primers were either template non-specific (universal forward (UF) or universal reverse (UR)) or allele-specific (Col R1 = KC418, Col R2 = KC417, Ler F1 = KC459, Ler F2 = KC465). Primer sequences are provided in S19 Table. Amplification was specific to the template genotype of choice under these PCR conditions. (TIFF) [file pgen.1006179.s003.tiff]

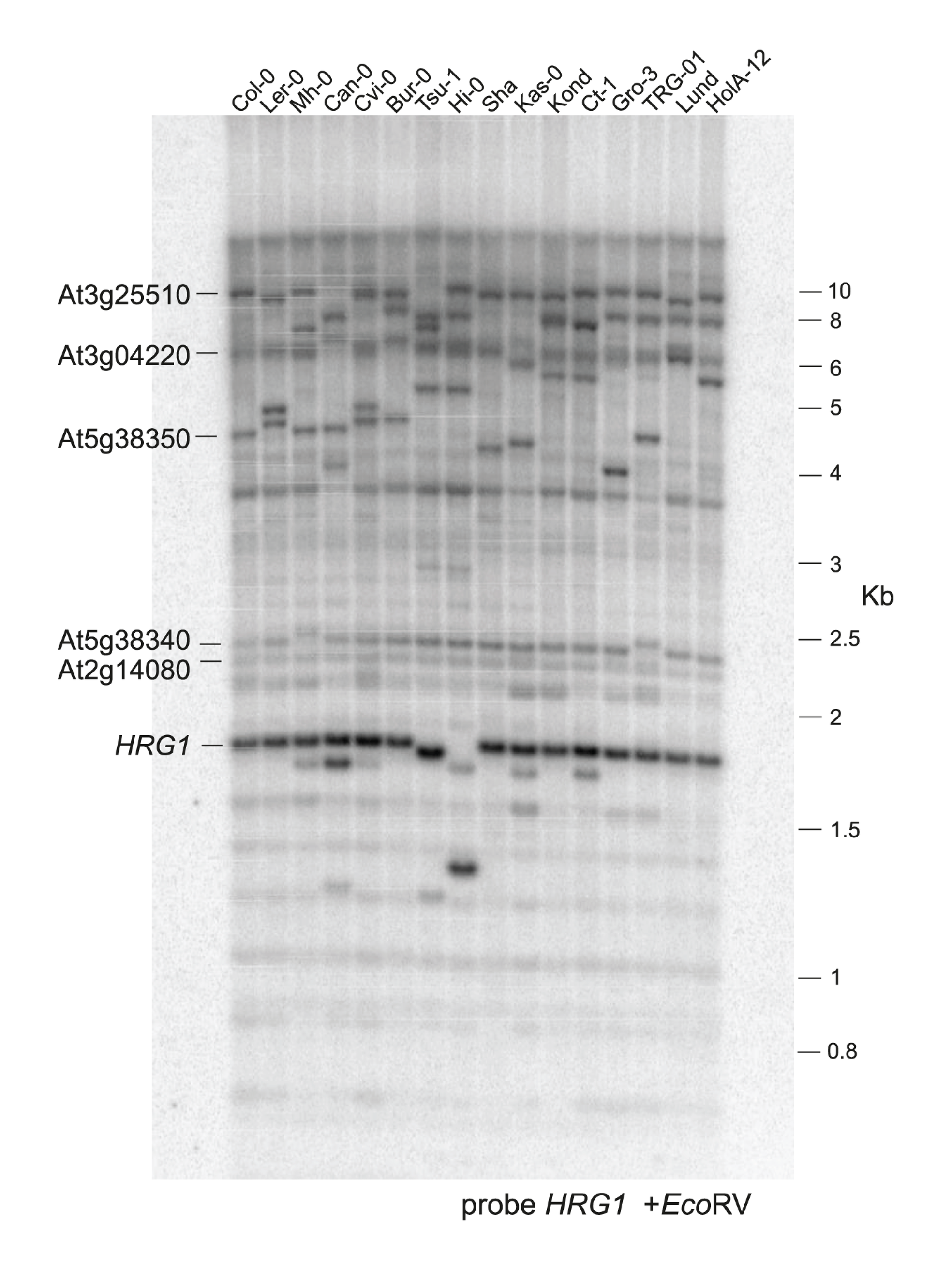

Supplement: S4 Fig — Genomic DNA was isolated from the indicated accessions and digested with EcoRV. DNA was separated using gel electrophoresis, blotting and probing using radio-labelled HRG1 DNA. (TIFF) [file pgen.1006179.s004.tiff]

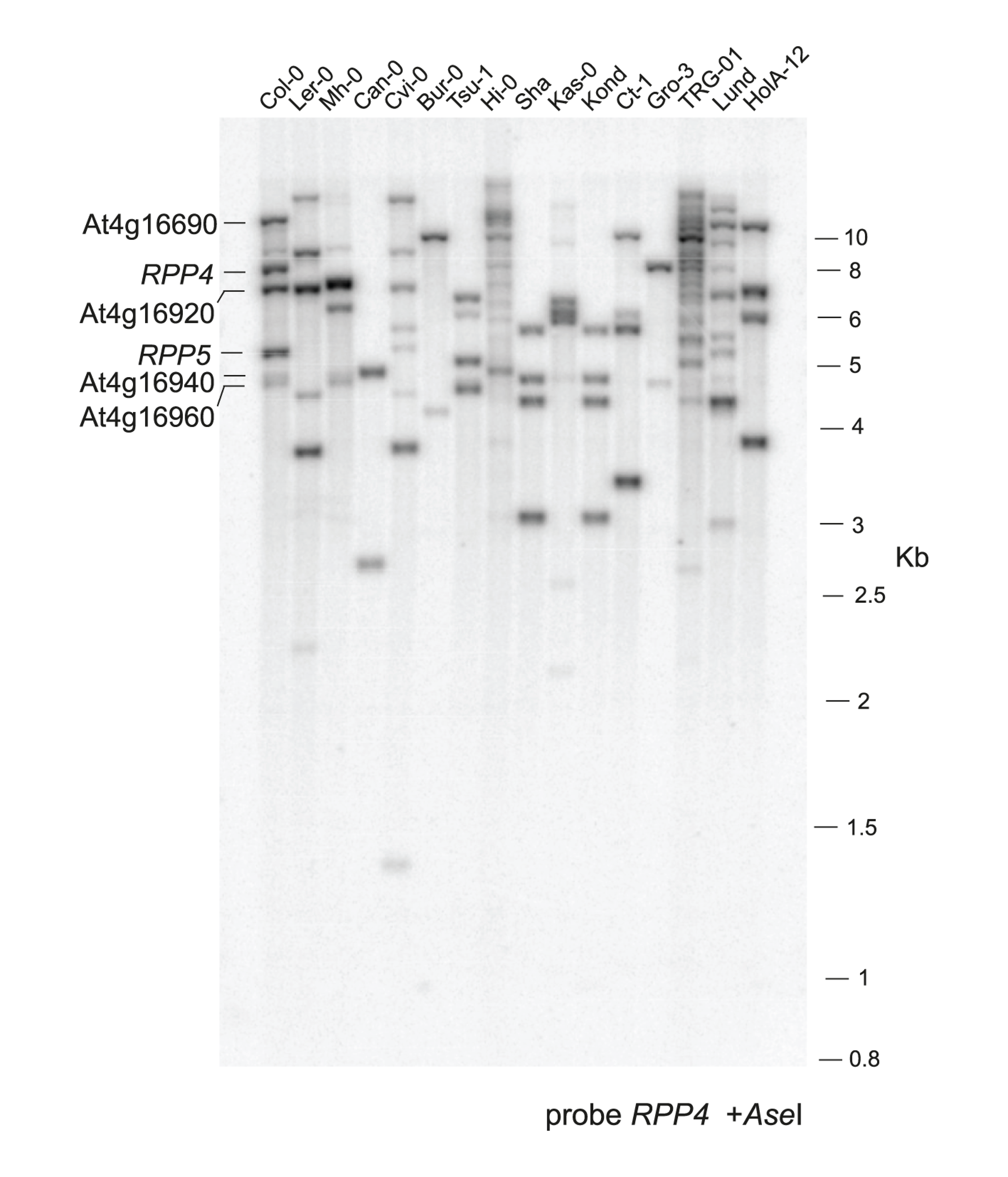

Supplement: S5 Fig — Genomic DNA was isolated from the indicated accessions and digested with AseI. DNA was separated using gel electrophoresis, blotting and probing using radio-labelled RPP4 DNA. (TIFF) [file pgen.1006179.s005.tiff]

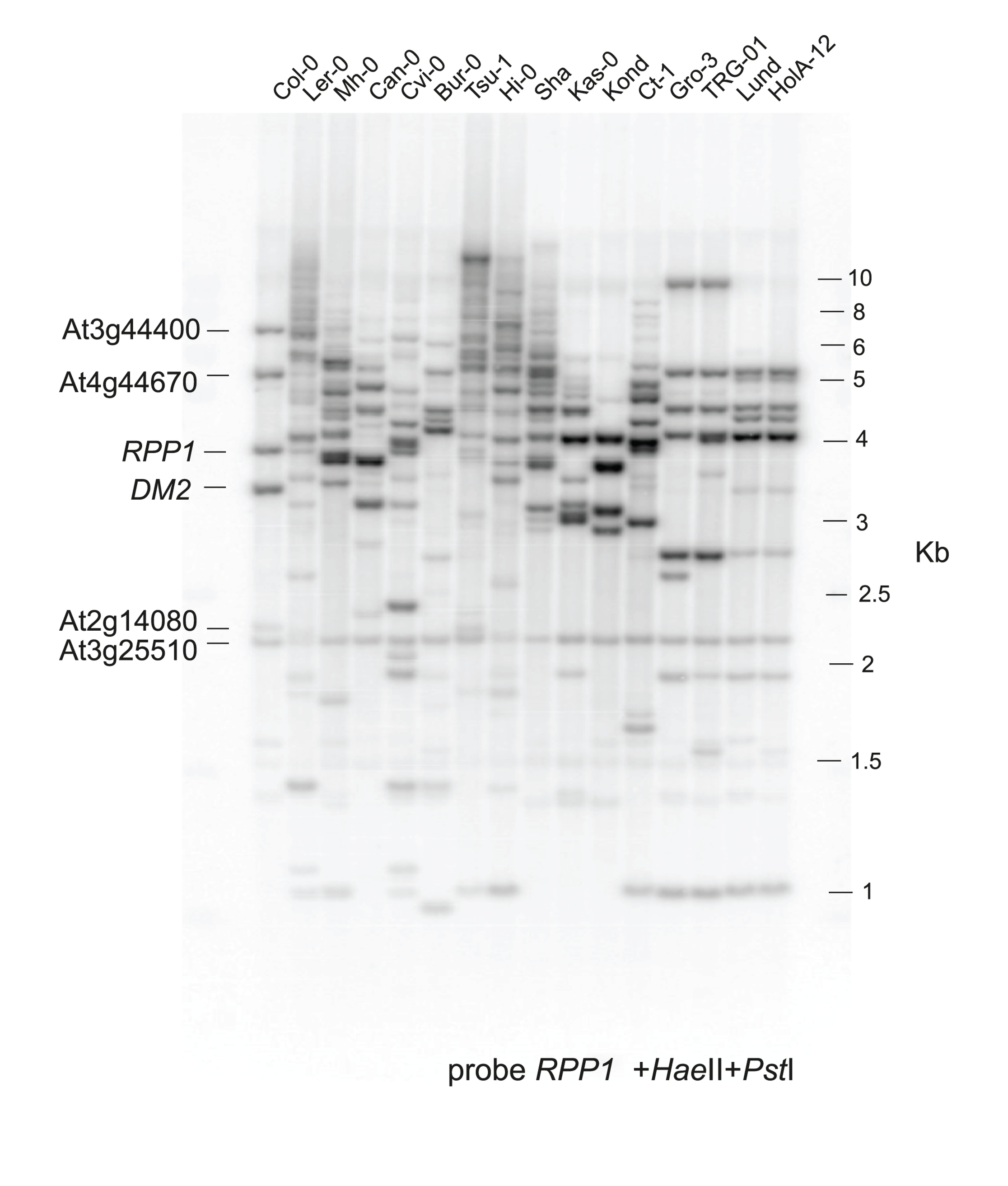

Supplement: S6 Fig — Genomic DNA was isolated from the indicated accessions and digested with HaeII and PstI. DNA was separated using gel electrophoresis, blotting and probing using radio-labelled RPP1 DNA. (TIFF) [file pgen.1006179.s006.tiff]
