# Supplementary material for: Recombination Rate Heterogeneity within Arabidopsis Disease Resistance Genes
Source: PLoS Genet. 2016 Jul 14;12(7):e1006179. doi: 10.1371/journal.pgen.1006179 (PMC4945094; doi:10.1371/journal.pgen.1006179)
Supplement: S2 Table — The table lists marker coordinates used to genotype double-selected MRC1 crossover individuals, together with Col and Ler genotypes and interval lengths (bp). The number of crossovers identified in each interval is shown, together with cM/Mb. Eurasian and Swedish historical recombination rates estimated by LDhat are shown for the same intervals and NBS-LRR genes present in each interval are listed. A chi-square test was performed between the observed MRC crossover counts per interval and those expected at random using a 2×2 contingency table. P<0.05 values are listed in the P column, or listed as not significantly different (ns). The P adj. column shows the significance level after correction for multiple testing [78]. (DOCX) [file pgen.1006179.s008.docx]

**S2 Table. Crossover frequency within the *MRC1* NBS-LRR supercluster region.**

| Chr1 coordinates | Col | Ler | Interval (bp) | COs | *P* | *P* adj. | ColxLer cM/Mb | Eurasian cM/Mb | Swedish cM/Mb | NBS-LRR genes |
| --- | --- | --- | --- | --- | --- | --- | --- | --- | --- | --- |
| 20768571 | NULL | SGT | 1888 | 0 | ns | ns | 0.00 | 0.91 | 2.03 |  |
| 20770459 | T | G | 51330 | 5 | ns | ns | 3.06 | 2.70 | 2.86 |  |
| 20821789 | T | C | 44589 | 6 | ns | ns | 4.23 | 2.92 | 1.84 |  |
| 20866378 | T | G | 55906 | 0 | 0.003 | 0.062 | 0.00 | 1.77 | 1.68 |  |
| 20922284 | A | C | 44196 | 8 | ns | ns | 5.69 | 2.49 | 3.24 |  |
| 20966480 | T | A | 57617 | 7 | ns | ns | 3.82 | 2.73 | 1.08 |  |
| 21024097 | C | A | 36457 | 1 | ns | ns | 0.86 | 3.47 | 5.46 |  |
| 21060554 | T | G | 35127 | 4 | ns | ns | 3.58 | 2.53 | 1.78 |  |
| 21095681 | T | G | 47454 | 5 | ns | ns | 3.31 | 6.32 | 2.46 |  |
| 21143135 | G | A | 21592 | 7 | ns | ns | 10.20 | 4.86 | 4.58 |  |
| 21164727 | G | C | 39001 | 14 | ns | ns | 11.29 | 5.19 | 4.30 | AT1G56510 *(WRR4*)  AT1G56520 AT1G56540 |
| 21203728 | C | T | 47570 | 5 | ns | ns | 3.31 | 3.62 | 2.60 |  |
| 21251298 | G | T | 58824 | 4 | ns | ns | 2.14 | 1.77 | 1.99 |  |
| 21310122 | T | G | 43832 | 9 | ns | ns | 6.46 | 3.75 | 6.02 | AT1G57650 |
| 21353954 | C | A | 9272 | 1 | ns | ns | 3.39 | 20.14 | 16.98 | AT1G57670 |
| 21363226 | G | A | 42509 | 7 | ns | ns | 5.18 | 4.67 | 5.72 |  |
| 21405735 | G | A | 40233 | 10 | ns | ns | 7.82 | 4.56 | 6.33 | AT1G57830 AT1G57850 |
| 21445968 | G | A | 56991 | 8 | ns | ns | 4.42 | 3.75 | 5.10 |  |
| 21502959 | A | T | 57129 | 5 | ns | ns | 2.75 | 5.44 | 5.07 |  |
| 21560088 | A | C | 40357 | 4 | ns | ns | 3.12 | 3.82 | 3.42 |  |
| 21600445 | C | T | 52196 | 0 | 0.0048 | 0.0763 | 0.00 | 3.97 | 3.53 |  |
| 21652641 | C | G | 33407 | 11 | ns | ns | 10.36 | 4.50 | 3.95 |  |
| 21686048 | C | T | 29655 | 3 | ns | ns | 3.18 | 7.75 | 8.30 | AT1G58390 AT1G58400 AT1G58410 |
| 21715703 | T | G | 49353 | 4 | ns | ns | 2.55 | 9.67 | 8.48 | AT1G58602  (*RPP7*) |
| 21765056 | A | G | 93248 | 1 | 0.0002 | 0.0276 | 0.34 | 1.20 | 1.97 | AT1G58807 AT1G58848 AT1G59124 AT1G59218 |
| 21858304 | T | C | 43645 | 8 | ns | ns | 5.77 | 8.39 | 6.80 |  |
| 21901949 | T | A | 15916 | 6 | ns | ns | 11.86 | 26.73 | 20.73 | AT1G59620  *(CW9*) |
| 21917865 | C | T | 41254 | 5 | ns | ns | 3.81 | 9.05 | 5.14 |  |
| 21959119 | A | C | 27027 | 3 | ns | ns | 3.49 | 4.39 | 4.09 |  |
| 21986146 | G | C | 15206 | 4 | ns | ns | 8.27 | 14.47 | 5.94 | AT1G59780 |
| 22001352 | G | A | 42928 | 9 | ns | ns | 6.59 | 10.15 | 7.56 |  |
| 22044280 | C | A | 48460 | 4 | ns | ns | 2.60 | 8.00 | 6.53 |  |
| 22092740 | C | T | 40752 | 5 | ns | ns | 3.86 | 3.24 | 4.68 |  |
| 22133492 | G | T | 48442 | 14 | ns | ns | 9.09 | 2.84 | 3.61 |  |
| 22181934 | C | T | 43930 | 3 | ns | ns | 2.15 | 7.38 | 6.63 |  |
| 22225864 | A | G | 17391 | 1 | ns | ns | 1.81 | 4.74 | 7.76 | AT1G60320 |
| 22243255 | C | T | 45849 | 12 | ns | ns | 8.23 | 9.73 | 9.06 |  |
| 22289104 | G | A | 50211 | 6 | ns | ns | 3.76 | 4.90 | 4.09 |  |
| 22339315 | T | G | 52308 | 5 | ns | ns | 3.01 | 6.69 | 5.79 |  |
| 22391623 | C | A | 37577 | 3 | ns | ns | 2.51 | 6.59 | 5.15 |  |
| 22429200 | T | A | 38702 | 3 | ns | ns | 2.44 | 4.07 | 3.69 |  |
| 22467902 | G | A | 43808 | 0 | 0.011 | 0.157 | 0.00 | 6.39 | 6.14 |  |
| 22511710 | T | A | 3018 | 0 | ns | ns | 0.00 | 10.70 | 5.04 | AT1G61105 |
| 22514728 | G | A | 32210 | 5 | ns | ns | 4.88 | 7.48 | 8.12 |  |
| 22546938 | T | C | 20540 | 2 | ns | ns | 3.06 | 63.13 | 51.79 | AT1G61180  AT1G61190  *(RPP39*) |
| 22567478 | C | T | 27865 | 3 | ns | ns | 3.39 | 15.51 | 15.13 |  |
| 22595343 | C | A | 40084 | 11 | ns | ns | 8.63 | 32.10 | 28.29 | AT1G61300 AT1G61310 |
| 22635427 | G | C | 64616 | 0 | 0.0013 | 0.0425 | 0.00 | 1.56 | 4.43 |  |
| 22700043 | C | G | 26399 | 5 | ns | ns | 5.96 | 8.07 | 8.26 |  |
| 22726442 | A | T | 47837 | 10 | ns | ns | 6.57 | 5.28 | 6.84 |  |
| 22774279 | T | C | 55376 | 15 | ns | ns | 8.52 | 9.04 | 11.80 |  |
| 22829655 | C | T | 51117 | 9 | ns | ns | 5.54 | 4.52 | 4.56 |  |
| 22880772 | C | T | 45671 | 9 | ns | ns | 6.20 | 8.58 | 6.32 |  |
| 22926443 | A | G | 59966 | 9 | ns | ns | 4.72 | 8.85 | 11.21 |  |
| 22986409 | C | T | 39971 | 20 | 0.0265 | 0.312 | 15.74 | 5.39 | 6.94 |  |
| 23026380 | G | A | 42852 | 17 | ns | ns | 12.48 | 4.28 | 8.34 |  |
| 23069232 | A | G | 48448 | 10 | ns | ns | 6.49 | 4.01 | 4.43 |  |
| 23117680 | A | T | 48672 | 11 | ns | ns | 7.11 | 4.07 | 5.63 |  |
| 23166352 | G | A | 41807 | 12 | ns | ns | 9.03 | 14.76 | 13.26 | AT1G62630 |
| 23208159 | C | T | 38260 | 8 | ns | ns | 6.58 | 4.80 | 3.83 |  |
| 23246419 | T | C | 44943 | 19 | ns | ns | 13.30 | 8.02 | 7.97 |  |
| 23291362 | A | T | 75355 | 17 | ns | ns | 7.10 | 11.28 | 4.06 |  |
| 23366717 | A | T | 42244 | 10 | ns | ns | 7.45 | 2.72 | 4.11 |  |
| 23408961 | A | G | 51168 | 20 | ns | ns | 12.29 | 7.59 | 4.20 |  |
| 23460129 | G | A | 53813 | 23 | 0.0359 | 0.337 | 13.44 | 20.15 | 7.52 | AT1G63350  (*HRG4*)  AT1G63360  *(HRG5*) |
| 23513942 | C | T | 38068 | 4 | ns | ns | 3.30 | 4.96 | 4.56 |  |
| 23552010 | A | G | 41763 | 7 | ns | ns | 5.27 | 3.84 | 4.46 |  |
| 23593773 | A | T | 40931 | 6 | ns | ns | 4.61 | 5.64 | 4.91 |  |
| 23634704 | A | C | 25234 | 8 | ns | ns | 9.97 | 8.44 | 7.66 | AT1G63730 AT1G63740  AT1G63750  *(HRG6*) |
| 23659938 | G | A | 39905 | 8 | ns | ns | 6.31 | 8.30 | 6.59 |  |
| 23699843 | A | G | 23768 | 7 | ns | ns | 9.26 | 2.87 | 3.38 | AT1G63860 AT1G63870  AT1G63880 |
| 23723611 | C | T | 53311 | 8 | ns | ns | 4.72 | 7.29 | 8.01 |  |
| 23776922 | C | T | 7187 | 3 | ns | ns | 13.13 | 6.45 | 4.61 | AT1G64070  *(RLM1*) |
| 23784109 | G | T | 34038 | 6 | ns | ns | 5.54 | 10.87 | 7.17 |  |
| 23818147 | G | A | 48284 | 9 | ns | ns | 5.86 | 4.72 | 5.50 |  |
| 23866431 | T | G | 40331 | 8 | ns | ns | 6.24 | 4.38 | 4.39 |  |
| 23906762 | G | A | 53190 | 9 | ns | ns | 5.32 | 5.06 | 8.07 |  |
| 23959952 | A | G | 91107 | 6 | 0.0347 | 0.337 | 2.07 | 5.21 | 4.97 |  |
| 24051059 | C | A | 59377 | 5 | ns | ns | 2.65 | 4.65 | 5.27 |  |
| 24110436 | A | G | 43980 | 5 | ns | ns | 3.58 | 7.40 | 5.24 |  |
| 24154416 | C | A | 56876 | 6 | ns | ns | 3.32 | 6.02 | 6.41 |  |
| 24211292 | C | T | 33463 | 8 | ns | ns | 7.52 | 6.99 | 15.92 |  |
| 24244755 | T | A | 44041 | 4 | ns | ns | 2.86 | 9.57 | 9.74 |  |
| 24288796 | T | C | 16071 | 0 | ns | ns | 0.00 | 5.49 | 7.08 | AT1G65390 |
| 24304867 | A | G | 38516 | 13 | ns | ns | 10.62 | 3.33 | 4.71 |  |
| 24343383 | G | C | 57626 | 21 | ns | ns | 11.46 | 5.71 | 5.75 |  |
| 24401009 | T | A | 36470 | 25 | 0.0020 | 0.0490 | 21.56 | 3.24 | 7.20 |  |
| 24437479 | T | A | 54384 | 18 | ns | ns | 10.41 | 4.89 | 6.84 |  |
| 24491863 | G | A | 11683 | 18 | 0.0009 | 0.0423 | 48.46 | 8.32 | 12.41 | AT1G65850  *(HRG1*) |
| 24503546 | C | G | 45016 | 16 | ns | ns | 11.18 | 3.14 | 4.67 |  |
| 24548562 | G | A | 49451 | 15 | ns | ns | 9.54 | 3.32 | 4.28 |  |
| 24598013 | T | A | 11454 | 3 | ns | ns | 8.24 | 3.06 | 6.50 | AT1G66090 |
| 24609467 | T | C | 29752 | 14 | ns | ns | 14.80 | 5.11 | 4.08 |  |
| 24639219 | T | C | 2567 | 0 | ns | ns | 0.00 | 0.59 | 2.79 |  |
| 24641786 | SAIL | NULL | 0 | 0 |  |  | 0.00 | 0.00 | 0.00 |  |
| Total |  |  | 3873215 | 725 |  |  | 5.89 | 5.01 | 5.60 |  |
