# Supplementary material for: Recombination Rate Heterogeneity within Arabidopsis Disease Resistance Genes
Source: PLoS Genet. 2016 Jul 14;12(7):e1006179. doi: 10.1371/journal.pgen.1006179 (PMC4945094; doi:10.1371/journal.pgen.1006179)
Supplement: S3 Table — The table lists marker coordinates used to genotype double-selected MRC5 crossover individuals, together with Col and Ler genotypes and interval length (bp). The number of crossovers identified in each interval is shown, together with cM/Mb. Eurasian and Swedish historical recombination rates estimated by LDhat are shown for the same intervals and R genes present in each interval are listed. A chi-square test was performed between the observed crossover counts per interval and those expected at random using a 2×2 contingency table. P<0.05 values are listed in the P column, or listed as not significantly different (ns). The P adj. column shows the significance level after correction for multiple testing [78]. (DOCX) [file pgen.1006179.s009.docx]

**S3 Table. Crossover frequency within the *MRC5* NBS-LRR supercluster region.**

| Chr5 coordinate | Col | Ler | Interval (bp) | COs | *P* | *P* adj. | Col x Ler cM/Mb | Eurasian cM/Mb | Swedish cM/Mb | *R* genes |
| --- | --- | --- | --- | --- | --- | --- | --- | --- | --- | --- |
| 15558820 | INDEL | INDEL | 56138 | 12 | ns | ns | 5.87 | 3.11 | 2.82 |  |
| 15614958 | T | C | 37598 | 17 | 0.0297 | 0.308 | 12.41 | 6.40 | 4.90 |  |
| 15652556 | T | A | 71517 | 4 | ns | ns | 1.54 | 1.17 | 1.65 |  |
| 15724073 | G | A | 59868 | 17 | ns | ns | 7.79 | 4.82 | 5.73 |  |
| 15783941 | C | T | 57901 | 16 | ns | ns | 7.59 | 7.54 | 5.22 |  |
| 15841842 | C | T | 53057 | 20 | 0.0373 | 0.308 | 10.35 | 6.91 | 5.08 |  |
| 15894899 | A | G | 53123 | 14 | ns | ns | 7.23 | 3.95 | 3.01 |  |
| 15948022 | C | T | 46729 | 19 | 0.0315 | 0.308 | 11.16 | 4.85 | 5.12 |  |
| 15994751 | A | G | 38391 | 10 | ns | ns | 7.15 | 4.75 | 6.02 |  |
| 16033142 | T | C | 34187 | 6 | ns | ns | 4.82 | 5.06 | 5.24 | AT5G40060  AT5G40090 AT5G40100 |
| 16067329 | C | T | 57220 | 15 | ns | ns | 7.20 | 7.31 | 4.42 |  |
| 16124549 | T | A | 50806 | 10 | ns | ns | 5.40 | 3.17 | 3.85 |  |
| 16175355 | G | C | 59461 | 5 | ns | ns | 2.31 | 5.90 | 4.92 |  |
| 16234816 | A | T | 61047 | 8 | ns | ns | 3.60 | 5.29 | 5.41 |  |
| 16295863 | T | G | 53020 | 10 | ns | ns | 5.18 | 4.17 | 3.55 |  |
| 16348883 | T | C | 42649 | 3 | ns | ns | 1.93 | 2.53 | 3.01 |  |
| 16391532 | A | C | 13334 | 0 | ns | ns | 0.00 | 1.24 | 1.79 | AT5G40910 |
| 16404866 | A | T | 54772 | 8 | ns | ns | 4.01 | 7.56 | 5.43 |  |
| 16459638 | C | A | 57995 | 2 | ns | ns | 0.95 | 5.73 | 2.58 |  |
| 16517633 | C | T | 58591 | 3 | ns | ns | 1.41 | 5.00 | 4.49 |  |
| 16576224 | T | A | 36025 | 12 | ns | ns | 9.14 | 5.96 | 7.61 |  |
| 16612249 | C | A | 19954 | 5 | ns | ns | 6.88 | 14.00 | 9.78 | AT5G41540 AT5G41550  *(DM1*) |
| 16632203 | G | A | 51925 | 14 | ns | ns | 7.40 | 10.76 | 10.00 |  |
| 16684128 | A | C | 24348 | 2 | ns | ns | 2.25 | 11.68 | 16.12 | AT5G41740  *(DM1*)  AT5G41750 |
| 16708476 | A | G | 49698 | 3 | ns | ns | 1.66 | 5.90 | 4.76 |  |
| 16758174 | G | C | 44364 | 5 | ns | ns | 3.09 | 5.92 | 6.83 |  |
| 16802538 | C | T | 56189 | 8 | ns | ns | 3.91 | 6.39 | 6.31 |  |
| 16858727 | T | A | 57828 | 9 | ns | ns | 4.27 | 5.87 | 6.11 |  |
| 16916555 | T | C | 105924 | 16 | ns | ns | 4.15 | 5.76 | 5.58 |  |
| 17022479 | T | C | 64679 | 7 | ns | ns | 2.97 | 3.11 | 4.89 |  |
| 17087158 | C | A | 57796 | 10 | ns | ns | 4.75 | 6.18 | 5.75 |  |
| 17144954 | G | A | 61476 | 12 | ns | ns | 5.36 | 8.48 | 7.50 |  |
| 17206430 | A | G | 49118 | 5 | ns | ns | 2.79 | 4.19 | 8.13 |  |
| 17255548 | C | T | 58113 | 6 | ns | ns | 2.83 | 6.24 | 7.61 |  |
| 17313661 | G | C | 55692 | 2 | ns | ns | 0.99 | 4.77 | 7.17 |  |
| 17369353 | T | C | 33368 | 0 | ns | ns | 0.00 | 4.15 | 5.84 |  |
| 17402721 | C | T | 52096 | 3 | ns | ns | 1.58 | 3.03 | 4.76 |  |
| 17454817 | A | T | 15787 | 2 | ns | ns | 3.48 | 67.13 | 59.43 | AT5G43470  *(RPP8*) |
| 17470604 | T | C | 69906 | 7 | ns | ns | 2.75 | 7.84 | 9.08 |  |
| 17540510 | A | G | 45385 | 6 | ns | ns | 3.63 | 4.78 | 6.95 | AT5G43730 AT5G43740 |
| 17585895 | T | C | 54608 | 6 | ns | ns | 3.02 | 6.86 | 9.71 |  |
| 17640503 | G | A | 67222 | 9 | ns | ns | 3.68 | 7.14 | 8.48 |  |
| 17707725 | T | C | 58878 | 9 | ns | ns | 4.20 | 4.33 | 4.75 |  |
| 17766603 | G | C | 57679 | 7 | ns | ns | 3.33 | 3.37 | 2.76 |  |
| 17824282 | G | A | 57337 | 9 | ns | ns | 4.31 | 4.11 | 6.39 |  |
| 17881619 | G | T | 65205 | 14 | ns | ns | 5.89 | 4.21 | 4.05 | AT5G44510  *(TAO1*) |
| 17946824 | T | C | 52933 | 8 | ns | ns | 4.15 | 4.31 | 3.67 |  |
| 17999757 | T | A | 65295 | 25 | 0.016 | 0.308 | 10.51 | 5.17 | 4.40 |  |
| 18065052 | A | T | 69470 | 6 | ns | ns | 2.37 | 2.89 | 2.68 | AT5G44870  *(LAZ5/TTR1*) |
| 18134522 | A | G | 34313 | 8 | ns | ns | 6.40 | 5.27 | 5.06 | AT5G44900 AT5G44910  AT5G44920  AT5G45000 |
| 18168835 | T | A | 44546 | 0 | 0.026 | 0.308 | 0.00 | 0.95 | 0.81 | AT5G45050  *(RRS1B*)  AT5G45060  *(RPS4B*)  AT5G45070 AT5G45080 AT5G45090 |
| 18213381 | A | G | 64251 | 9 | ns | ns | 3.85 | 3.52 | 3.56 |  |
| 18277632 | A | T | 58833 | 10 | ns | ns | 4.67 | 3.67 | 3.66 | AT5G45200  *(HRG7*)  AT5G45210  *(HRG8*)  AT5G45220 AT5G45230 AT5G45240  AT5G45250  *(RPS4*)  AT5G45260  *(RRS1*) |
| 18336465 | G | C | 55096 | 15 | ns | ns | 7.47 | 6.17 | 6.07 |  |
| 18391561 | A | G | 58130 | 3 | ns | ns | 1.42 | 3.81 | 2.76 | AT5G45440 AT5G45490 AT5G45510 |
| 18449691 | T | C | 54675 | 6 | ns | ns | 3.01 | 3.32 | 3.04 |  |
| 18504366 | G | A | 60607 | 13 | ns | ns | 5.89 | 3.72 | 3.73 |  |
| 18564973 | G | A | 55342 | 16 | ns | ns | 7.94 | 2.56 | 1.38 |  |
| 18620315 | G | A | 51093 | 15 | ns | ns | 8.06 | 3.15 | 2.67 |  |
| 18671408 | A | T | 41083 | 8 | ns | ns | 5.35 | 7.32 | 5.13 |  |
| 18712491 | A | G | 42622 | 9 | ns | ns | 5.80 | 5.39 | 5.13 |  |
| 18755113 | G | T | 15148 | 11 | 0.034 | 0.308 | 19.93 | 19.22 | 11.58 | AT5G46260  (*HRG2*)  AT5G46270  (*HRG3*) |
| 18770261 | C | G | 63009 | 11 | ns | ns | 4.79 | 4.29 | 5.50 |  |
| 18833270 | T | A | 43732 | 0 | 0.028 | 0.308 | 0.00 | 1.50 | 1.74 | AT5G46450  AT5G46470  (*RPS6*)  AT5G46490  AT5G46500  AT5G46510  AT5G46520 |
| 18877002 | C | A | 79700 | 20 | ns | ns | 6.89 | 4.63 | 4.62 |  |
| 18956702 | G | A | 60308 | 25 | 0.010 | 0.308 | 11.38 | 4.65 | 8.74 |  |
| 19017010 | A | T | 56878 | 10 | ns | ns | 4.83 | 1.75 | 2.49 |  |
| 19073888 | G | A | 53355 | 6 | ns | ns | 3.09 | 1.66 | 1.77 |  |
| 19127243 | G | C | 52613 | 11 | ns | ns | 5.74 | 2.71 | 3.75 |  |
| 19179856 | A | T | 24443 | 2 | ns | ns | 2.25 | 3.65 | 4.25 | AT5G47250  AT5G47260 AT5G47280 |
| 19204299 | C | G | 41545 | 5 | ns | ns | 3.30 | 3.22 | 3.09 |  |
| 19245844 | T | A | 56100 | 4 | ns | ns | 1.96 | 2.56 | 2.70 |  |
| 19301944 | A | G | 55673 | 3 | ns | ns | 1.48 | 1.05 | 0.92 |  |
| 19357617 | T | C | 53698 | 3 | ns | ns | 1.53 | 2.23 | 1.66 |  |
| 19411315 | C | T | 60471 | 7 | ns | ns | 3.18 | 2.75 | 2.30 |  |
| 19471786 | G | A | 61884 | 1 | 0.021 | 0.308 | 0.44 | 2.94 | 1.98 |  |
| 19533670 | G | A | 53080 | 9 | ns | ns | 4.65 | 4.84 | 4.16 |  |
| 19586750 | A | C | 51498 | 1 | 0.049 | 0.371 | 0.53 | 2.88 | 1.83 |  |
| 19638248 | C | T | 75190 | 6 | ns | ns | 2.19 | 4.13 | 3.17 |  |
| 19713438 | A | C | 10846 | 0 | ns | ns | 0.00 | 31.64 | 51.43 | AT5G48620  (*RPP8L*) |
| 19724284 | T | C | 44510 | 0 | 0.0262 | 0.308 | 0.00 | 6.09 | 4.95 |  |
| 19768794 | G | A | 27319 | 1 | ns | ns | 1.00 | 1.78 | 2.10 | AT5G48770 AT5G48780 |
| 19796113 | G | A | 55791 | 5 | ns | ns | 2.46 | 4.88 | 3.72 |  |
| 19851904 | T | C | 73317 | 12 | ns | ns | 4.49 | 1.58 | 1.54 | AT5G49140  (*HRG9*) |
| 19925221 | A | G | 38015 | 2 | ns | ns | 1.44 | 2.30 | 1.95 |  |
| 19963236 | T | C | 42313 | 8 | ns | ns | 5.19 | 3.36 | 3.28 |  |
| 20005549 | A | G | 40305 | 3 | ns | ns | 2.04 | 2.47 | 2.21 |  |
| 20045854 | A | G | 38139 | 2 | ns | ns | 1.44 | 3.30 | 2.53 |  |
| 20083993 | T | A | 15704 | 1 | ns | ns | 1.75 | 1.75 | 2.26 |  |
| 20099697 | T | G | 86734 | 2 | 0.0085 | 0.308 | 0.63 | 2.13 | 2.77 |  |
| 20186431 | C | A | 64095 | 5 | ns | ns | 2.14 | 4.39 | 3.87 |  |
| 20250526 | INDEL | INDEL | - | 0 |  |  | 0 | 0 | 0 |  |
| Total |  |  | 4691706 | 714 |  |  | 4.18 | 4.41 | 4.53 |  |
