# Supplementary material for: Recombination Rate Heterogeneity within Arabidopsis Disease Resistance Genes
Source: PLoS Genet. 2016 Jul 14;12(7):e1006179. doi: 10.1371/journal.pgen.1006179 (PMC4945094; doi:10.1371/journal.pgen.1006179)
Supplement: S4 Table — The ‘Genotyping Assay’ column indicates whether a given marker coordinate was genotyped by KBiosciences (SNP), or via dCAPs assays. (DOCX) [file pgen.1006179.s010.docx]

**S4 Table. Fine-mapping crossovers within the *HRG1* *MRC1* map interval using dCAPs genotyping.**

| Genotyping  Assay | Chr1 coordinate (bp) | Crossovers | Interval size (bp) | cM | cM/Mb |
| --- | --- | --- | --- | --- | --- |
| SNP | 24491863 | 1 | 2521 | 0.0315 | 12.48 |
| dCAPs | 24494384 | 1 | 835 | 0.0315 | 37.67 |
| dCAPs | 24495219 | 0 | 160 | 0 | 0 |
| dCAPs | 24495379 | 0 | 322 | 0 | 0 |
| dCAPs | 24495701 | 0 | 143 | 0 | 0 |
| dCAPs | 24495844 | 3 | 807 | 0.0944 | 116.93 |
| dCAPs | 24496651 | 0 | 200 | 0 | 0 |
| dCAPs | 24496851 | 1 | 500 | 0.0315 | 62.91 |
| dCAPs | 24497351 | 4 | 688 | 0.1258 | 182.87 |
| dCAPs | 24498039 | 0 | 272 | 0 | 0 |
| dCAPs | 24498311 | 1 | 303 | 0.0315 | 103.81 |
| dCAPs | 24498614 | 4 | 1636 | 0.1258 | 76.90 |
| dCAPs | 24500250 | 3 | 2877 | 0.0944 | 32.80 |
| dCAPs | 24503127 | 0 | 419 | 0 | 0 |
| SNP | 24503546 | 0 | 0 | 0 | 0 |
