# Supplementary material for: Recombination Rate Heterogeneity within Arabidopsis Disease Resistance Genes
Source: PLoS Genet. 2016 Jul 14;12(7):e1006179. doi: 10.1371/journal.pgen.1006179 (PMC4945094; doi:10.1371/journal.pgen.1006179)
Supplement: S5 Table — The ‘Genotyping Assay’ column indicates whether a given marker coordinate was genotyped by KBiosciences (SNP), or via dCAPs assays. (DOCX) [file pgen.1006179.s011.docx]

**S5 Table. Fine-mapping crossovers within the *HRG2 HRG3* *MRC5* map interval using dCAPs genotyping.**

| Genotyping  Assay | Chr5 coordinate (bp) | Crossovers | Interval size (bp) | cM | cM/Mb |
| --- | --- | --- | --- | --- | --- |
| SNP | 18755113 | 0 | 3963 | 0 | 0 |
| dCAPs | 18759076 | 0 | 1196 | 0 | 0 |
| dCAPs | 18760272 | 1 | 1025 | 0.0275 | 26.78 |
| dCAPs | 18761297 | 3 | 2207 | 0.0824 | 37.32 |
| dCAPs | 18763504 | 2 | 990 | 0.0549 | 55.46 |
| dCAPs | 18764494 | 2 | 1689 | 0.0549 | 32.51 |
| dCAPs | 18766183 | 3 | 1219 | 0.0824 | 67.56 |
| dCAPs | 18767402 | 0 | 2018 | 0 | 0 |
| dCAPs | 18769420 | 0 | 841 | 0 | 0 |
| SNP | 18770261 | 0 | 0 | 0 | 0 |
