# Supplementary material for: Recombination Rate Heterogeneity within Arabidopsis Disease Resistance Genes
Source: PLoS Genet. 2016 Jul 14;12(7):e1006179. doi: 10.1371/journal.pgen.1006179 (PMC4945094; doi:10.1371/journal.pgen.1006179)
Supplement: S6 Table — The ‘Genotyping Assay’ column indicates whether a given marker coordinate was genotyped by KBiosciences (SNP), or via dCAPs assays. (DOCX) [file pgen.1006179.s012.docx]

**S6 Table. Fine-mapping crossovers within the *WRR4* *MRC1* map interval using dCAPs genotyping.**

| Genotyping  Assay | Chr1 coordinate (bp) | Crossovers | Interval size (bp) | cM | cM/Mb |
| --- | --- | --- | --- | --- | --- |
| SNP | 21164727 | 0 | 2539 | 0 | 0 |
| dCAPs | 21167266 | 1 | 3795 | 0.0314 | 8.29 |
| dCAPs | 21171061 | 2 | 1374 | 0.0629 | 45.78 |
| dCAPs | 21172435 | 3 | 2386 | 0.0944 | 39.55 |
| dCAPs | 21174821 | 5 | 2039 | 0.157 | 77.13 |
| dCAPs | 21176860 | 0 | 2384 | 0 | 0 |
| dCAPs | 21179244 | 0 | 2236 | 0 | 0 |
| dCAPs | 21181480 | 1 | 1440 | 0.0315 | 21.84 |
| dCAPs | 21182920 | 1 | 2765 | 0.0315 | 11.38 |
| dCAPs | 21185685 | 1 | 18043 | 0.0315 | 1.74 |
| SNP | 21203728 | 0 | 0 | 0 | 0 |
