# Supplementary material for: Recombination Rate Heterogeneity within Arabidopsis Disease Resistance Genes
Source: PLoS Genet. 2016 Jul 14;12(7):e1006179. doi: 10.1371/journal.pgen.1006179 (PMC4945094; doi:10.1371/journal.pgen.1006179)
Supplement: S7 Table — The ‘Genotyping Assay’ column indicates whether a given marker coordinate was genotyped by KBiosciences (SNP), or via dCAPs assays. (DOCX) [file pgen.1006179.s013.docx]

**S7 Table. Fine-mapping crossovers within the *CW9* *MRC1* map interval using dCAPs genotyping.**

| Genotyping  Assay | Chr1 coordinate (bp) | Crossovers | Interval size (bp) | cM | cM/Mb |
| --- | --- | --- | --- | --- | --- |
| SNP | 21901949 | 0 | 966 | 0 | 0 |
| dCAPs | 21902915 | 2 | 1282 | 0.0629 | 49.07 |
| dCAPs | 21904197 | 0 | 467 | 0 | 0 |
| dCAPs | 21904664 | 1 | 635 | 0.0315 | 49.53 |
| dCAPs | 21905299 | 0 | 241 | 0 | 0 |
| dCAPs | 21905540 | 3 | 12325 | 0.0944 | 7.66 |
| SNP | 21917865 | 0 | 0 | 0 | 0 |
