# Supplementary material for: Recombination Rate Heterogeneity within Arabidopsis Disease Resistance Genes
Source: PLoS Genet. 2016 Jul 14;12(7):e1006179. doi: 10.1371/journal.pgen.1006179 (PMC4945094; doi:10.1371/journal.pgen.1006179)
Supplement: S8 Table — The ‘Genotyping Assay’ column indicates whether a given marker coordinate was genotyped by KBiosciences (SNP), or via dCAPs assays. (DOCX) [file pgen.1006179.s014.docx]

**S8 Table. Fine-mapping crossovers within the *HRG4-HRG5* *MRC1* map interval using dCAPs genotyping.**

| Genotyping  Assay | Chr1 coordinate (bp) | Crossovers | Interval size (bp) | cM | cM/Mb |
| --- | --- | --- | --- | --- | --- |
| SNP | 23460129 | 12 | 34305 | 0.3774 | 11 |
| dCAPs | 23494434 | 2 | 1063 | 0.0629 | 59.18 |
| dCAPs | 23495497 | 0 | 1627 | 0 | 0 |
| dCAPs | 23497124 | 1 | 2226 | 0.0315 | 14.13 |
| dCAPs | 23499350 | 4 | 751 | 0.1258 | 167.53 |
| dCAPs | 23500101 | 0 | 1163 | 0 | 0 |
| dCAPs | 23501264 | 1 | 3027 | 0.0315 | 10.39 |
| dCAPs | 23504291 | 3 | 9651 | 0.0944 | 9.78 |
| SNP | 23513942 | 0 | 0 | 0 | 0 |
