# Supplementary material for: Recombination Rate Heterogeneity within Arabidopsis Disease Resistance Genes
Source: PLoS Genet. 2016 Jul 14;12(7):e1006179. doi: 10.1371/journal.pgen.1006179 (PMC4945094; doi:10.1371/journal.pgen.1006179)
Supplement: S9 Table — The ‘Genotyping Assay’ column indicates whether a given marker coordinate was genotyped by KBiosciences (SNP), or via dCAPs assays. (DOCX) [file pgen.1006179.s015.docx]

**S9 Table. Fine-mapping crossovers within the *HRG6* *MRC1* map interval using dCAPs genotyping.** The ‘Genotyping Assay’ column indicates whether a given marker coordinate was genotyped by KBiosciences (SNP), or via dCAPs assays.

| Genotyping  Assay | Chr1 coordinate (bp) | Crossovers | Interval size (bp) | cM | cM/Mb |
| --- | --- | --- | --- | --- | --- |
| SNP | 23634704 | 3 | 5864 | 0.094 | 16.09 |
| dCAPs | 23640568 | 0 | 1805 | 0 | 0 |
| dCAPs | 23642373 | 0 | 6346 | 0 | 0 |
| dCAPs | 23648719 | 0 | 2161 | 0 | 0 |
| dCAPs | 23650880 | 2 | 1645 | 0.0629 | 38.24 |
| dCAPs | 23652525 | 1 | 659 | 0.0315 | 47.73 |
| dCAPs | 23653184 | 2 | 2599 | 0.0629 | 24.20 |
| dCAPs | 23655783 | 0 | 4155 | 0 | 0 |
| SNP | 23659938 | 0 | 0 | 0 | 0 |
