# Supplementary material for: Recombination Rate Heterogeneity within Arabidopsis Disease Resistance Genes
Source: PLoS Genet. 2016 Jul 14;12(7):e1006179. doi: 10.1371/journal.pgen.1006179 (PMC4945094; doi:10.1371/journal.pgen.1006179)
Supplement: S10 Table — The ‘Genotyping Assay’ column indicates whether a given marker coordinate was genotyped by KBiosciences (SNP), or via dCAPs assays. (DOCX) [file pgen.1006179.s016.docx]

**S10 Table. Fine-mapping crossovers within the *HRG7-HRG8* *MRC5* map interval using dCAPs genotyping.**

| Genotyping  Assay | Chr5 coordinate (bp) | Crossovers | Interval size (bp) | cM | cM/Mb |
| --- | --- | --- | --- | --- | --- |
| SNP | 18277632 | 1 | 6872 | 0.0275 | 3.99 |
| dCAPs | 18284504 | 1 | 1832 | 0.0275 | 14.98 |
| dCAPs | 18286336 | 0 | 1166 | 0 | 0 |
| dCAPs | 18287502 | 0 | 2314 | 0 | 0 |
| dCAPs | 18289816 | 1 | 690 | 0.0275 | 39.79 |
| dCAPs | 18290506 | 1 | 4094 | 0.0275 | 6.71 |
| dCAPs | 18294600 | 2 | 980 | 0.0549 | 56.02 |
| dCAPs | 18295580 | 2 | 2058 | 0.0549 | 26.68 |
| dCAPs | 18297638 | 2 | 1743 | 0.0549 | 31.50 |
| dCAPs | 18299381 | 0 | 20987 | 0 | 0 |
| dCAPs | 18320368 | 0 | 6218 | 0 | 0 |
| dCAPs | 18326586 | 0 | 3873 | 0 | 0 |
| dCAPs | 18330459 | 0 | 6006 | 0 | 0 |
| SNP | 18336465 | 0 | 0 | 0 | 0 |
