# Supplementary material for: Recombination Rate Heterogeneity within Arabidopsis Disease Resistance Genes
Source: PLoS Genet. 2016 Jul 14;12(7):e1006179. doi: 10.1371/journal.pgen.1006179 (PMC4945094; doi:10.1371/journal.pgen.1006179)
Supplement: S11 Table — The ‘Genotyping Assay’ column indicates whether a given marker coordinate was genotyped by KBiosciences (SNP), or via dCAPs assays. (DOCX) [file pgen.1006179.s017.docx]

**S11 Table. Fine-mapping crossovers within the *HRG9* *MRC5* map interval using dCAPs genotyping.**

| Genotyping  Assay | Chr5 coordinate (bp) | Crossovers | Interval size (bp) | cM | cM/Mb |
| --- | --- | --- | --- | --- | --- |
| SNP | 19851904 | 5 | 64925 | 0.1373 | 2.11 |
| dCAPs | 19916829 | 0 | 2317 | 0 | 0 |
| dCAPs | 19919146 | 1 | 1902 | 0.0275 | 14.43 |
| dCAPs | 19921048 | 0 | 1382 | 0 | 0 |
| dCAPs | 19922430 | 6 | 1456 | 0.1647 | 113.13 |
| dCAPs | 19923886 | 0 | 302 | 0 | 0 |
| dCAPs | 19924188 | 0 | 1033 | 0 | 0 |
| SNP | 19925221 | 0 | 0 | 0 | 0 |
