# Supplementary material for: Recombination Rate Heterogeneity within Arabidopsis Disease Resistance Genes
Source: PLoS Genet. 2016 Jul 14;12(7):e1006179. doi: 10.1371/journal.pgen.1006179 (PMC4945094; doi:10.1371/journal.pgen.1006179)
Supplement: S12 Table — The mean and maximum crossover frequency (cM/Mb) measured at known plant hotspots is listed, together with the species studied, hotspot name, width of measured region, genetic distance (cM) and crossover frequency (cM/Mb), together with chromosome average recombination rates. (DOCX) [file pgen.1006179.s018.docx]

**S12 Table. Recombination rates of plant crossover hotspots.**

| Species | Hotspot | Hotspot width (bp) | cM | Mean cM/Mb | Peak cM/Mb | Location | Average cM/Mb | Reference |
| --- | --- | --- | --- | --- | --- | --- | --- | --- |
| *Arabidopsis thaliana* | *3a* | 5,825 | 0.21 | 36.22 | 93.06 | Genic and Intergenic | 4.82 | Choi et al., 2013; Yelina et al., 2012 |
| *Arabidopsis thaliana* | *3b* | 5,746 | 0.11 | 20.01 | 68.81 | Intergenic | 4.82 | Choi et al., 2013 |
| *Arabidopsis thaliana* | *14a* | 7,283 | 0.55 | 75.52 | 261 | Genic and Intergenic | 4.82 | Drouaud et al., 2013 |
| *Arabidopsis thaliana* | *130x* | 12,488 | 0.53 | 42.44 | 167 | Intergenic | 4.82 | Drouaud et al., 2013 |
| *Arabidopsis thaliana* | *RAC1* | 5,301 | 0.16 | 24.34 | 117 | Genic | 4.82 | This study |
| *Arabidopsis thaliana* | *HRG1* | 4,230 | 0.44 | 104.11 | 182.87 | Genic | 4.82 | This study |
| *Arabidopsis thaliana* | *HRG2 HRG3* | 10,344 | 0.3 | 29.19 | 67.56 | Genic | 4.82 | This study |
| *Arabidopsis thaliana* | *WRR4* | 18,419 | 0.44 | 23.91 | 77.13 | Genic and intergenic | 4.82 | This study |
| *Arabidopsis thaliana* | *CW9* | 3,591 | 0.19 | 11.86 | 49.53 | Genic and intergenic | 4.82 | This study |
| *Arabidopsis thaliana* | *HRG4 HRG5* | 9,857 | 0.72 | 73.39 | 167.53 | Genic and intergenic | 4.82 | This study |
| *Arabidopsis thaliana* | *HRG6* | 7,064 | 0.16 | 22.26 | 47.73 | Genic | 4.82 | This study |
| *Arabidopsis thaliana* | *HRG7 HRG8* | 20,006 | 0.27 | 13.72 | 56.02 | Genic and intergenic | 4.82 | This study |
| *Arabidopsis thaliana* | *HRG9* | 7,057 | 0.19 | 27.23 | 113.13 | Genic | 4.82 | This study |
| *Zea mays* | *a1* | 377 | 0.00607 | 16.12 | 16.12 | Genic | 2.12 | Brown and Sundaresan, 1991 |
| *Zea mays* | *Yz1* | 3,400 | 0.028 | 8.2 | 13 | Genic | 2.12 | Yao & Schnable, 2005 |
| *Zea mays* | *Bronze* | 9,100 | 0.11 | 12.09 | 12.09 | Genic | 2.12 | Dooner & Martinez-Ferez, 1997 |
| *Triticum aestivum* | *HGA3* | 23,000 | 0.2 | 8.82 | 8.82 | Genic | 0.85 | Saintenace et al., 2011 |

[1–7]**.**
