# Supplementary material for: Recombination Rate Heterogeneity within Arabidopsis Disease Resistance Genes
Source: PLoS Genet. 2016 Jul 14;12(7):e1006179. doi: 10.1371/journal.pgen.1006179 (PMC4945094; doi:10.1371/journal.pgen.1006179)
Supplement: S13 Table — 192 Col×Ler F2 individuals were analysed by genotyping-by-sequencing. The number of crossovers (CO) per F2 and in total are listed, for the whole genome and for each chromosome separately. (DOCX) [file pgen.1006179.s019.docx]

**S13 Table. Crossovers identified by genotyping by sequencing in a Col×Ler F_2_ population.**

|  | Col/Ler | |
| --- | --- | --- |
|  | CO/F_2_ | Total CO |
| Total | 6.41 | 1,230 |
| Chr1 | 1.64 | 315 |
| Chr2 | 1.09 | 210 |
| Chr3 | 1.17 | 225 |
| Chr4 | 1.03 | 198 |
| Chr5 | 1.47 | 282 |
