# Supplementary material for: Recombination Rate Heterogeneity within Arabidopsis Disease Resistance Genes
Source: PLoS Genet. 2016 Jul 14;12(7):e1006179. doi: 10.1371/journal.pgen.1006179 (PMC4945094; doi:10.1371/journal.pgen.1006179)
Supplement: S14 Table — A 9.419 kb amplicon (Chr1: 11,288,146–11,297,565 bp) containing the RAC1 gene was amplified using allele-specific oligonucleotides to estimate the number of parental (non-recombinant) and crossover molecules per μl of Col/Ler F1 pollen genomic DNA. Genetic distance was calculated as described [83]. (DOCX) [file pgen.1006179.s020.docx]

**S14 Table. Measurement of *RAC1* recombination rate via pollen-typing.**

| Parentals/μl | 3,008.99 |
| --- | --- |
| Crossovers/μl | 6.2 |
| cM | 0.16 |
| cM St.Dev. | 0.03 |
| cM/Mb | 16.99 |
| cM/Mb St.Dev. | 3.19 |
