# Supplementary material for: Recombination Rate Heterogeneity within Arabidopsis Disease Resistance Genes
Source: PLoS Genet. 2016 Jul 14;12(7):e1006179. doi: 10.1371/journal.pgen.1006179 (PMC4945094; doi:10.1371/journal.pgen.1006179)
Supplement: S15 Table — 181 single RAC1 crossover molecules were Sanger sequenced to identify recombination sites to the resolution of single polymorphisms. Col and Ler genotypes and number of crossovers per interval are listed, together with cM/Mb. (DOCX) [file pgen.1006179.s021.docx]

**S15 Table. Crossover distributions across the *RAC1* *R* gene hotspot analysed via pollen-typing.**

| Chr1 coordinate (TAIR10) | Col | Ler | Interval length (bp) | Crossovers | cM/Mb |
| --- | --- | --- | --- | --- | --- |
| 11288165 | A | T | 16 | 0 | 0 |
| 11288181 | A | G | 11 | 0 | 0 |
| 11288192 | T | A | 7 | 0 | 0 |
| 11288199 | T | C | 9 | 0 | 0 |
| 11288208 | - | CTCTCTACATTACCATCTTCAG | 33 | 0 | 0 |
| 11288241 | C | T | 1 | 0 | 0 |
| 11288242 | C | T | 9 | 0 | 0 |
| 11288251 | A | - | 1 | 0 | 0 |
| 11288252 | A | G | 12 | 0 | 0 |
| 11288264 | - | TT | 67 | 0 | 0 |
| 11288331 | C | A | 127 | 0 | 0 |
| 11288458 | A | T | 108 | 0 | 0 |
| 11288566 | A | C | 19 | 0 | 0 |
| 11288585 | C | A | 77 | 0 | 0 |
| 11288662 | C | T | 76 | 0 | 0 |
| 11288738 | C | C | 62 | 0 | 0 |
| 11288800 | GACTGTGA | - | 72 | 3 | 36.83 |
| 11288872 | A | G | 9 | 0 | 0 |
| 11288881 | G | C | 17 | 0 | 0 |
| 11288898 | G | T | 23 | 1 | 38.43 |
| 11288921 | C | G | 8 | 0 | 0 |
| 11288929 | T | C | 1 | 0 | 0 |
| 11288930 | G | A | 19 | 0 | 0 |
| 11288949 | G | T | 148 | 4 | 23.89 |
| 11289097 | T | C | 178 | 4 | 19.86 |
| 11289275 | T | C | 118 | 1 | 7.49 |
| 11289393 | G | A | 53 | 0 | 0 |
| 11289446 | T | A | 5 | 0 | 0 |
| 11289451 | A | G | 103 | 3 | 25.75 |
| 11289554 | T | C | 130 | 1 | 6.80 |
| 11289684 | A | C | 99 | 4 | 35.72 |
| 11289783 | CTA | - | 78 | 2 | 22.67 |
| 11289861 | T | G | 80 | 0 | 0 |
| 11289941 | T | C | 75 | 2 | 23.57 |
| 11290016 | A | T | 52 | 2 | 34 |
| 11290068 | C | T | 69 | 0 | 0 |
| 11290137 | C | T | 22 | 0 | 0 |
| 11290159 | C | A | 28 | 0 | 0 |
| 11290187 | C | T | 10 | 0 | 0 |
| 11290197 | T | C | 10 | 0 | 0 |
| 11290207 | C | G | 186 | 3 | 14.26 |
| 11290393 | G | T | 2 | 0 | 0 |
| 11290395 | T | G | 1 | 0 | 0 |
| 11290396 | T | G | 2 | 0 | 0 |
| 11290398 | T | C | 1 | 0 | 0 |
| 11290399 | T | A | 5 | 0 | 0 |
| 11290404 | T | A | 6 | 0 | 0 |
| 11290410 | C | G | 33 | 0 | 0 |
| 11290443 | T | G | 21 | 0 | 0 |
| 11290464 | G | A | 121 | 0 | 0 |
| 11290585 | T | - | 67 | 1 | 13.19 |
| 11290652 | G | A | 2 | 0 | 0 |
| 11290654 | T | C | 8 | 0 | 0 |
| 11290662 | G | T | 2 | 0 | 0 |
| 11290664 | - | ATGTATTATGGGTAAATCTAAACAAGAATAAA | 23 | 0 | 0 |
| 11290687 | A | C | 26 | 0 | 0 |
| 11290713 | A | - | 27 | 0 | 0 |
| 11290740 | C | T | 13 | 0 | 0 |
| 11290753 | C | G | 29 | 0 | 0 |
| 11290782 | G | A | 95 | 0 | 0 |
| 11290877 | G | C | 6 | 0 | 0 |
| 11290883 | T | A | 6 | 0 | 0 |
| 11290889 | TTA | - | 10 | 0 | 0 |
| 11290899 | T | A | 67 | 0 | 0 |
| 11290966 | T | A | 13 | 0 | 0 |
| 11290979 | T | A | 31 | 0 | 0 |
| 11291010 | C | T | 1 | 0 | 0 |
| 11291011 | - | ACAAGTGTACAAAGTTT | 21 | 0 | 0 |
| 11291032 | T | C | 4 | 0 | 0 |
| 11291036 | A | T | 20 | 0 | 0 |
| 11291056 | G | A | 105 | 0 | 0 |
| 11291161 | C | G | 19 | 0 | 0 |
| 11291180 | G | A | 83 | 0 | 0 |
| 11291263 | G | A | 9 | 0 | 0 |
| 11291272 | T | C | 62 | 0 | 0 |
| 11291334 | A | C | 7 | 0 | 0 |
| 11291341 | C | A | 13 | 0 | 0 |
| 11291354 | T | C | 36 | 0 | 0 |
| 11291390 | C | T | 6 | 0 | 0 |
| 11291396 | C | G | 2 | 0 | 0 |
| 11291398 | C | G | 5 | 0 | 0 |
| 11291403 | A | T | 4 | 0 | 0 |
| 11291407 | T | C | 13 | 0 | 0 |
| 11291420 | C | T | 15 | 0 | 0 |
| 11291435 | T | C | 2 | 0 | 0 |
| 11291437 | C | T | 1 | 0 | 0 |
| 11291438 | A | G | 28 | 2 | 63.14 |
| 11291466 | G | A | 9 | 0 | 0 |
| 11291475 | T | C | 1 | 0 | 0 |
| 11291476 | G | C | 54 | 0 | 0 |
| 11291530 | T | C | 2 | 0 | 0 |
| 11291532 | G | T | 8 | 0 | 0 |
| 11291540 | A | C | 17 | 0 | 0 |
| 11291557 | G | A | 23 | 0 | 0 |
| 11291580 | C | T | 8 | 0 | 0 |
| 11291588 | C | T | 2 | 0 | 0 |
| 11291590 | AAC | - | 28 | 0 | 0 |
| 11291618 | G | A | 3 | 0 | 0 |
| 11291621 | ACTCCTTCC | T | 29 | 0 | 0 |
| 11291650 | T | A | 4 | 0 | 0 |
| 11291654 | G | T | 4 | 0 | 0 |
| 11291658 | T | A | 32 | 0 | 0 |
| 11291690 | C | T | 72 | 0 | 0 |
| 11291762 | C | T | 17 | 0 | 0 |
| 11291779 | A | T | 9 | 0 | 0 |
| 11291788 | T | A | 4 | 0 | 0 |
| 11291792 | A | C | 4 | 0 | 0 |
| 11291796 | CAAAGT | - | 22 | 0 | 0 |
| 11291818 | A | G | 50 | 0 | 0 |
| 11291868 | G | C | 11 | 0 | 0 |
| 11291879 | A | G | 38 | 0 | 0 |
| 11291917 | A | G | 13 | 0 | 0 |
| 11291930 | - | TA | 2 | 0 | 0 |
| 11291932 | - | TA | 8 | 0 | 0 |
| 11291940 | T | G | 10 | 0 | 0 |
| 11291950 | C | A | 12 | 0 | 0 |
| 11291962 | C | T | 7 | 0 | 0 |
| 11291969 | C | T | 2 | 0 | 0 |
| 11291971 | G | T | 1 | 0 | 0 |
| 11291972 | A | G | 3 | 0 | 0 |
| 11291975 | G | A | 15 | 0 | 0 |
| 11291990 | TATA | - | 5 | 0 | 0 |
| 11291995 | G | A | 18 | 0 | 0 |
| 11292013 | G | T | 17 | 0 | 0 |
| 11292030 | T | A | 1 | 0 | 0 |
| 11292031 | T | A | 1 | 0 | 0 |
| 11292032 | T | A | 26 | 0 | 0 |
| 11292058 | T | C | 27 | 1 | 32.74 |
| 11292085 | G | A | 130 | 10 | 68 |
| 11292215 | T | C | 53 | 0 | 0 |
| 11292268 | C | T | 111 | 2 | 15.93 |
| 11292379 | T | C | 89 | 3 | 29.80 |
| 11292468 | T | C | 116 | 1 | 7.62 |
| 11292584 | T | G | 21 | 2 | 84.19 |
| 11292605 | T | C | 10 | 0 | 0 |
| 11292615 | A | G | 148 | 14 | 83.62 |
| 11292763 | A | G | 68 | 8 | 104 |
| 11292831 | T | C | 55 | 6 | 96.43 |
| 11292886 | A | T | 28 | 1 | 31.57 |
| 11292914 | G | A | 69 | 3 | 38.43 |
| 11292983 | G | T | 32 | 2 | 55.25 |
| 11293015 | T | C | 73 | 5 | 60.55 |
| 11293088 | G | A | 136 | 18 | 117 |
| 11293224 | - | AA | 255 | 20 | 69.33 |
| 11293479 | T | G | 210 | 17 | 71.56 |
| 11293689 | C | A | 9 | 0 | 0 |
| 11293698 | - | AG | 15 | 0 | 0 |
| 11293713 | G | T | 29 | 0 | 0 |
| 11293742 | A | T | 56 | 0 | 0 |
| 11293798 | A | C | 133 | 4 | 26.59 |
| 11293931 | C | - | 49 | 0 | 0 |
| 11293980 | G | T | 9 | 0 | 0 |
| 11293989 | T | C | 1 | 0 | 0 |
| 11293990 | T | G | 17 | 0 | 0 |
| 11294007 | A | C | 36 | 0 | 0 |
| 11294043 | T | A | 15 | 0 | 0 |
| 11294058 | T | C | 36 | 3 | 73.66 |
| 11294094 | C | A | 14 | 2 | 126.28 |
| 11294108 | C | T | 20 | 1 | 44.20 |
| 11294128 | - | AA | 11 | 0 | 0 |
| 11294139 | C | T | 2 | 0 | 0 |
| 11294141 | C | T | 37 | 0 | 0 |
| 11294178 | A | C | 6 | 0 | 0 |
| 11294184 | G | C | 26 | 0 | 0 |
| 11294210 | A | C | 7 | 0 | 0 |
| 11294217 | T | A | 119 | 0 | 0 |
| 11294336 | T | A | 13 | 0 | 0 |
| 11294349 | A | T | 2 | 0 | 0 |
| 11294351 | T | G | 8 | 0 | 0 |
| 11294359 | A | T | 8 | 0 | 0 |
| 11294367 | A | T | 1 | 0 | 0 |
| 11294368 | G | A | 16 | 0 | 0 |
| 11294384 | A | T | 3 | 0 | 0 |
| 11294387 | C | G | 5 | 0 | 0 |
| 11294392 | C | T | 4 | 0 | 0 |
| 11294396 | A | C | 48 | 0 | 0 |
| 11294444 | G | C | 25 | 0 | 0 |
| 11294469 | A | G | 21 | 0 | 0 |
| 11294490 | A | C | 9 | 0 | 0 |
| 11294499 | A | C | 1 | 0 | 0 |
| 11294500 | T | A | 6 | 0 | 0 |
| 11294506 | - | T | 19 | 0 | 0 |
| 11294525 | C | A | 4 | 0 | 0 |
| 11294529 | T | A | 9 | 0 | 0 |
| 11294538 | - | G | 1 | 0 | 0 |
| 11294539 | A | T | 11 | 0 | 0 |
| 11294550 | A | T | 21 | 0 | 0 |
| 11294571 | T | C | 2 | 0 | 0 |
| 11294573 | AAAAAGGTGAGAGCTTAAAAACCCAC | - | 52 | 0 | 0 |
| 11294625 | T | - | 28 | 0 | 0 |
| 11294653 | A | C | 7 | 0 | 0 |
| 11294660 | A | T | 9 | 0 | 0 |
| 11294669 | A | - | 2 | 0 | 0 |
| 11294671 | C | T | 11 | 0 | 0 |
| 11294682 | C | T | 6 | 0 | 0 |
| 11294688 | T | A | 6 | 0 | 0 |
| 11294694 | G | C | 21 | 0 | 0 |
| 11294715 | TG | - | 27 | 0 | 0 |
| 11294742 | C | A | 17 | 0 | 0 |
| 11294759 | A | T | 15 | 0 | 0 |
| 11294774 | - | GC | 3 | 0 | 0 |
| 11294777 | A | - | 26 | 0 | 0 |
| 11294803 | - | A | 10 | 0 | 0 |
| 11294813 | T | C | 23 | 0 | 0 |
| 11294836 | - | TGT | 38 | 0 | 0 |
| 11294874 | C | A | 31 | 0 | 0 |
| 11294905 | A | G | 11 | 0 | 0 |
| 11294916 | A | C | 19 | 0 | 0 |
| 11294935 | G | A | 2 | 0 | 0 |
| 11294937 | T | A | 23 | 0 | 0 |
| 11294960 | C | A | 20 | 0 | 0 |
| 11294980 | T | A | 11 | 0 | 0 |
| 11294991 | A | G | 47 | 0 | 0 |
| 11295038 | G | T | 53 | 0 | 0 |
| 11295091 | C | A | 35 | 0 | 0 |
| 11295126 | C | A | 25 | 0 | 0 |
| 11295151 | G | A | 17 | 0 | 0 |
| 11295168 | A | T | 4 | 0 | 0 |
| 11295172 | T | C | 1 | 0 | 0 |
| 11295173 | T | C | 9 | 0 | 0 |
| 11295182 | G | A | 5 | 0 | 0 |
| 11295187 | C | T | 6 | 0 | 0 |
| 11295193 | C | A | 2 | 0 | 0 |
| 11295195 | C | G | 22 | 0 | 0 |
| 11295217 | T | C | 17 | 0 | 0 |
| 11295234 | A | T | 28 | 0 | 0 |
| 11295262 | C | A | 11 | 0 | 0 |
| 11295273 | T | G | 16 | 0 | 0 |
| 11295289 | G | A | 1 | 0 | 0 |
| 11295290 | G | A | 9 | 0 | 0 |
| 11295299 | C | T | 52 | 0 | 0 |
| 11295351 | CT | - | 7 | 0 | 0 |
| 11295358 | - | T | 6 | 0 | 0 |
| 11295364 | C | T | 11 | 0 | 0 |
| 11295375 | A | G | 3 | 0 | 0 |
| 11295378 | C | G | 1 | 0 | 0 |
| 11295379 | C | T | 9 | 0 | 0 |
| 11295388 | A | T | 18 | 0 | 0 |
| 11295406 | T | A | 37 | 0 | 0 |
| 11295443 | A | T | 36 | 0 | 0 |
| 11295479 | T | A | 17 | 1 | 52 |
| 11295496 | G | T | 28 | 0 | 0 |
| 11295524 | T | A | 17 | 0 | 0 |
| 11295541 | G | - | 3 | 0 | 0 |
| 11295544 | T | C | 16 | 0 | 0 |
| 11295560 | T | A | 5 | 0 | 0 |
| 11295565 | G | A | 3 | 0 | 0 |
| 11295568 | T | A | 33 | 0 | 0 |
| 11295601 | T | A | 8 | 0 | 0 |
| 11295609 | A | T | 127 | 1 | 6.96 |
| 11295736 | C | G | 154 | 3 | 17.22 |
| 11295890 | T | G | 31 | 0 | 0 |
| 11295921 | G | A | 5 | 0 | 0 |
| 11295926 | - | TAG | 27 | 0 | 0 |
| 11295953 | C | A | 41 | 0 | 0 |
| 11295994 | A | G | 79 | 0 | 0 |
| 11296073 | C | T | 57 | 0 | 0 |
| 11296130 | C | T | 96 | 5 | 46.04 |
| 11296226 | T | G | 24 | 0 | 0 |
| 11296250 | T | C | 19 | 0 | 0 |
| 11296269 | TGGA | - | 22 | 0 | 0 |
| 11296291 | A | C | 178 | 2 | 9.93 |
| 11296469 | T | C | 193 | 6 | 27.48 |
| 11296662 | A | T | 23 | 0 | 0 |
| 11296685 | T | C | 22 | 0 | 0 |
| 11296707 | C | T | 7 | 0 | 0 |
| 11296714 | A | C | 27 | 0 | 0 |
| 11296741 | T | C | 81 | 4 | 43.65 |
| 11296822 | C | A | 42 | 1 | 21.05 |
| 11296864 | T | A | 12 | 0 | 0 |
| 11296876 | T | C | 19 | 0 | 0 |
| 11296895 | TTGACATAAGAAACCTAAGAA | - | 40 | 0 | 0 |
| 11296935 | - | AAA | 12 | 0 | 0 |
| 11296947 | C | T | 2 | 0 | 0 |
| 11296949 | T | C | 5 | 0 | 0 |
| 11296954 | G | A | 8 | 0 | 0 |
| 11296962 | A | G | 2 | 0 | 0 |
| 11296964 | C | T | 2 | 0 | 0 |
| 11296966 | T | C | 3 | 0 | 0 |
| 11296969 | C | - | 4 | 0 | 0 |
| 11296973 | A | G | 6 | 0 | 0 |
| 11296979 | C | T | 6 | 0 | 0 |
| 11296985 | C | A | 13 | 0 | 0 |
| 11296998 | C | T | 134 | 0 | 0 |
| 11297132 | T | G | 80 | 0 | 0 |
| 11297212 | C | A | 6 | 0 | 0 |
| 11297218 | A | T | 1 | 0 | 0 |
| 11297219 | A | G | 28 | 0 | 0 |
| 11297247 | C | A | 41 | 0 | 0 |
| 11297288 | A | T | 8 | 2 | 220.99 |
| 11297296 | A | G | 68 | 0 | 0 |
| 11297364 | - | G | 45 | 0 | 0 |
| 11297409 | A | - | 3 | 0 | 0 |
| 11297412 | G | T | 32 | 0 | 0 |
| 11297444 | A | G | 10 | 0 | 0 |
| 11297454 | T | C | 7 | 0 | 0 |
| 11297461 | T | G | 31 | 0 | 0 |
| 11297492 | A | G | 56 | 0 | 0 |
| 11297548 | G | A | 0 | 0 | 0 |
|  |  | Total | 9,383 | 181 |  |
