# Supplementary material for: Recombination Rate Heterogeneity within Arabidopsis Disease Resistance Genes
Source: PLoS Genet. 2016 Jul 14;12(7):e1006179. doi: 10.1371/journal.pgen.1006179 (PMC4945094; doi:10.1371/journal.pgen.1006179)
Supplement: S16 Table — The number of read pairs surviving sequential analysis filters are listed in order to identify RAC1 crossover read pairs. Paired end reads (1 and 2) were separated and aligned to the Col or Ler RAC1 parental template sequences, allowing only exact matches (Mapped). Read pair ends that mapped to both Col and Ler were then excluded (Unique). Read pair ends (1 and 2) that mapped to Col and Ler were identified (Matched), where the Ler mapping read had a lower coordinate than the Col mapping read (Orientate), and that were on opposite strands (Strand). These filters yielded 182,909 crossover read pairs. (DOCX) [file pgen.1006179.s022.docx]

**S16 Table. Crossover distributions across the *RAC1* *R* gene hotspot analysed via pollen typing.**

| Total reads | Read | Parental  Template | Mapped | Unique | Match | Orientate | Strand | Total |
| --- | --- | --- | --- | --- | --- | --- | --- | --- |
| 21,517,099 | 1 | Col | 10,504,978 | 6,567,826 | 103,272 | 92,710 | 92,620 | 182,909 |
| 21,517,099 | 2 | Ler | 9,918,809 | 6,364,442 |  |  |  |  |
| 21,517,099 | 1 | Ler | 10,237,197 | 6,300,045 | 101,142 | 90,380 | 90,289 |  |
| 21,517,099 | 2 | Col | 9,942,487 | 6,388,120 |  |  |  |  |
