# Supplementary material for: Recombination Rate Heterogeneity within Arabidopsis Disease Resistance Genes
Source: PLoS Genet. 2016 Jul 14;12(7):e1006179. doi: 10.1371/journal.pgen.1006179 (PMC4945094; doi:10.1371/journal.pgen.1006179)
Supplement: S17 Table — NBS-LRR genes located within the MRC genetic maps were divided into two groups according to whether their interval had higher or lower crossover frequency (cM/Mb) compared with the male Col×Ler genome average (4.82 cM/Mb) [71]. We then matched the position weight matrix of a CTT-repeat motif previously identified as enriched at Arabidopsis historical crossover hotspots (CTTCTTCTTCTTCTTC) [37] to +1 kb windows around NBS-LRR gene transcriptional start sites (TSSs), allowing matches with >80% identity. The location, width and sequence of matching CTT motifs are listed. Motif coordinates are given relative to the 1 kb of sequence matched to. (DOCX) [file pgen.1006179.s023.docx]

**S17 Table. CTT-repeat motifs associated with high and low *MRC* recombination NBS-LRR genes.**

| *MRC* | cM/Mb | NBS-LRR gene | NBS-LRR start | NBS-LRR end | Strand | CTT start | CTT end | CTT width | CTT Sequence |
| --- | --- | --- | --- | --- | --- | --- | --- | --- | --- |
| *MRC1* | High | AT1G56510.1 | 21167589 | 21173632 | + | 617 | 638 | 22 | T G G C T T C T T C T T C T T C C T C A C C |
| *MRC1* | High | AT1G56520.2 | 21174664 | 21178974 | - | 556 | 577 | 22 | T G G C T T C T T C T T C T T C C T C A C C |
| *MRC1* | High | AT1G56540.1 | 21181664 | 21185306 | + | 502 | 529 | 28 | T G G C T T C T T C T T C T T C T T C T T C T T C A C G |
| *MRC1* | High | AT1G57650.1 | 21351291 | 21354311 | + | 0 | 0 | 0 |  |
| *MRC1* | High | AT1G57830.1 | 21420309 | 21420803 | + | 0 | 0 | 0 |  |
| *MRC1* | High | AT1G57850.1 | 21426690 | 21427349 | + | 0 | 0 | 0 |  |
| *MRC1* | High | AT1G59620.1 | 21902284 | 21905685 | + | 0 | 0 | 0 |  |
| *MRC1* | High | AT1G59780.1 | 21993581 | 21997691 | - | 0 | 0 | 0 |  |
| *MRC1* | High | AT1G61300.1 | 22607466 | 22610273 | - | 0 | 0 | 0 |  |
| *MRC1* | High | AT1G61310.1 | 22613063 | 22615943 | - | 84 | 105 | 22 | C A A T T T C T T C T T C C T C T T C C A T |
| *MRC1* | High | AT1G61310.1 | 22613063 | 22615943 | - | 387 | 405 | 19 | T C T C T T C T T C T T C T C A T T C |
| *MRC1* | High | AT1G62630.1 | 23185912 | 23188593 | + | 0 | 0 | 0 |  |
| *MRC1* | High | AT1G63350.1 | 23494935 | 23497631 | - | 0 | 0 | 0 |  |
| *MRC1* | High | AT1G63360.1 | 23499515 | 23502169 | - | 0 | 0 | 0 |  |
| *MRC1* | High | AT1G63730.1 | 23641770 | 23645132 | + | 502 | 523 | 22 | T G G C T T C C T C T T C T T C T T C G C C |
| *MRC1* | High | AT1G63740.1 | 23645429 | 23648973 | + | 472 | 493 | 22 | T G G C T T C C T C T T C T T C T T C G C C |
| *MRC1* | High | AT1G63750.3 | 23650848 | 23655359 | + | 609 | 627 | 19 | C T T C C C C T T C T T C T T C C A A |
| *MRC1* | High | AT1G63860.1 | 23701484 | 23706005 | - | 511 | 529 | 19 | T G G C T T C T T C T T C C T C T T G |
| *MRC1* | High | AT1G63870.1 | 23707131 | 23711901 | - | 517 | 532 | 16 | C T C C T T C C T C T A T T T C |
| *MRC1* | High | AT1G63880.1 | 23712276 | 23716124 | - | 579 | 603 | 25 | T G G C T T C T C C T T C T T C T T T T T C G T C |
| *MRC1* | High | AT1G64070.1 | 23779949 | 23783449 | + | 502 | 523 | 22 | T G G C T T C T T C C T C T T C T T C T G C |
| *MRC1* | High | AT1G65850.2 | 24494678 | 24498542 | + | 747 | 765 | 19 | C T T C T A C T A T A T C T T C T T C |
| *MRC1* | High | AT1G66090.1 | 24602146 | 24604763 | + | 580 | 634 | 55 | C T G C A T C T A C T T C T T C T T C T T C T T C A T C A T C A T C T T C T T C T T T A T C T T C T C C T T C |
| *MRC5* | High | AT5G40060.1 | 16034576 | 16039096 | + | 517 | 535 | 19 | C T A C T T C T T C C T C T T C T T G |
| *MRC5* | High | AT5G40090.1 | 16041919 | 16043494 | - | 502 | 535 | 34 | T G T C T T C T G C T T C T T C A T C T T C T G C T G C T T C T T T |
| *MRC5* | High | AT5G40100.1 | 16043929 | 16047355 | + | 0 | 0 | 0 |  |
| *MRC5* | High | AT5G41540.1 | 16612659 | 16616063 | - | 0 | 0 | 0 |  |
| *MRC5* | High | AT5G41550.1 | 16617232 | 16620785 | - | 170 | 194 | 25 | C T T C C T C T T C G T C T T C C T C T T T T T C |
| *MRC5* | High | AT5G41550.1 | 16617232 | 16620785 | - | 505 | 526 | 22 | C T T T G T C T T C T T C T T C G T C T A A |
| *MRC5* | High | AT5G44510.1 | 17929673 | 17934188 | - | 565 | 604 | 40 | C T T C T T C T C T A T C T T C T T C A T C T C C C C C T T C T T C T T T G T C |
| *MRC5* | High | AT5G44900.1 | 18135446 | 18136438 | + | 501 | 516 | 16 | C T T C T T G T T T T T C C T C |
| *MRC5* | High | AT5G44900.1 | 18135446 | 18136438 | + | 558 | 573 | 16 | C T C C T T C T C C C T C T T T |
| *MRC5* | High | AT5G44910.1 | 18137354 | 18138235 | + | 432 | 450 | 19 | C A G C T T C T T C T T C T C C A T A |
| *MRC5* | High | AT5G44920.1 | 18138775 | 18140190 | + | 0 | 0 | 0 |  |
| *MRC5* | High | AT5G45000.1 | 18165383 | 18167193 | + | 0 | 0 | 0 |  |
| *MRC5* | High | AT5G46260.1 | 18759102 | 18763358 | - | 502 | 523 | 22 | T G G C T T C T T C A T C T T C T T C T C G |
| *MRC5* | High | AT5G46270.1 | 18764349 | 18769090 | - | 751 | 772 | 22 | T G G C T T C T T C A T C T T C T T C T C G |
| *MRC1* | Low | AT1G57670.1 | 21357169 | 21359872 | - | 0 | 0 | 0 |  |
| *MRC1* | Low | AT1G58390.1 | 21690962 | 21694447 | - | 0 | 0 | 0 |  |
| *MRC1* | Low | AT1G58400.1 | 21696165 | 21699118 | - | 0 | 0 | 0 |  |
| *MRC1* | Low | AT1G58410.1 | 21701185 | 21704255 | - | 0 | 0 | 0 |  |
| *MRC1* | Low | AT1G58602.1 | 21746373 | 21764969 | + | 0 | 0 | 0 |  |
| *MRC1* | Low | AT1G58807.1 | 21780185 | 21784296 | + | 0 | 0 | 0 |  |
| *MRC1* | Low | AT1G58848.1 | 21791783 | 21797050 | + | 0 | 0 | 0 |  |
| *MRC1* | Low | AT1G59124.1 | 21816443 | 21820572 | + | 0 | 0 | 0 |  |
| *MRC1* | Low | AT1G59218.1 | 21828033 | 21832708 | + | 0 | 0 | 0 |  |
| *MRC1* | Low | AT1G60320.1 | 22235091 | 22235606 | + | 0 | 0 | 0 |  |
| *MRC1* | Low | AT1G61105.1 | 22513585 | 22514492 | - | 0 | 0 | 0 |  |
| *MRC1* | Low | AT1G61180.2 | 22551345 | 22554649 | + | 528 | 546 | 19 | T C T C T T C T T C T T C T C A T T C |
| *MRC1* | Low | AT1G61190.1 | 22557602 | 22560687 | + | 0 | 0 | 0 |  |
| *MRC1* | Low | AT1G65390.1 | 24292478 | 24294631 | + | 599 | 614 | 16 | C A T C T T C T G T T T C T T C |
| *MRC5* | Low | AT5G40910.1 | 16395507 | 16399129 | + | 0 | 0 | 0 |  |
| *MRC5* | Low | AT5G41740.2 | 16688626 | 16693119 | + | 563 | 593 | 31 | T G G C T T C T T C T T C T T C T T C A T C T T C T T C G T C |
| *MRC5* | Low | AT5G41750.1 | 16693909 | 16698943 | + | 643 | 664 | 22 | C T T T G T C T T C T T C T T T G T C T T G |
| *MRC5* | Low | AT5G43470.1 | 17462611 | 17467448 | - | 0 | 0 | 0 |  |
| *MRC5* | Low | AT5G43730.1 | 17560179 | 17562929 | + | 0 | 0 | 0 |  |
| *MRC5* | Low | AT5G43740.1 | 17564738 | 17568802 | + | 0 | 0 | 0 |  |
| *MRC5* | Low | AT5G44870.1 | 18114666 | 18118608 | + | 0 | 0 | 0 |  |
| *MRC5* | Low | AT5G45050.1 | 18176914 | 18181805 | - | 0 | 0 | 0 |  |
| *MRC5* | Low | AT5G45060.1 | 18182038 | 18186495 | + | 0 | 0 | 0 |  |
| *MRC5* | Low | AT5G45070.1 | 18187630 | 18189431 | - | 571 | 589 | 19 | T G G C T G C T T C T T C T T C T G T |
| *MRC5* | Low | AT5G45080.1 | 18191575 | 18193207 | - | 502 | 517 | 16 | T G G C T T C T T C T T C C T C |
| *MRC5* | Low | AT5G45090.1 | 18202753 | 18205000 | - | 115 | 133 | 19 | T T T C T T C T T C T T C T T C C A A |
| *MRC5* | Low | AT5G45090.1 | 18202753 | 18205000 | - | 910 | 937 | 28 | C T T C T A C C T T T T C T T C G T C G T C T T C T T T |
| *MRC5* | Low | AT5G45200.1 | 18283967 | 18290332 | - | 0 | 0 | 0 |  |
| *MRC5* | Low | AT5G45210.1 | 18295521 | 18298434 | + | 0 | 0 | 0 |  |
| *MRC5* | Low | AT5G45220.1 | 18298926 | 18301069 | - | 511 | 529 | 19 | T A T C C T C T T C T T C T T C G G T |
| *MRC5* | Low | AT5G45230.1 | 18302147 | 18308303 | - | 0 | 0 | 0 |  |
| *MRC5* | Low | AT5G45240.1 | 18313706 | 18319089 | + | 0 | 0 | 0 |  |
| *MRC5* | Low | AT5G45250.1 | 18321914 | 18326022 | - | 0 | 0 | 0 |  |
| *MRC5* | Low | AT5G45260.1 | 18326203 | 18332609 | + | 0 | 0 | 0 |  |
| *MRC5* | Low | AT5G45440.1 | 18412308 | 18413555 | - | 485 | 500 | 16 | C T T C T T C T G T T G C T T C |
| *MRC5* | Low | AT5G45490.1 | 18431003 | 18432397 | + | 515 | 533 | 19 | T T T C T T C A T C A T C T T C T T T |
| *MRC5* | Low | AT5G45510.1 | 18444657 | 18449446 | + | 0 | 0 | 0 |  |
| *MRC5* | Low | AT5G46450.1 | 18835618 | 18839546 | + | 505 | 523 | 19 | C T T C T T C G T C C T C C T C T T C |
| *MRC5* | Low | AT5G46470.1 | 18842701 | 18849741 | + | 502 | 526 | 25 | T G G C T T C T T C T T C C T C C T C C T C T T C |
| *MRC5* | Low | AT5G46490.2 | 18850776 | 18853843 | + | 574 | 595 | 22 | T G G C T T C T T C A T C T T C T T C T C G |
| *MRC5* | Low | AT5G46500.1 | 18856454 | 18857787 | + | 0 | 0 | 0 |  |
| *MRC5* | Low | AT5G46510.1 | 18860451 | 18867013 | + | 0 | 0 | 0 |  |
| *MRC5* | Low | AT5G46520.1 | 18867767 | 18872415 | + | 575 | 596 | 22 | T G G C T T C T T C A T C T T C T T C T C G |
| *MRC5* | Low | AT5G47250.1 | 19185794 | 19188745 | - | 0 | 0 | 0 |  |
| *MRC5* | Low | AT5G47260.1 | 19189411 | 19192516 | + | 0 | 0 | 0 |  |
| *MRC5* | Low | AT5G47280.1 | 19193157 | 19195559 | + | 0 | 0 | 0 |  |
| *MRC5* | Low | AT5G48620.1 | 19716476 | 19721452 | + | 0 | 0 | 0 |  |
| *MRC5* | Low | AT5G48770.1 | 19773277 | 19777242 | - | 508 | 523 | 16 | C T T C G T C T T T G T C T T C |
| *MRC5* | Low | AT5G48780.1 | 19777468 | 19779604 | + | 545 | 566 | 22 | T G T C T T C C T C T T C T T C C T C T T C |
| *MRC5* | Low | AT5G49140.1 | 19919085 | 19923415 | - | 0 | 0 | 0 |  |

[1–7]**.**
