# Supplementary material for: Recombination Rate Heterogeneity within Arabidopsis Disease Resistance Genes
Source: PLoS Genet. 2016 Jul 14;12(7):e1006179. doi: 10.1371/journal.pgen.1006179 (PMC4945094; doi:10.1371/journal.pgen.1006179)
Supplement: S18 Table — The first column lists the accession the I5a reporter (Col-0) was crossed to. In the case of Col-0 this represents data from Col-0/Col-0 homozygotes. The total number of pollen counted is listed, in addition to the number with red fluorescence alone (Red), yellow alone (yellow), both colors (Red+Yellow), or neither fluorescence (None). Genetic distance is calculated as cM = 100 × (Y/(Y+RY)). Where Y is the number of yellow alone pollen grains and RG is the number of both yellow and red fluorescent pollen grains. To test whether recombinant and non-recombinant counts were significantly different between replicate groups we used a generalized linear model (GLM), assuming that the count data is binomially distributed. Tests were performed between F1 genotypes and Col/Col homozygotes. (DOCX) [file pgen.1006179.s024.docx]

**S18 Table. Measurement of *I5a* genetic distance using fluorescent pollen and flow cytometry.**

| Col/Accession | Total | Red | Red+Yellow | None | Yellow | cM | GLM *P* |
| --- | --- | --- | --- | --- | --- | --- | --- |
| No-0 | 30,000 | 2,721 | 10,424 | 14,598 | 2,257 | 17.8 |  |
| No-0 | 30,000 | 2,627 | 10,325 | 14,794 | 2,254 | 17.92 |  |
| No-0 | 30,000 | 3,009 | 11,342 | 13,437 | 2,212 | 16.32 |  |
| No-0 | 30,000 | 2,932 | 10,493 | 14,280 | 2,295 | 17.95 |  |
| No-0 | 30,000 | 2,891 | 10,208 | 14,732 | 2,169 | 17.52 |  |
| No-0 | 30,000 | 2,788 | 10,306 | 14,777 | 2,129 | 17.12 | <2.0x10^-16^ |
| Wil-2 | 30,000 | 3,152 | 10,791 | 13,513 | 2,544 | 19.08 |  |
| Wil-2 | 30,000 | 3,056 | 10,571 | 13,882 | 2,491 | 19.07 |  |
| Wil-2 | 30,000 | 2,788 | 10,308 | 14,819 | 2,085 | 16.82 |  |
| Wil-2 | 25,854 | 2,399 | 8,665 | 13,147 | 1,643 | 15.94 |  |
| Wil-2 | 30,000 | 3,100 | 9,841 | 14,894 | 2,165 | 18.03 |  |
| Wil-2 | 30,000 | 2,886 | 9,128 | 16,018 | 1,968 | 17.74 | <2.0x10^-16^ |
| Kn-0 | 30,000 | 3,729 | 10,832 | 13,104 | 2,335 | 17.73 |  |
| Kn-0 | 30,000 | 3,507 | 10,148 | 13,934 | 2,411 | 19.2 |  |
| Kn-0 | 30,000 | 3,629 | 10,701 | 13,426 | 2,244 | 17.33 |  |
| Kn-0 | 30,000 | 3,536 | 10,469 | 13,622 | 2,373 | 18.48 |  |
| Kn-0 | 30,000 | 3,775 | 10,726 | 13,257 | 2,242 | 17.29 |  |
| Kn-0 | 30,000 | 3,633 | 10,283 | 13,691 | 2,393 | 18.88 | <2.0x10^-16^ |
| Kond | 30,000 | 2,873 | 9,643 | 15,414 | 2,070 | 17.67 |  |
| Kond | 30,000 | 2,729 | 9,195 | 16,017 | 2,059 | 18.3 |  |
| Kond | 30,000 | 3,004 | 9,006 | 16,060 | 1,930 | 17.65 |  |
| Kond | 30,000 | 2,821 | 8,827 | 16,382 | 1,970 | 18.25 |  |
| Kond | 30,000 | 2,854 | 8,150 | 17,119 | 1,877 | 18.72 |  |
| Kond | 30,000 | 2,761 | 7,641 | 17,867 | 1,731 | 18.47 | <2.0x10^-16^ |
| Ler-0 | 30,000 | 2,800 | 10,130 | 14,985 | 2,085 | 17.07 |  |
| Ler-0 | 30,000 | 2,768 | 9,859 | 15,217 | 2,156 | 17.94 |  |
| Ler-0 | 30,000 | 3,069 | 9,948 | 14,646 | 2,337 | 19.02 |  |
| Ler-0 | 30,000 | 3,063 | 9,440 | 15,131 | 2,366 | 20.04 |  |
| Ler-0 | 30,000 | 2,717 | 10,238 | 14,842 | 2,203 | 17.71 |  |
| Ler-0 | 30,000 | 2,736 | 9,850 | 15,262 | 2,152 | 17.93 | <2.0x10^-16^ |
| Wu-0 | 30,000 | 3,370 | 9,457 | 14,988 | 2,185 | 18.77 |  |
| Wu-0 | 30,000 | 3,226 | 8,959 | 15,634 | 2,181 | 19.58 |  |
| Wu-0 | 30,000 | 3,316 | 9,651 | 14,891 | 2,142 | 18.16 |  |
| Wu-0 | 30,000 | 3,209 | 9,097 | 15,638 | 2,056 | 18.43 |  |
| Wu-0 | 30,000 | 3,546 | 9,380 | 14,899 | 2,175 | 18.82 |  |
| Wu-0 | 30,000 | 3,483 | 8,838 | 15,486 | 2,193 | 19.88 | <2.0x10^-16^ |
| Rsch-4 | 30,000 | 3,961 | 8,796 | 15,269 | 1,974 | 18.33 |  |
| Rsch-4 | 30,000 | 3,874 | 8,537 | 15,629 | 1,960 | 18.67 |  |
| Rsch-4 | 30,000 | 3,883 | 8,167 | 16,088 | 1,862 | 18.57 |  |
| Rsch-4 | 30,000 | 3,781 | 8,215 | 16,056 | 1,948 | 19.17 |  |
| Rsch-4 | 30,000 | 3,606 | 8,797 | 15,493 | 2,104 | 19.3 |  |
| Rsch-4 | 30,000 | 3,622 | 8,820 | 15,371 | 2,187 | 19.87 | <2.0x10^-16^ |
| Kas | 30,114 | 3,233 | 10,686 | 13,834 | 2,361 | 18.1 |  |
| Kas | 30,445 | 3,245 | 10,380 | 14,320 | 2,500 | 19.41 |  |
| Kas | 30,415 | 3,509 | 9,138 | 15,402 | 2,366 | 20.57 |  |
| Kas | 30,000 | 3,295 | 8,952 | 15,553 | 2,200 | 19.73 |  |
| Kas | 30,000 | 3,457 | 9,737 | 14,388 | 2,418 | 19.89 |  |
| Kas | 24,576 | 2,610 | 7,329 | 12,820 | 1,817 | 19.87 | <2.0x10^-16^ |
| Edi-0 | 50,000 | 5,679 | 7,028 | 35,509 | 1,784 | 20.25 |  |
| Edi-0 | 50,000 | 7,058 | 6,072 | 35,375 | 1,495 | 19.76 |  |
| Edi-0 | 50,000 | 5,906 | 8,383 | 33,604 | 2,107 | 20.09 |  |
| Edi-0 | 60,000 | 6,923 | 11,252 | 39,274 | 2,551 | 18.48 |  |
| Edi-0 | 60,000 | 6,798 | 12,082 | 38,509 | 2,611 | 17.77 |  |
| Edi-0 | 60,000 | 7,872 | 12,391 | 36,278 | 3,459 | 21.82 | <2.0x10^-16^ |
| Ct-1 | 30,000 | 3,768 | 9,305 | 14,582 | 2,345 | 20.13 |  |
| Ct-1 | 30,000 | 3,614 | 8,470 | 15,677 | 2,239 | 20.91 |  |
| Ct-1 | 30,000 | 3,866 | 8,497 | 15,590 | 2,047 | 19.41 |  |
| Ct-1 | 30,000 | 3,770 | 8,230 | 15,951 | 2,049 | 19.93 |  |
| Ct-1 | 30,000 | 4,008 | 7,630 | 16,494 | 1,868 | 19.67 |  |
| Ct-1 | 30,000 | 4,001 | 6,955 | 17,217 | 1,827 | 20.8 | 2.16x10^-14^ |
| C24 | 30,000 | 3,484 | 8,823 | 15,513 | 2,180 | 19.81 |  |
| C24 | 30,000 | 3,416 | 8,930 | 15,444 | 2,210 | 19.84 |  |
| C24 | 30,000 | 3,320 | 9,119 | 15,205 | 2,356 | 20.53 |  |
| C24 | 30,000 | 3,503 | 8,893 | 15,325 | 2,279 | 20.4 |  |
| C24 | 30,000 | 3,478 | 8,971 | 15,271 | 2,280 | 20.26 |  |
| C24 | 30,000 | 3,450 | 8,940 | 15,359 | 2,251 | 20.11 | 1.38x10^-14^ |
| Can-0 | 50,000 | 6,136 | 10,701 | 30,202 | 2,961 | 21.67 |  |
| Can-0 | 50,000 | 5,963 | 10,201 | 30,939 | 2,897 | 22.12 |  |
| Can-0 | 50,000 | 6,615 | 11,211 | 29,042 | 3,132 | 21.84 |  |
| Can-0 | 38,577 | 4,892 | 8,229 | 23,135 | 2,321 | 22 |  |
| Can-0 | 40,149 | 5,078 | 8,351 | 24,860 | 1,860 | 18.22 |  |
| Can-0 | 28,827 | 3,391 | 5,286 | 18,985 | 1,165 | 18.06 | 9.68x10^-05^ |
| Bay-0 | 30,000 | 3,159 | 10,360 | 13,832 | 2,649 | 20.36 |  |
| Bay-0 | 30,000 | 3,229 | 10,053 | 14,006 | 2,712 | 21.25 |  |
| Bay-0 | 30,000 | 3,207 | 10,197 | 13,976 | 2,620 | 20.44 |  |
| Bay-0 | 30,000 | 3,188 | 9,945 | 14,099 | 2,768 | 21.77 |  |
| Bay-0 | 30,000 | 3,144 | 10,171 | 14,010 | 2,675 | 20.82 |  |
| Bay-0 | 30,000 | 3,253 | 9,881 | 14,192 | 2,674 | 21.3 | 6.12x10^-05^ |
| Sha | 30,000 | 3,119 | 8,828 | 15,877 | 2,176 | 19.77 |  |
| Sha | 30,000 | 3,092 | 8,457 | 16,165 | 2,286 | 21.28 |  |
| Sha | 30,000 | 3,017 | 9,664 | 14,688 | 2,631 | 21.4 |  |
| Sha | 30,000 | 2,878 | 9,403 | 15,175 | 2,544 | 21.29 |  |
| Sha | 30,000 | 3,092 | 8,981 | 15,398 | 2,529 | 21.97 |  |
| Sha | 30,000 | 3,049 | 8,976 | 15,445 | 2,530 | 21.99 | 1.37x10^-02^ |
| Oy-0 | 30,000 | 3,462 | 8,793 | 15,356 | 2,389 | 21.36 |  |
| Oy-0 | 30,000 | 3,470 | 8,732 | 15,323 | 2,475 | 22.08 |  |
| Oy-0 | 30,000 | 3,672 | 8,332 | 15,642 | 2,354 | 22.03 |  |
| Oy-0 | 30,000 | 3,744 | 8,262 | 15,729 | 2,265 | 21.52 |  |
| Oy-0 | 30,000 | 3,716 | 8,869 | 15,019 | 2,396 | 21.27 |  |
| Oy-0 | 30,000 | 3,687 | 8,629 | 15,187 | 2,497 | 22.44 | 0.826 |
| Col-0 | 30,000 | 3,534 | 9,608 | 14,242 | 2,616 | 21.4 |  |
| Col-0 | 30,000 | 3,535 | 9,469 | 14,225 | 2,771 | 22.64 |  |
| Col-0 | 30,000 | 3,386 | 9,831 | 14,082 | 2,701 | 21.55 |  |
| Col-0 | 30,000 | 3,398 | 9,556 | 14,321 | 2,725 | 22.19 |  |
| Col-0 | 30,000 | 3,411 | 10,043 | 13,850 | 2,696 | 21.16 |  |
| Col-0 | 30,000 | 3,372 | 9,716 | 14,159 | 2,753 | 22.08 | - |
| Po-0 | 30,503 | 3,828 | 9,607 | 14,243 | 2,825 | 22.72 |  |
| Po-0 | 30,000 | 3,757 | 9,091 | 14,417 | 2,735 | 23.13 |  |
| Po-0 | 30,000 | 3,667 | 9,388 | 14,429 | 2,516 | 21.14 |  |
| Po-0 | 30,000 | 3,570 | 9,154 | 14,714 | 2,562 | 21.87 |  |
| Po-0 | 30,000 | 3,521 | 9,879 | 13,742 | 2,858 | 22.44 |  |
| Po-0 | 30,000 | 3,719 | 9,294 | 14,262 | 2,725 | 22.67 | 2.08x10^-02^ |
| Cvi-0 | 30,000 | 3,657 | 9,664 | 13,819 | 2,860 | 22.84 |  |
| Cvi-0 | 30,000 | 3,667 | 9,502 | 13,886 | 2,945 | 23.66 |  |
| Cvi-0 | 30,000 | 3,626 | 9,211 | 14,364 | 2,799 | 23.31 |  |
| Cvi-0 | 30,000 | 3,639 | 8,874 | 14,672 | 2,815 | 24.08 |  |
| Cvi-0 | 30,000 | 3,323 | 9,503 | 14,405 | 2,769 | 22.56 |  |
| Cvi-0 | 30,000 | 3,256 | 9,412 | 14,666 | 2,666 | 22.07 | 9.13x10^-09^ |
| Sf-2 | 30,000 | 4,139 | 7,680 | 15,889 | 2,292 | 22.98 |  |
| Sf-2 | 30,000 | 4,082 | 7,369 | 16,199 | 2,350 | 24.18 |  |
| Sf-2 | 30,000 | 3,890 | 7,170 | 16,665 | 2,275 | 24.09 |  |
| Sf-2 | 30,000 | 3,941 | 7,217 | 16,511 | 2,331 | 24.41 |  |
| Sf-2 | 30,000 | 4,147 | 7,265 | 16,220 | 2,368 | 24.58 |  |
| Sf-2 | 30,000 | 4,121 | 7,220 | 16,420 | 2,239 | 23.67 | <2.0x10^-16^ |
