# Supplementary material for: Recombination Rate Heterogeneity within Arabidopsis Disease Resistance Genes
Source: PLoS Genet. 2016 Jul 14;12(7):e1006179. doi: 10.1371/journal.pgen.1006179 (PMC4945094; doi:10.1371/journal.pgen.1006179)
Supplement: S19 Table — Positions annealing to Col/Ler polymorphic bases in the allele-specific primers are indicated in red. The accession each primer anneals to is listed (Col or Ler), together with a code indicating whether the primer is forward or reverse (F or R) and used in the first or second round of allele-specific amplification (1 or 2). (DOCX) [file pgen.1006179.s025.docx]

**S19 Table. *RAC1* primer sequences used for allele-specific PCR amplification.**

| Name | Accession/Primer | Sequence (5'-3') | Coordinates (TAIR10) |
| --- | --- | --- | --- |
| KC459 | Ler F1 | CTGACTTGAGTGATCGCAA | 11,285,672 – 11,285,690 |
| KC418 | Col R1 | ATTTCACCCGATGTAGTCC | 11,298,007 – 11,298,025 |
| KC465 | Ler F2 | GTGGCCGCAAGCAAAAATAT | 11,288,146 – 11,288,165 |
| KC417 | Col R2 | TAGTTTTTCTGACCCCAC | 11,297,548 – 11,297,565 |
| KC493 | Col F1 | AAAACGTGCAACCTAAGAAC | 11,288,566 – 11,288,585 |
| KC495 | Col F2 | AACAGATTGGTCTCATTG | 11,288,932 – 11,288,949 |
